# Natural variation and dosage of the HEI10 meiotic E3 ligase control *Arabidopsis* crossover recombination

**DOI:** 10.1101/gad.295501.116

**Published:** 2017-02-01

**Authors:** Piotr A. Ziolkowski, Charles J. Underwood, Christophe Lambing, Marina Martinez-Garcia, Emma J. Lawrence, Liliana Ziolkowska, Catherine Griffin, Kyuha Choi, F. Chris H. Franklin, Robert A. Martienssen, Ian R. Henderson

**Affiliations:** 1 Department of Plant Sciences, University of Cambridge, Cambridge CB2 3EA, United Kingdom;; 2 Department of Biotechnology, Adam Mickiewicz University, 61-614 Poznan, Poland;; 3 Howard Hughes Medical Institute, Gordon and Betty Moore Foundation, Watson School of Biological Sciences, Cold Spring Harbor Laboratory, Cold Spring Harbor, New York 11724, USA;; 4 School of Biosciences, University of Birmingham, Birmingham B15 2TT, United Kingdom

**Keywords:** meiosis, recombination, modifier, HEI10, ZMM, *Arabidopsis*

## Abstract

During meiosis, homologous chromosomes undergo crossover recombination, which creates genetic diversity and balances homolog segregation. Despite these critical functions, crossover frequency varies extensively within and between species. Although natural crossover recombination modifier loci have been detected in plants, causal genes have remained elusive. Using natural *Arabidopsis thaliana* accessions, we identified two major recombination quantitative trait loci (*rQTL*s) that explain 56.9% of crossover variation in Col×Ler F_2_ populations. We mapped *rQTL1* to semidominant polymorphisms in *HEI10*, which encodes a conserved ubiquitin E3 ligase that regulates crossovers. Null *hei10* mutants are haploinsufficient, and, using genome-wide mapping and immunocytology, we show that transformation of additional *HEI10* copies is sufficient to more than double euchromatic crossovers. However, heterochromatic centromeres remained recombination-suppressed. The strongest *HEI10*-mediated crossover increases occur in subtelomeric euchromatin, which is reminiscent of sex differences in *Arabidopsis* recombination. Our work reveals that HEI10 naturally limits *Arabidopsis* crossovers and has the potential to influence the response to selection.

## Abstract (precis)

Here, Ziolkowski et al. combine high-throughput fluorescence methods to measure crossovers with natural *Arabidopsis* ecotypes in order to identify the first *trans*-acting modifier of meiotic recombination in plants. The authors found that HEI10, which encodes a conserved ubiquitin E3 ligase, naturally limits *Arabidopsis* crossovers and has the potential to influence the response to selection.

The majority of eukaryotes reproduces via the meiotic cell division, where a diploid cell replicates DNA once and segregates chromosomes twice to produce tetrads of haploid gametes (Barton and Charlesworth 1998). Genetic diversity is generated between gametes due to independent chromosome segregation in addition to recombination between homologous chromosomes during meiotic prophase I (Barton and Charlesworth 1998). Despite the importance of crossovers for balanced chromosome segregation during meiosis and fertility, extensive genetic variation in recombination frequency is observed within and between species (Sanchez-Moran et al. 2002; Coop and Przeworski 2006; Fledel-Alon et al. 2011; Hinch et al. 2011; Sandor et al. 2012; Bauer et al. 2013; Rodgers-Melnick et al. 2015; Ziolkowski et al. 2015; Johnston et al. 2016). Importantly, natural variation that modifies crossover frequency has the potential to widely influence genetic adaptation and the response to selection (Hill and Robertson 1966; Feldman et al. 1996; Barton and Charlesworth 1998).

Genetic polymorphisms that modify crossover frequency can be classified as *cis-* or *trans-*acting, according to whether they control recombination on the same chromosome or throughout the genome, respectively (Coop and Przeworski 2006; Yandeau-Nelson et al. 2006; Baudat and de Massy 2007; Ziolkowski et al. 2015). Examples of human *trans* modifier loci include the *RNF212* meiotic E3 ligase gene, which controls crossover levels (Kong et al. 2008; Fledel-Alon et al. 2011), and the *PRDM9* zinc finger SET domain gene, which specifies recombination hot spot locations (Fledel-Alon et al. 2011; Hinch et al. 2011). Polymorphisms are also known to exert local *cis* effects, where heterozygous polymorphisms can inhibit crossover repair of interhomolog strand invasion events (Borts and Haber 1987; Baudat and de Massy 2007; Mercier et al. 2015). Structural variation (for example, insertions and deletions [indels], translocations, and inversions) are also associated with crossover suppression at larger physical scales (Fransz et al. 2016). Extensive evidence for *cis* and *trans* modification of crossover frequency exists in plants, including *Arabidopsis thaliana* (Timmermans et al. 1997; Barth et al. 2001; Yandeau-Nelson et al. 2006; Esch et al. 2007; McMullen et al. 2009; López et al. 2012; Salomé et al. 2012; Bauer et al. 2013; Martín et al. 2014; Rodgers-Melnick et al. 2015; Ziolkowski et al. 2015). Therefore, we sought to use high-throughput fluorescent reporter systems to measure recombination and identify *trans*-acting crossover modifier loci that vary between *A. thaliana* accessions.

Meiotic recombination initiates via formation of DNA double-strand breaks (DSBs) by SPO11 transesterases (Mercier et al. 2015). DSBs are resected to generate ssDNA, which is bound by the RecA-related recombinases RAD51 and DMC1 (Mercier et al. 2015). The resulting nucleoprotein filaments can then invade a replicated sister chromatid or a homologous DNA duplex to form a displacement loop (Mercier et al. 2015). Immunostaining of *Arabidopsis* meiocytes for DMC1, RAD51, or the DSB-associated histone variant γH2A.X has revealed ∼100–200 foci distributed along the paired homologous chromosomes at the leptotene stage (Girard et al. 2015; Yelina et al. 2015). Approximately 10 of these initiating meiotic DSBs mature into crossovers per *Arabidopsis* meiosis (Giraut et al. 2011; Salomé et al. 2012; Rowan et al. 2015; Choi et al. 2016).

Meiotic interhomolog strand invasion intermediates can follow alternative repair fates, including crossover or noncrossover, which differ in exchange of flanking markers (Mercier et al. 2015). The majority of *Arabidopsis* crossovers is dependent on the conserved ZMM pathway (named after the budding yeast genes *Zip1*, *Zip2*, *Zip3*, *Zip4*, *Msh4*, *Msh5*, and *Mer3*), which includes *SHOC1*, *HEI10*, *ZIP4*, *MSH4*, *MSH5*, *MER3, PTD*, *MLH1*, and *MLH3* (Lynn et al. 2007; Mercier et al. 2015). Crossovers generated by the ZMM pathway show interference, where double crossovers are spaced farther apart than expected at random, which is detectable over the scale of megabases in *Arabidopsis* (Lynn et al. 2007; Mercier et al. 2015). The ZMM pathway is thought to stabilize interhomolog recombination intermediates, including double Holliday junctions, and promote crossover resolution (Lynn et al. 2007; Gray and Cohen 2016). A minority of *Arabidopsis* crossovers is generated by a noninterfering pathway that includes MUS81 (Mercier et al. 2015). Noncrossover repair of strand invasion events is promoted by multiple nonredundant pathways that include *FANCONI ANEMIA COMPLEMENTATION GROUP M* (*FANCM*), *MHF1*, *MHF2*, *FIDGETIN-LIKE1* (*FIGL1*), *RECQ4A*, *RECQ4B*, *TOPOISOMERASE3*α (*TOP3*α), and *MSH2* (Mercier et al. 2015). The combined action of these anti-crossover pathways results in repair of ∼90% of initiating meiotic DSBs as noncrossovers.

In this study, we identified an *Arabidopsis* natural genetic variation that acts in *trans* to control meiotic crossover frequency. Although *A. thaliana* is predominantly self-fertilizing, clear evidence for outcrossing exists. For example, *Arabidopsis* linkage disequilibrium decays rapidly over kilobase distances, and strong historical crossover hot spots are detectable (Kim et al. 2007; Horton et al. 2012; Choi et al. 2013). Genotyping of natural *Arabidopsis* populations has also revealed standing heterozygosity and evidence for local outcrossing between subpopulations (Bomblies et al. 2010). Therefore, recombination-modifying polymorphisms have had the opportunity to exert an effect on the genetic history of this species. Here we identify natural genetic polymorphisms in the *HEI10* meiotic E3 ligase gene that associate with quantitative variation in crossover frequency between *Arabidopsis* accessions. We further show that *HEI10* is highly dosage-sensitive and that transformation of additional *HEI10* copies is sufficient to more than double crossover recombination throughout euchromatin. Together, this demonstrates that HEI10 is a limiting factor for interference-sensitive crossover formation in *Arabidopsis*.

## Results

### Detecting recombination modifier loci using Col/Ler chromosome substitution lines (CSLs)

**Figure 1.**
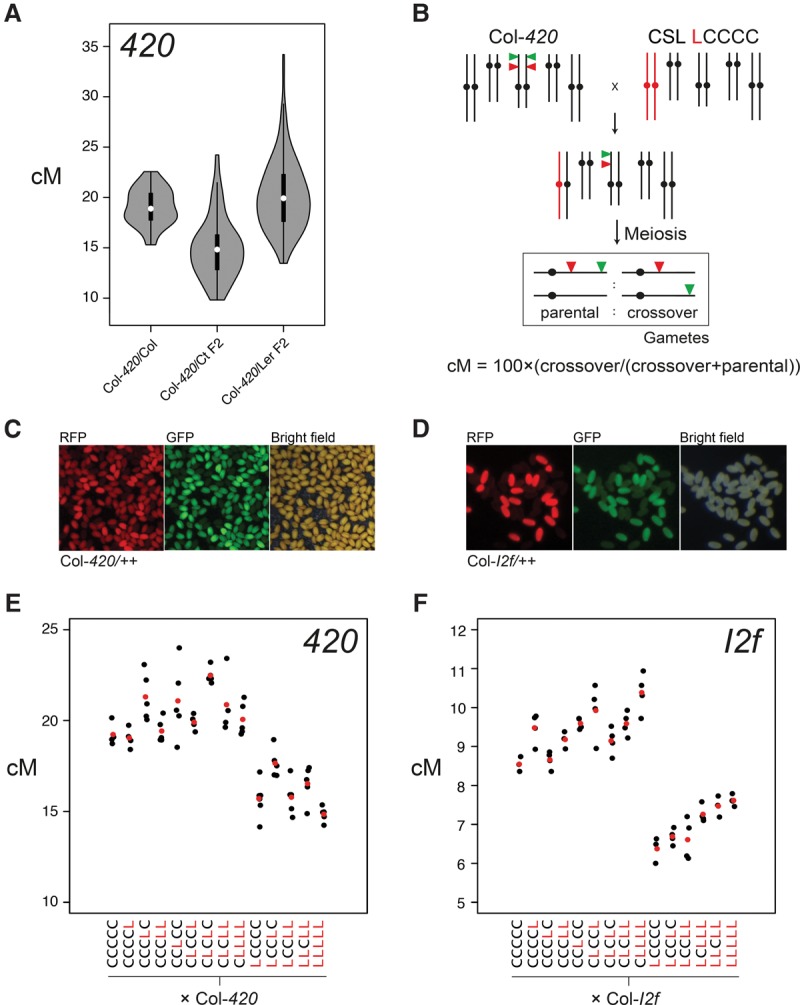
A dosage-sensitive *trans-*acting recombination modifier on *Arabidopsis* chromosome 1. (*A*) Col-*420* FTL crossover frequency (in centimorgans) in Col-*420*/Col, Col-*420*/Ct F_2_ (Ziolkowski et al. 2015), or Col-*420*/Ler F_2_ populations. (*B*) Crossing scheme used to analyze CSLs. *Arabidopsis* chromosomes are colored black (Col) or red (Ler), with FTL transgenes indicated by colored triangles. Parental and crossover fluorescence ratios were used to measure genetic distance (in centimorgans). (*C*) Representative micrographs of seeds from a *420*/++ hemizygote imaged under bright-field or showing green and red fluorescence. (*D*) As for *C*, but showing pollen from an *I2f*/++ hemizygote. (*E*) *420* crossover frequency in F_1_ plants derived from Col-*420*×CSL crosses. CSL genotypes are indicated by “C” (Col) and “L” (Ler) for each chromosome. Replicate F_1_ data are shown by black dots, and mean values are indicated by red dots. (*F*) As for *E*, but measuring crossover frequency (in centimorgans) within FTL interval *I2f*.

Genetic segregation of linked, hemizygous T-DNAs expressing different colors of fluorescent proteins in pollen or seed (fluorescent-tagged lines [FTLs]) can be used to measure *Arabidopsis* crossover frequency (Fig. 1; Emmanuel et al. 2006; Berchowitz and Copenhaver 2008; Yelina et al. 2013; Ziolkowski et al. 2015). We previously analyzed crossovers in an F_2_ population derived from crosses between the Col-*420* subtelomeric FTL and Catania-1 (Ct) parents, which did not identify significant *trans*-acting recombination modifier loci (Ziolkowski et al. 2015). To further screen for natural crossover modifiers, we generated a Col-*420*×Landsberg *erecta* (Ler) F_2_ population, which showed higher mean recombination than Col-*420*×Ct (20.2 cM vs. 15.0 cM) (Fig. 1A; Supplemental Tables S1, S2) and significantly greater variation in crossover frequency between individuals (Brown-Forsythe test, *P* = 2.91 × 10^−32^). This is consistent with the presence of *trans* modifier loci (Fig. 1A; Supplemental Tables S1, S2).

To identify *trans* recombination modifier loci, we first used Col/Ler CSLs (Fig. 1B; Wijnker et al. 2012). For example, CSL LCCCC denotes Ler (L) and Col (C) genotypes for each of the five chromosomes (Fig. 1B). Fourteen CSLs were crossed to Col-*I2f* and Col-*420* FTLs (crossover reporters located on chromosomes 2 and 3, respectively), and replicate F_1_ measurements were collected (Fig. 1B–F; Supplemental Tables S3, S4). We observed that all F_1_ genotypes that were chromosome 1 Col/Ler heterozygous showed significantly reduced crossovers compared with control Col-*420*×CCCCC F_1_ plants, with weaker effects detected from the other chromosomes (Fig. 1E,F; Supplemental Tables S3–S5). This reveals the presence of a semidominant *trans*-acting recombination modifier on chromosome 1.

### Genetic mapping of *Arabidopsis* recombination quantitative trait loci (rQTLs)

**Figure 2.**
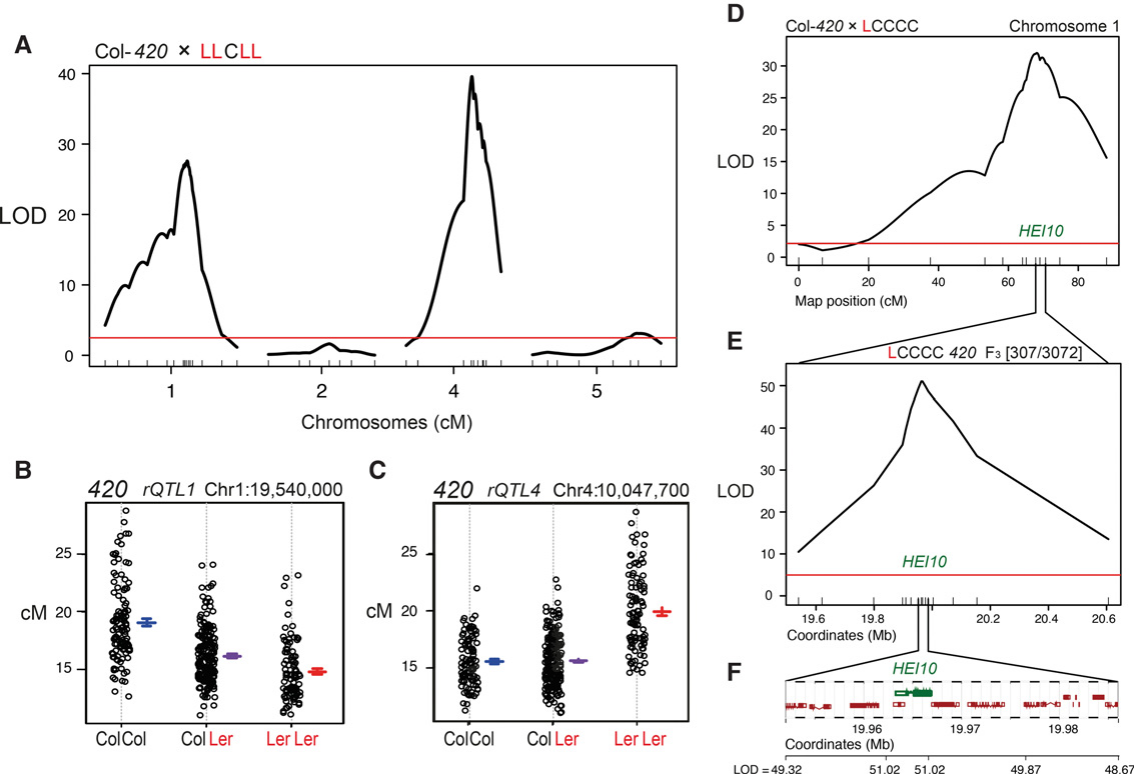
The *rQTL1* recombination modifier locus maps to the meiotic E3 ligase gene *HEI10*. (*A*) LOD scores for genetic markers and crossover frequency from a Col-*420*×LLCLL F_2_ population. Genetic map positions (in centimorgans) of markers are indicated on the *X*-axis. The red line indicates the 95% significance threshold. (*B*) Effects plots showing *420* crossovers (in centimorgans) from Col/Col, Col/Ler, or Ler/Ler individuals for a *rQTL1* maker. (*C*) As for *B*, but showing a *rQTL4* marker. (*D*) LOD scores for genetic markers and crossover frequency in a Col-*420*×LCCCC F_2_ population. The red line indicates the 95% significance threshold. The approximate position of *HEI10* is labeled. (*E*) As for *D*, but showing the marker LOD for *420* crossover frequency in a 1.7-Mb interval from a F_3_ Col-*420*×LCCCC population derived from *D*. (*F*) Marker LOD associated with *420* crossovers in proximity to *HEI10* (green) and adjacent genes (red).

We observed previously that juxtaposition of homozygous and heterozygous regions can influence recombination in *cis* at the megabase scale (Ziolkowski et al. 2015). To eliminate *cis* effects and specifically map *trans* recombination modifiers, we generated an F_2_ population from a Col-*420*×LLCLL cross. In this population, chromosome 3 is Col/Col homozygous, which is where the *420* FTL interval is located, and therefore *cis* effects were excluded. We identified two major *trans rQTL*s on chromosomes 1 and 4, with logarithm of the (base 10) odds ratio (LOD) scores of 40.2 and 53.5, which explain 23.3% and 33.6% of the variance in recombination, respectively (*F*-test, *P* < 2 × 10^−16^) (Fig. 2A; Supplemental Table S6). *rQTL1*^*Ler*^ genotypes associate with low recombination, with heterozygotes showing intermediate crossover frequency (Fig. 2B), consistent with the semidominant effects observed for chromosome 1 in the CSL F_1_ experiments (Fig. 1E,F; Supplemental Tables S3, S4). In contrast, *rQTL4*^*Ler*^ associates with high recombination and behaves recessively, explaining why it was not detected in the CSL experiments (Fig. 2C).

To investigate the influence of *rQTL1* and *rQTL4* on meiotic recombination elsewhere in the genome, we performed cytogenetic analysis in Col and Ler in addition to recombinants with low (*rQTL1*^*Ler*^ *rQTL4*^*Col*^) or high (*rQTL1*^*Col*^ *rQTL4*^*Ler*^) *420* crossovers (Supplemental Fig. S1A–E; Supplemental Tables S7–S9). MLH1 foci occurring along the meiotic synaptonemal complex (visualized by ZYP1 immunostaining) serve as a measure of total interfering crossovers per nucleus (Lambing et al. 2015). We observed significantly more MLH1 foci in *rQTL1*^*Col*^ *rQTL4*^*Ler*^ lines compared with the other genotypes (Mann-Whitney-Wilcoxon test, *P* = 0.0396) (Supplemental Fig. S1A,C; Supplemental Table S7). We confirmed the same trend via analysis of chiasmata at metaphase I (Mann-Whitney-Wilcoxon test, *P* = 2.20 × 10^−5^) (Supplemental Fig. S1B,D; Supplemental Table S8; Sanchez-Moran et al. 2002). These analyses confirm that Col and Ler polymorphisms underlying *rQTL1* and *rQTL4* influence crossovers not only in the *420* interval but throughout the chromosomes.

### Genetic variation in the *HEI10* meiotic E3 ligase gene underlies *rQTL1*

We sought to identify *rQTL1* using an F_2_ population derived from a Col-*420*×LCCCC cross, which again revealed a major *rQTL* on chromosome 1 (Fig. 2D; Supplemental Table S10). We selected an F_2_ individual that was Col/Ler heterozygous spanning *rQTL1* (19.54–21.24 Mb), which was self-fertilized to generate a large F_3_ population (*n* = 3072) (Fig. 2E; Supplemental Table S11). Genotyping identified 307 F_3_ plants with crossovers within the *rQTL1* region, which were then measured for *420* crossover frequency and genotyped for 15 additional markers (Fig. 2E; Supplemental Table S11). This narrowed the credible *rQTL1* interval to a 34-kb region containing 14 genes (Fig. 2E,F). The most strongly associated marker pair (LOD = 51.02) defined a 4.3-kb interval containing two genes: *MRD1* (At1g53480) and *HEI10* (At1g53490) (Fig. 2F). As *HEI10* belongs to a conserved gene family, which encodes RING domain SUMO/ubiquitin E3 ligases that promote crossovers in diverse eukaryotes, this was the strongest candidate gene for *rQTL1* (Supplemental Figs. S2, S3; Bhalla et al. 2008; Kong et al. 2008; Fledel-Alon et al. 2011; Chelysheva et al. 2012; Sandor et al. 2012; Serrentino et al. 2013; De Muyt et al. 2014; Qiao et al. 2014; Johnston et al. 2016; Rao et al. 2017). HEI10 family proteins possess N-terminal RING domains, central coiled-coil domains, and C-terminal regions of unknown function (Supplemental Figs. S2, S3B; Gray and Cohen 2016).

**Figure 3.**
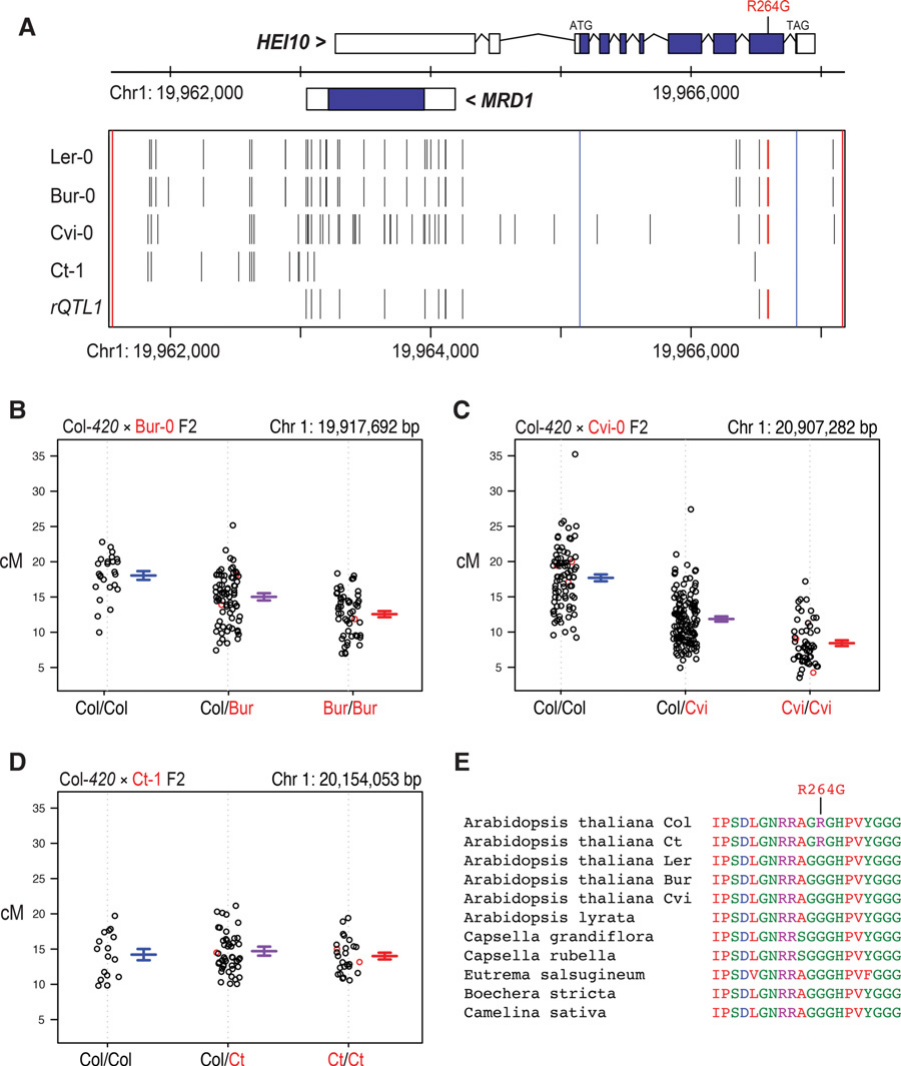
Candidate *rQTL1* Col/Ler polymorphisms. (*A*) Plot showing the *HEI10* region on chromosome 1. The positions of *HEI10* (forward strand) and *MRD1* (reverse strand) gene annotations are plotted as black boxes, with coding regions shown in blue. Blue vertical lines indicate *HEI10* ATG and TAG codons. Black *X*-axis ticks show the positions of Ler-0, Bur-0, Cvi-0, and Ct-1 polymorphisms, identified by Sanger sequencing. Red ticks show the nonsynonymous *HEI10* substitution R264G. (*B*) *420* recombination rate (in centimorgans) from individual plants in a Col-*420*×Bur-0 F_2_ population, according to the Col-0/Bur-0 genotype at marker 19,917,692 base pairs (bp). (*C*) As for *B* but plotting for marker 20,907,282 bp in a Col-*420*×Cvi-0 F_2_ population. (*D*) As for *B* but plotting for marker 20,154,053 bp in a Col-*420*×Ct-1 F_2_ population (the marker is below the 95% significance threshold LOD = 2.27). (*E*) A multiple sequence alignment of *Brassicacea* HEI10 orthologs in the region of the R264G substitution.

To further investigate *HEI10* polymorphisms associated with *rQTL1*, we sequenced the Ler accession used in our experiments and identified a single nonsynonymous (R264G) substitution and three synonymous intragenic variants relative to the Col reference sequence (Fig. 3A; Supplemental Table S12), which was consistent with 1001 Genomes project data from the closely related Ler-1 and La-0 accessions (Alonso-Blanco et al. 2016). The *HEI10* promoter, which overlaps the antisense gene *MRD1*, is also polymorphic, with 26 single-nucleotide polymorphisms (SNPs) or indels upstream of the start codon (Fig. 3A; Supplemental Table S12). We generated additional F_2_ populations derived from Col*-420*×Bur-0 or Col*-420*×Cvi-0 crosses and again observed significant association between the *HEI10* region and crossover frequency (Bur-0 LOD = 8.30, 95% significance threshold LOD = 2.82; Cvi-0 LOD = 31.74, 95% significance threshold LOD = 2.97) (Fig. 3B,C; Supplemental Table S13). We sequenced *HEI10*^*Bur*^ and *HEI10*^*Cvi*^ and observed 43 and 30 polymorphisms, respectively, relative to *HEI10*^*Col*^ (Fig. 3A; Supplemental Table S12). As we previously observed an absence of *trans rQTLs* in Col-*420*×Ct-1 populations (Fig. 3D; Ziolkowski et al. 2015), we also sequenced *HEI10*^*Ct*^ (Fig. 3A; Supplemental Table S12). Twelve polymorphisms are shared between *HEI10*^*Ler*^, *HEI10*^*Cvi*^, and *HEI10*^*Bur*^ but absent from *HEI10*^*Ct*^, including the R264 substitution, which we consider as candidates for *rQTL1* causal variants (Fig. 3A; Supplemental Table S12). *HEI10* transcript and protein levels measured by quantitative RT–PCR (qRT–PCR) and immunocytogenetic analysis, respectively, did not show significant differences between Col, Ler, and Col/Ler F_1_ (Supplemental Fig. S1E; Supplemental Tables S14–S16), consistent with the causal *rQTL1* polymorphism influencing HEI10 protein function or expression timing rather than expression level. The nonsynonymous R264G polymorphism occurs in the HEI10 C-terminal region (Fig. 3E; Supplemental Figs. S2, S3), which, by analogy with other RING E3 ligases, may play a role in substrate recognition (Deshaies and Joazeiro 2009). We queried 1001 Genomes project data for the frequency and geographic distribution of the *HEI10* R264G variants (Supplemental Fig. S4; Alonso-Blanco et al. 2016). Both alleles are globally distributed, with the majority of accessions (959 out of 1008) showing the Ler G264 genotype, and the Col R264 reference allele present in the remaining 123 (11.4%) (Supplemental Fig. S4). As other *Brassicaceae* HEI10 orthologs show glycine at position 264, this is consistent with the R264 variant observed in Col-0 and Ct-1 being more recently derived within *A. thaliana* (Fig. 3E).

### *Arabidopsis* crossover frequency is sensitive to *HEI10* dosage

**Figure 4.**
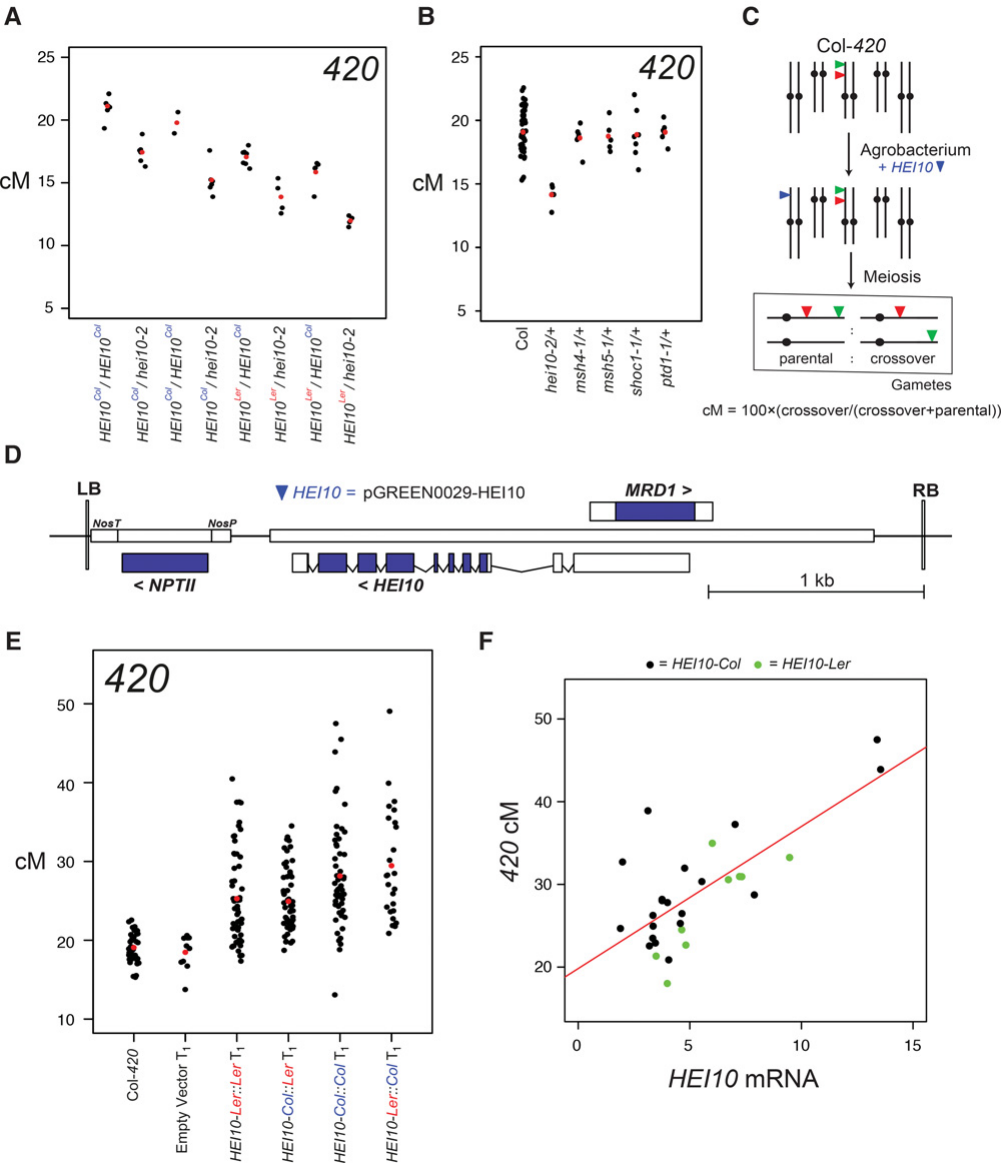
*HEI10* is a dosage-sensitive regulator of *Arabidopsis* crossovers. (*A*) *420* crossovers (in centimorgans) in F_1_ individuals derived from crosses between *HEI10*^*Col*^ or *HEI10*^*Ler*^ homozygotes and wild-type (Col) or null *hei10-2* mutants. Replicate individuals are shown as black dots, and mean values are shown as red dots. (*B*) *420* crossovers (in centimorgans) in F_1_ individuals derived from crosses with interfering crossover ZMM pathway mutants. (*C*) Schematic showing the transformation of Col-*420* with additional *HEI10* copies (blue triangles). (*D*) Diagram illustrating the *HEI10* T-DNA construct used for *Arabidopsis* transformation via *Agrobacterium*. (LB) T-DNA left border sequence; (RB) T-DNA right border sequences. (*E*) *420* crossover frequency (in centimorgans) in empty vector, *HEI10*^*Ler::Ler*^, *HEI10*^*Col::Ler*^, *HEI10*^*Ler::Col*^, and *HEI10*^*Col::Col*^ transformants compared with untransformed Col-*420*/Col controls. Data from individual plants are shown as black dots, and mean values are shown in red. (*F*) Correlation between *420* crossovers (in centimorgans) and *HEI10* transcript levels measured by qRT–PCR from *HEI10*^*Col*^ (black) and *HEI10*^*Ler*^ (green) transformant flowers. A regression line is plotted in red.

As a genetic test of *rQTL1* allelism with *HEI10*, we crossed recombinant *420* lines with the null *hei10-2* allele, which was isolated in the Col background and shows substantially reduced crossovers and fertility when homozygous (Chelysheva et al. 2012). Four independent recombinant lines were used for crosses, two of which were *rQTL1*^*Col*^ homozygous, and two that were *rQTL1*^*Ler*^ homozygous (Supplemental Table S17). All lines analyzed were *rQTL4*^*Ler*^ homozygous. F_1_ individuals that were *hei10-2* heterozygous showed significantly reduced recombination compared with wild-type crosses (Fig. 4A; Supplemental Table S17). This indicates *HEI10* dosage sensitivity, which is similar to haploinsufficiency of mouse *hei10* and *rnf212* mutations (Reynolds et al. 2013; Qiao et al. 2014). Consistent with our previous *rQTL* mapping, the progeny from *rQTL1*^*Ler*^ crosses showed significantly fewer *420* crossovers compared with *rQTL1*^*Col*^ progeny (*X*^*2*^, *P* < 2.2 × 10^−16^) (Fig. 4A; Supplemental Table S17), which we interpret as reflecting the different activity of *HEI10* Col/Ler variants, including R264G. To investigate whether haploinsufficiency is a general property of mutants in the *Arabidopsis* ZMM pathway, we compared Col*-420* F_1_ crossover measurements using *hei10-2*, *msh4-1*, *msh5-1*, *shoc1-1*, and *ptd1* heterozygotes (Fig. 4B; Supplemental Table S18). Among these mutations, only *hei10-2*/+ heterozygotes showed significantly reduced crossovers compared with wild type (*X*^*2*^, *P* = 1.63 × 10^−38^) (Fig. 4B; Supplemental Table S18), revealing that dosage sensitivity was specific to *HEI10*.

### Increased *HEI10* dosage elevates euchromatic crossovers genome-wide

Due to *HEI10* dosage sensitivity, we next investigated whether increasing copy number would elevate recombination beyond wild type (Fig. 4C–E). We transformed Col*-420* plants with a *HEI10* transgene under the control of its endogenous promoter, amplified from either Col or Ler genomic DNA (referred to here as *HEI10*^*Col*^ and *HEI10*^*Ler*^) (Fig. 4C–E). *HEI10*^*Col*^ and *HEI10*^*Ler*^ T_1_ populations, but not empty vector T_1_, showed significantly higher recombination than untransformed Col-*420* controls (Mann-Whitney-Wilcoxon test: *HEI10*^*Col*^, *P* = 4.03 × 10^−14^; *HEI10*^*Ler*^, *P* = 3.64 × 10^−10^; empty, *P* = 0.474) (Fig. 4E; Supplemental Tables S2, S19). Wide variation in recombination rate was observed within T_1_ populations (Fig. 4E), which was likely caused by varying *HEI10* transgene copy numbers and position effects that influence expression level. Indeed, qRT–PCR analysis of *HEI10* expression from meiotic stage flower buds (floral stages 1–12) of T_1_ transformants revealed a positive correlation with *420* recombination (*r* = 0.727; *P* = 7.96 × 10^−6^) (Fig. 4F; Supplemental Table S20). *HEI10*^*Col*^ transformants showed higher recombination than *HEI10*^*Ler*^ transformants (Mann-Whitney-Wilcoxon test, *P* = 8.93 × 10^−3^) (Fig. 4E; Supplemental Table S19), which is consistent with the different recombination activity of Col/Ler *HEI10* variants. To further investigate polymorphisms responsible for differences in *HEI10* function, we generated *Col::Ler* and *Ler::Col* promoter swap constructs and repeated transformation (Fig. 4E; Supplemental Table S19). The *HEI10-Ler::Col* transformants showed significantly higher recombination than *HEI10-Col::Ler* transformants (Mann-Whitney-Wilcoxon test, *P* = 1.1 × 10^−2^). Furthermore, *HEI10-Ler::Col* were not significantly different from *HEI10-Col::Col* (Mann-Whitney-Wilcoxon test, *P* = 0.841), and *HEI10-Col::Ler* were not different from *HEI10-Ler-Ler* (Mann-Whitney-Wilcoxon test, *P* = 0.259). Together, this is consistent with intragenic *HEI10* polymorphisms, including R264G, causing differences in recombination activity (Fig. 4E; Supplemental Table S19).

**Figure 5.**
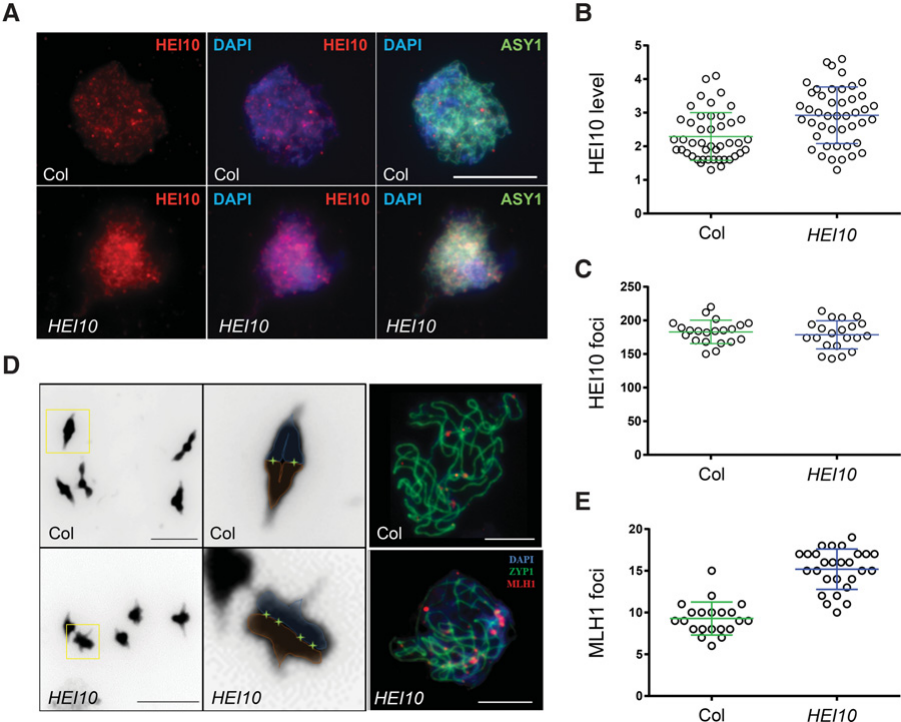
Increased *HEI10* dosage promotes formation of meiotic MLH1 foci. (*A*) Representative images showing leptotene stage male meiocytes from Col or *HEI10*^*Col*^ (line C2) immunostained for HEI10 (red) and ASY1 (green) and counterstained with DAPI (blue). Bars, 10 µm. (*B*) Quantification of HEI10 expression level via immunostaining of Col and *HEI10*^*Col*^ (line C2). (*C*) As for *B* but showing quantification of HEI10 foci. (*D*) Representative images of DAPI-stained bivalents at metaphase I in wild type (Col) (*top left* panel) and *HEI10*^*Col*^ (line C2) (*bottom left* panel). Bars, 5 µm. (*Top middle* panel) A magnified view of a wild-type ring bivalent is shown with homologs outlined in red and blue. (*Bottom middle* panel) The inferred chiasmata sites are marked with an “X.” A magnified view of a *HEI10*^*Col*^ ring bivalent is shown. Representative images showing leptotene stage male meiocytes from Col (*top right* panel) or *HEI10*^*Col*^ (line C2) (*bottom right* panel) stained for MLH1 (red), ASY1 (green), and DAPI (DNA; blue). Bars, 10 μm. (*E*) Quantification of MLH1 foci on pachytene stage meiotic chromosomes in wild type and *HEI10*^*Col*^ (line C2).

A *HEI10*^*Col*^ T_1_ line showing high *420* recombination (C2; 33.74 cM) was selected for cytological investigation. Immunostaining of leptotene stage meiotic nuclei for HEI10 showed a significant increase in signal intensity (Mann-Whitney-Wilcoxon test, *P* = 3.90 × 10^−4^), although focus numbers were not changed (Mann-Whitney-Wilcoxon test, *P* = 0.5971) (Fig. 5A–C; Supplemental Tables S21, S22). To investigate the effect of *HEI10*^*Col*^ transformation on crossover formation, we performed MLH1 immunostaining at the pachytene stage (Fig. 5D,E). There were close to double the number of MLH1 foci along *HEI10*^*Col*^ chromosomes compared with wild type (mean = wild type 9.3, *HEI10*^*Col*^ 16.2; Mann-Whitney-Wilcoxon test, *P* = 4.83 × 10^−8^) (Fig. 5D,E; Supplemental Table S23). *HEI10*^*Col*^ also showed more compact bivalents at metaphase I, which is indicative of greater crossover numbers in the chromosome arms (Fig. 5D; Sanchez-Moran et al. 2002). This provides cytological evidence that increased *HEI10* dosage and expression level elevates crossovers throughout the genome.

**Figure 6.**
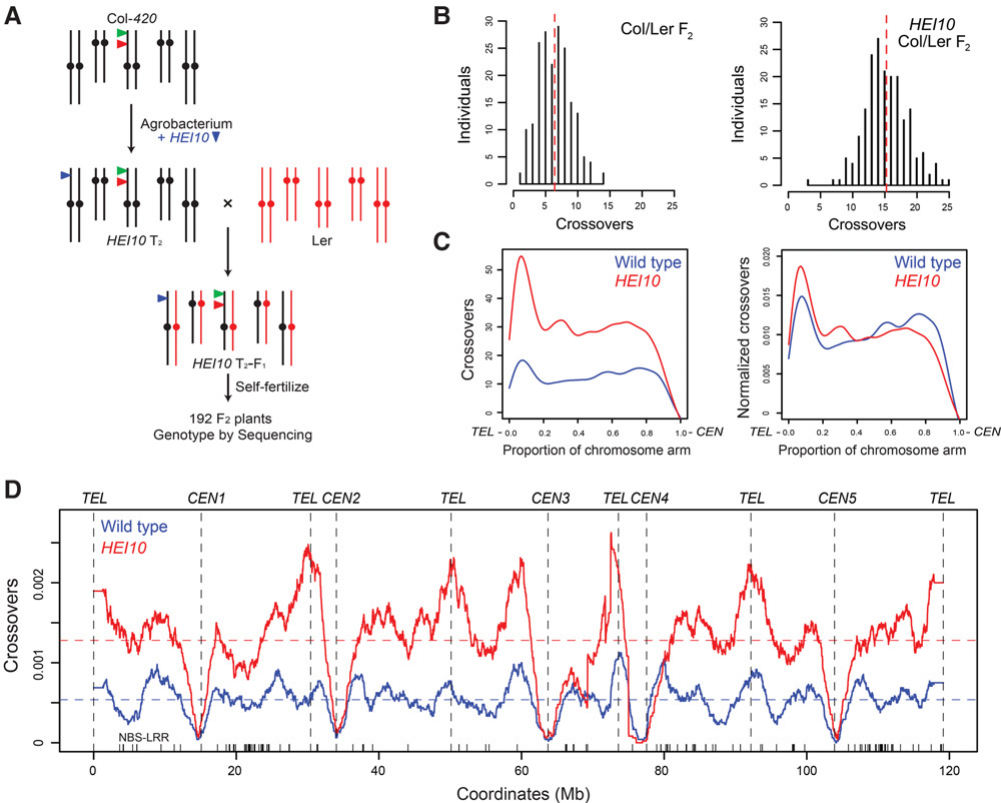
Increased *HEI10* dosage elevates euchromatic crossover frequency genome-wide. (*A*) Diagram showing genetic mapping using the *HEI10*^*Col*^ (line C2) following crosses to Ler (red). (*B*) Crossover numbers mapped by genotyping by sequencing in wild-type (Col/Ler) and *HEI10*^*Col*^/Ler F_2_ populations (Table 1). Mean values are indicated by the vertical red dotted lines. (*C*) Crossovers analyzed along the proportional length of chromosome arms from telomeres (TEL) to centromeres (CEN) in wild-type (blue) and *HEI10* (red) populations. Plots are shown analyzing total crossovers or after normalizing by total crossover events. (*D*) Crossover frequency along the five chromosomes in wild-type (blue) and *HEI10*^*Col*^ (red) populations. Mean values are shown by the dotted horizontal lines, and telomere (*TEL*) and centromere (*CEN*) positions are indicated by vertical dotted lines and labels.

To investigate the effect of increased *HEI10* dosage on crossovers at higher resolution, we used genotyping by sequencing (Fig. 6A). The *HEI10*^*Col*^ C2 line was backcrossed to Ler alongside a wild-type Col control (Fig. 6A). *HEI10*^*Col*^×Ler F_1_ plants showed highly elevated *420* recombination compared with Col/Ler F_1_ (Supplemental Tables S19, S24), demonstrating that *HEI10* increases crossovers in both hybrid (Col/Ler) and inbred (Col/Col) backgrounds. F_2_ populations (*n* = 192) were then generated from wild-type and *HEI10*^*Col*^ Col/Ler F_1_ plants and sequenced to identify crossover locations (Fig. 6A; Supplemental Table S25; Choi et al. 2016). The *HEI10*^*Col*^ population contained more than double the number of wild-type crossovers (1230 vs. 2928 crossovers; mean per F_2_, 6.41 vs. 15.25; Mann-Whitney-Wilcoxon test, *P* = 1.07 × 10^−59^) (Fig. 6B; Supplemental Fig. S5; Supplemental Table S1), consistent with our MLH1 focus analysis (Fig. 5D,E). Euchromatic chromosome arms showed the greatest increase in *HEI10*^*Col*^ crossovers (2.6×, Mann-Whitney-Wilcoxon test, *P* = 1.98 × 10^−8^), with the largest effects in the subtelomeric regions (Fig. 6C,D; Supplemental Tables S26, S27). A lower yet significant crossover increase was observed in the pericentromeres (1.6×, Mann-Whitney-Wilcoxon test, *P* = 2.34 × 10^−9^) (Fig. 6C; Supplemental Fig. S6; Supplemental Tables S26, S27). In contrast, centromeric suppression of crossovers was observed in both populations (Fig. 6C; Supplemental Fig. S6; Supplemental Tables S26, S27; Copenhaver et al. 1999; Yelina et al. 2015).

**Table 1.**
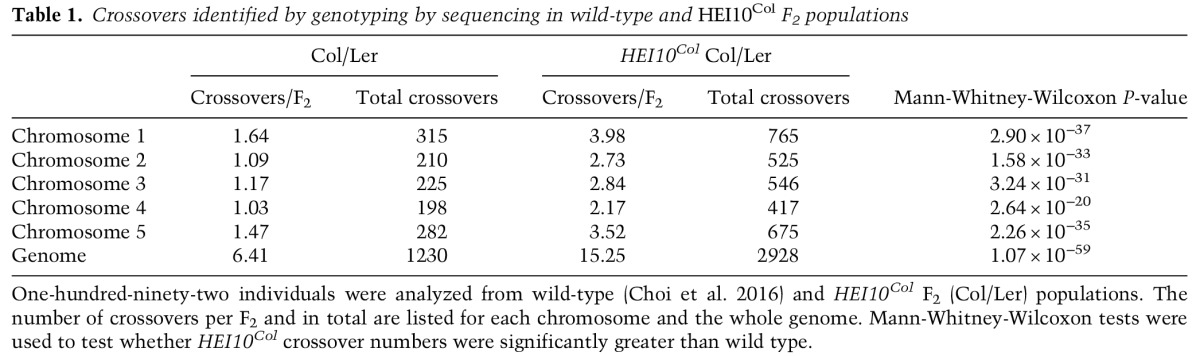
Crossovers identified by genotyping by sequencing in wild-type and *HEI10*^*Col*^ F_2_ populations

Crossovers were mapped using ∼1×–2× depth sequencing data and the TIGER analysis pipeline (Supplemental Table S25; Rowan et al. 2015), which resolved events to a mean width of 976 base pairs (bp). To analyze the fine-scale distribution of wild type versus *HEI10* crossovers, we overlapped them with gene and transposon annotations and compared them with matched sets of randomly chosen intervals (Supplemental Fig. S6A; Supplemental Table S28). Both wild-type and *HEI10* crossovers show increased intergenic and decreased transposon overlap compared with random (Supplemental Fig. S6A; Supplemental Table S28), which is consistent with *Arabidopsis* crossover hot spots associating with euchromatic gene promoters and terminators (Choi et al. 2013; Mercier et al. 2015). We also compared DNA methylation levels and observed that crossovers from both populations were hypomethylated in CG, CHG, and CHH sequence contexts compared with random (Supplemental Fig. S6B; Stroud et al. 2013). This is further consistent with both wild-type and *HEI10* crossovers being enriched within euchromatic regions along the chromosome arms.

## Discussion

Plants, fungi, and invertebrates possess a single *HEI10/RNF212* ortholog (Bhalla et al. 2008; Chelysheva et al. 2012; Wang et al. 2012; Serrentino et al. 2013; De Muyt et al. 2014; Lake et al. 2015), whereas vertebrates encode separate RNF212 and HEI10 proteins that function as antagonistic SUMO and ubiquitin E3 ligases (Ward et al. 2007; Reynolds et al. 2013; Qiao et al. 2014; Gray and Cohen 2016; Rao et al. 2017). In mice, HEI10 and RNF212 promote stable accumulation of MSH4/MSH5 (MutSγ) heterodimers on recombining meiotic chromosomes via a SUMO–ubiquitin relay, which promotes recruitment of MLH1/MLH3 (MutLγ) and crossover formation (Ward et al. 2007; Reynolds et al. 2013; Qiao et al. 2014; Gray and Cohen 2016; Rao et al. 2017). However, it remains unclear which proteins are direct ubiquitin/SUMO targets of the HEI10/RNF212 E3 ligases, although rice HEI10 can directly interact with MSH5 (Wang et al. 2012). HEI10/RNF212 RING domains are C_3_HC_4_ zinc fingers, which generally function as protein interaction domains to recruit E2 ubiquitin-conjugating enzymes to substrates (Deshaies and Joazeiro 2009). Regions outside the RING domain are known to contribute to substrate recognition (Deshaies and Joazeiro 2009). Therefore, we postulate that the HEI10 C-terminal R264 variant may alter substrate recognition efficiency and SUMO/ubiquitin transfer during regulation of meiotic recombination.

Beyond genetic variation that alters HEI10 function, we demonstrated that *Arabidopsis* crossover frequency is exquisitely sensitive to *HEI10* dosage. We propose that higher HEI10 concentration at meiotic repair foci quantitatively promotes crossovers via increased SUMO or ubiquitin transfer to substrate recombination factors. The dosage sensitivity of *Arabidopsis HEI10* is strikingly reminiscent of *rnf212* and *hei10* mutations in mice, which show haploinsufficiency (Reynolds et al. 2013; Qiao et al. 2014). Furthermore, polymorphisms in *RNF212* and *HEI10* genes have been associated with variation in recombination rate in human, cattle, and sheep populations (Kong et al. 2008; Fledel-Alon et al. 2011; Sandor et al. 2012; Johnston et al. 2016). We propose that haploinsufficiency and dosage sensitivity of *HEI10/RNF212* genes predisposes them to acting as *trans* recombination modifiers in diverse eukaryotic lineages. It is interesting to note that increased *HEI10* dosage in *Arabidopsis* led to the greatest crossover increase in subtelomeric euchromatin, which is similar to the sex differences in recombination observed in both plants and mammals (Coop and Przeworski 2006; Giraut et al. 2011). For example, *Arabidopsis* male meiosis shows subtelomeric increases in crossover frequency (Giraut et al. 2011). Therefore, we speculate that differences in HEI10/RNF212 expression or regulation have the potential to contribute to sex differences in recombination. We also note that increasing *HEI10* copy number may be an attractive mechanism to elevate crossover numbers during breeding of crop species.

Crossover modifier loci are able to alter population responses to selection (Feldman et al. 1996). For example, recombination can mitigate the effects of Hill-Robertson interference when linked loci are under selection (Hill and Robertson 1966; Barton and Charlesworth 1998). Therefore, loci that modify crossover frequency may influence genetic adaptation to diverse environments and conditions. Interestingly, total recombination levels compared across eukaryotes are generally low, typically with one or two crossovers per chromosome per meiosis, despite wide variation in physical genome size (Mercier et al. 2015). It is possible that high recombination levels might cause infertility and be selected against. However, *Arabidopsis* anti-crossover pathway mutants show normal fertility despite greatly elevated crossover frequency, at least in the short term (Girard et al. 2015; Mercier et al. 2015). Therefore, we propose that *Arabidopsis* recombination modifiers may act to maintain relatively low crossover numbers. As *rQTL1*^*Col*^ and *rQTL4*^*Col*^ alleles show opposite effects on crossover frequency, this example is consistent with antagonistic modifiers acting to balance recombination. It is also important to note that the effect of modifiers will be highly dependent on genome architecture and outcrossing levels. Crossover modifiers may be especially common in plants, where frequent polyploidization causes challenges for balanced meiotic genome transmission (Bomblies et al. 2016). Indeed, meiotic axis proteins (*ASY1*, *ASY3*, *PDS5*, *ZYP1a*, *ZYP1b*, *SMC1*, and *REC8*) have been strongly selected during polyploid evolution in *Arabidopsis arenosa* (Yant et al. 2013), and the *Ph1* locus is required for promotion of homologous versus homeologous recombination in hexaploid bread wheat (Martín et al. 2014). Therefore, further study of plant meiotic modifier loci is likely to reveal insights into the control of recombination and how this interacts with selection during evolution.

## Materials and methods

### *Arabidopsis* strains

Crossover frequency was measured using fluorescent reporters in seeds (Col-*420*) and pollen (Col-*I2f*) (Emmanuel et al. 2006; Berchowitz and Copenhaver 2008; Yelina et al. 2013; Ziolkowski et al. 2015). In F_2_ populations derived from FTL hemizygotes, only a subset of progeny will contain the fluorescent protein-encoding transgenes also in a hemizygous state, which is necessary for crossover measurement. When using the seed-based *420* line, it is possible to enrich for FTL hemizygous F_2_ plants by examining seed under a fluorescence microscope prior to sowing and separating nonfluorescent, hemizygous, and homozygous seeds based on eGFP and dsRed fluorescence intensities. CSLs were kindly provided by Erik Wijnker, Jose van der Belt, and Joost Keurentjes (University of Wageningen) (Wijnker et al. 2012), with the exception of LCCCC, which was obtained from an *esd7-1* backcross line (del Olmo et al. 2010). Mt-0, Ct-1, and Cvi-0 accessions were obtained from the Nottingham *Arabidopsis* Stock Centre. The ZMM mutant alleles used were *hei10-2* (Salk_014624), *msh4-1* (Salk_136296), *msh5-1* (Salk_110240), *ptd1* (Salk_ 127447), and *shoc1-1* (Salk_057589). Genotyping primer sequences for these mutations used with the LBb1.3 T-DNA left border primer are in Supplemental Table S29.

### rQTL mapping

Genomic DNA was extracted using CTAB and genotyped using PCR amplification of Col/Ler SSLP, CAPS, or dCAPS markers (Supplemental Tables S30, S31). We performed one- and two-dimensional QTL mapping using the R statistical package rQTL. We implemented the Haley-Knott regression algorithm using 2.5-cM steps across the genome and 0.1-cM steps for *rQTL1* fine mapping. To fit models with multiple QTLs, we used the *fitqtl* function with Haley-Knott regression. We used 10,000 permutations for each mapping population to empirically calculate genome-wide LOD score significance thresholds.

### *HEI10* transformation

*HEI10* was amplified from Col or Ler genomic DNA using primers *HEI10-XbaI* and *HEI10-BamHI* (Supplemental Table S29). Amplification products were cloned into the pGREEN-0029 binary vector using XbaI and BamHI restriction enzymes. Promoter swap constructs were generated using XbaI/PacI digestion and vector religation. These vectors were transformed into Col-*420* FTL hemizygous plants using *Agrobacterium* strain GV3101 and floral dipping.

### Quantitative gene expression analysis

RNA was extracted from ∼40 mg of immature flower buds (closed buds up to stage 12, which contain all meiotic stages) using TRI reagent (Sigma-Aldrich). Reverse transcription was performed with SuperScript II reverse transcriptase (ThermoFisher Scientific). Relative *HEI10* expression was measured by qPCR using primers *HEI10-qPCR1* and *HEI10-qPCR2*, and the meiosis-specific gene *DMC1* was amplified using primers *DMC1-qPCR1* and *DMC1-qPCR2* as a control for ΔCt calculations (Supplemental Table S29). For *HEI10* T_1_ analysis, the 2^−ΔΔCt^ method was used to quantify relative transcript levels in comparison with untransformed plants.

### Meiotic cytology and immunostaining

Chromosome spreads of *Arabidopsis* pollen mother cells and rDNA in situ hybridization were performed as described using fixed buds (Sanchez-Moran et al. 2007). Pachytene stage meiocytes were immunostained for ASY1, ZYP1, MLH1, and HEI10 using fresh buds as described (Armstrong et al. 2002; Sanchez-Moran et al. 2007) with the following antibodies: α-ASY1 (rabbit; 1:500 dilution), α-MLH1 (rabbit, IgG-purified; 1:200 dilution), α-ZYP1 (rat; 1:500 dilution), and α-HEI10 (rabbit; 1:200 dilution) (Armstrong et al. 2002; Sanchez-Moran et al. 2007; Chelysheva et al. 2012; Lambing et al. 2015). HEI10-immunostained slides within experiments were prepared side by side, and images were captured using the same exposure times. HEI10-immunostained cell images were acquired as *Z*-stacks of 10-µm × 0.4-µm optical sections, and maximum intensity projections were reconstructed using ImageJ (Lambing et al. 2015). Cell boundaries were defined manually, and total signal intensity within cells was measured. An adjacent image region was used to measure background intensity, and this value was subtracted from the cell intensity. The fluorescence signal from an adjacent Inspeck Red microsphere (ThermoFisher Scientific) was also used to normalize HEI10 signal intensity. Microscopy was conducted using a DeltaVision personal DV microscope (Applied Precision/GE Healthcare) equipped with a CDD Coolsnap HQ2 camera (Photometrics). Image capture was performed using SoftWoRx software version 5.5 (Applied precision/GE Healthcare). For MLH1 and ZYP1 coimmunostaining of pachytene nuclei, individual cell images were acquired as *Z*-stacks of 0.1-µm optical sections, and the maximum intensity projection for each cell was rendered using ImageJ. Numbers of MLH1 foci associated with the synaptonemal complex were scored. DAPI staining of chromosomes from metaphase I nuclei and chiasma counting were performed as described (Sanchez-Moran et al. 2002). Image capture was conducted using a Nikon 90i fluorescence microscope. Images were analyzed with NIS-Elements-F software and ImageJ.

### Mapping crossovers via genotyping by sequencing

DNA was extracted using CTAB and used to generate sequencing libraries as described (Rowan et al. 2015; Yelina et al. 2015) with the following modifications. DNA was extracted from three rosette leaves of 5-wk-old plants, and 150 ng of DNA was used as input for each library. DNA was sheared for 20 min at 37°C with 0.4 U of DNA Shearase (Zymo research). Each set of 96 libraries was sequenced on one lane of an Illumina NextSeq500 instrument (300-cycle Mid Output run). FastQ sequencing data files are available from ArrayExpress accessions E-MTAB-4657 (wild type) (Choi et al. 2016) and E-MTAB-4967 (*HEI10*). Sequencing data were analyzed to identify crossovers as reported previously using the TIGER pipeline (Rowan et al. 2015; Yelina et al. 2015; Choi et al. 2016). Crossovers were tallied in 10-kb windows and plotted along chromosomes after smoothing using the R function “filter.” Crossovers were counted and compared between populations using 2 × 2 contingency tables and χ^2^ tests.

## Supplementary Material

Supplemental Material
