# Supplementary material for: Natural variation and dosage of the HEI10 meiotic E3 ligase control Arabidopsis crossover recombination
Source: Genes Dev. 2017 Feb 1;31(3):306–17. doi: 10.1101/gad.295501.116 (PMC5358726; doi:10.1101/gad.295501.116)
Supplement: Supplemental Material [file supp_31_3_306__index.html]

Natural variation and dosage of the HEI10 meiotic E3 ligase control Arabidopsis crossover recombination — Supplemental Material 

# Natural variation and dosage of the HEI10 meiotic E3 ligase control *Arabidopsis* crossover recombination

## Supplemental Material

- Supplemental\_Material\_and\_Legends.docx
- Supplemental\_FigS1.ai
- Supplemental\_FigS4.ai
- Supplemental\_FigS2.docx
- Supplemental\_FigS5.ai
- Supplemental\_FigS3.ai
- Supplemental\_FigS6.ai
