# Supplementary material for: Natural variation and dosage of the HEI10 meiotic E3 ligase control Arabidopsis crossover recombination
Source: Genes Dev. 2017 Feb 1;31(3):306–17. doi: 10.1101/gad.295501.116 (PMC5358726; doi:10.1101/gad.295501.116)
Supplement: Supplemental Material [file supp_gad.295501.116_Supplemental_Material_and_Legends.docx]

Natural variation and dosage of the *HEI10* meiotic E3 ligase control Arabidopsis crossover recombination

Piotr A. Ziolkowski^1,2^†, Charles J. Underwood^1,3^†, Christophe Lambing^1^, Marina Martinez-Garcia^4^, Emma J. Lawrence^1^, Liliana Ziolkowska^1^, Catherine Griffin^1^, Kyuha Choi^1^, F. Chris H. Franklin^4^, Robert A. Martienssen^3^ and Ian R. Henderson^1*^

**Author Contributions:**

PAZ CJU CL MMG EJL KC FCHF RAM and IRH conceived and designed experiments. PAZ CJU CL MMG EJL LZ performed experiments. PAZ CJU CL MMG EJL FCHF RAM and IRH analyzed the data. PAZ CJU CL MMG EJL FCHF RAM and IRH wrote the manuscript.

**Supplemental Figure Legends**

**Supplemental Figure S1. Immunocytological analysis of meiocytes with varying *HEI10* genotypes. (A)** Representative images of pachytene stage male meiocytes stained for the synaptonemal complex protein ZYP1 (green), MLH1 (red) and DAPI (DNA, blue) from plants with the 4 possible *rQTL* genotypes. Scale bars indicate 5 μM. **(B)** As for (A), but analysing chiasmata at metaphase-I. Chromosomes were stained with DAPI and using FISH *5S* (red) and *45S* rDNA (green) probes. Scale bars indicate 5 μM. **(C)** Quantification of MLH1 foci and chiasmata numbers in nuclei derived from plants with the indicated *rQTL* genotypes. **(D)** As for (C), but quantification of chiasmata numbers. **(E)** Representative images showing immunostained leptotene-stage male meiocytes from the indicated genotypes immunostained for ASY1 (green) and HEI10 (red) and counterstained with DAPI (blue). Scale bars indicate 10 μM.

**Supplemental Figure S2. Multiple sequence alignment of plant HEI10 orthologs.** A multiple-sequence alignment generated using higher plant HEI10 orthologues, identified by BLAST. Color-codes are from Clustal Omega, signifying small & hydrophobic (red), acidic (blue), basic (magenta) or hydroxyl/sulfhydryl/amine/glycine (green). The RING domain, coiled-coil domain and C-terminal regions are highlighted beneath the alignments. For *Arabidopsis thaliana* the Col and Ler amino acid variants are shown separately.

**Supplemental Figure S3. Phylogenetic analysis of HEI10-related sequences. (A)** Phylogenetic tree constructed using eukaryotic HEI10/RNF212 family RING domains. Species names are color-coded according to fungal (purple), plant (green), metazoan HEI10 (red) or metazoan RNF212 (blue) clades. The tree is rooted using fungal SNT2 RING domain sequences. **(B)** Structure of selected HEI10/RNF212 family members with the RING (red) and coiled-coil (blue) domains highlighted as colored boxes.

**Supplemental Figure S4. Geographic distribution of the non-synonymous *HEI10* R264G polymorphism. (A)** Geographical map showing the distribution of Arabidopsis accessions carrying the likely ancestral G264 allele (red, e.g. Ler), or the derived R264 variant (blue, e.g. Col) allele. Data is from the 1,001 Genomes Project (Alonso-Blanco et al. 2016). **(B)** As for (A), but showing a wider geographical area.

**Supplemental Figure S5. Crossovers identified by genotyping-by-sequencing in wild type and *HEI10^Col^* F_2_ populations. (A)** Histograms showing the number of crossover events mapped by genotyping-by-sequencing in the wild type Col/Ler F_2_ population (Choi et al. 2016), for the whole genome and by individual chromosome (Table 1). Mean values are indicated by the vertical red dotted lines. **(B)** As for (A), but showing data from the *HEI10^Col^* F_2_ population.

**Supplemental Figure S6. Chromosomal distribution and DNA methylation of crossover locations in wild type and *HEI10^Col^* F_2_ populations. (A)** Barplots showing overlap (megabases) between crossover intervals, or matched randomly chosen intervals, with TAIR10 representative genes (green), transposons (red), or other sequences (blue) in wild type and *HEI10* F_2_ populations. Analysis was performed separately for chromosome arms, pericentromeres or centromeres (Supplemental Table S26). **(B)** The proportion of Col DNA methylation in CG, CHG or CHH contexts (Stroud et al. 2013), within crossovers or randomly chosen intervals in the chromosome arms or pericentromeres for wild type and *HEI10^Col^*.

**Supplemental Tables**

**Supplemental Table S1.** **Col*-420***×**Ler F_2_ fluorescent count data.** Genetic distance is calculated as cM = 100 × (1 – (1 − 2(*N_G_*+*N_R_*)/*N_T_*)^1/2^), where *N_G_* is the number of green alone seeds, *N_R_* is the number of red alone seeds and *N_T_* is the total number of seeds analysed.

| Individual | Green alone | Red alone | Both colours | No colour | Total | cM |
| --- | --- | --- | --- | --- | --- | --- |
| P4 | 196 | 160 | 1989 | 490 | 2835 | 13.46 |
| BD4 | 219 | 196 | 2156 | 563 | 3134 | 14.26 |
| B2 | 189 | 197 | 1918 | 556 | 2860 | 14.56 |
| BJ2 | 244 | 213 | 2312 | 588 | 3357 | 14.69 |
| U1 | 204 | 206 | 2058 | 530 | 2998 | 14.77 |
| P3 | 227 | 185 | 2067 | 470 | 2949 | 15.11 |
| AF5 | 205 | 220 | 2062 | 539 | 3026 | 15.20 |
| CC3 | 210 | 225 | 2061 | 564 | 3060 | 15.40 |
| AF3 | 233 | 225 | 2169 | 493 | 3120 | 15.95 |
| CD3 | 256 | 243 | 2278 | 621 | 3398 | 15.96 |
| CE2 | 220 | 221 | 2047 | 514 | 3002 | 15.96 |
| BI4 | 196 | 220 | 1956 | 455 | 2827 | 15.99 |
| BH4 | 250 | 216 | 2140 | 542 | 3148 | 16.10 |
| F4 | 252 | 214 | 2106 | 561 | 3133 | 16.18 |
| M1 | 193 | 237 | 1918 | 525 | 2873 | 16.29 |
| CD2 | 264 | 218 | 2176 | 554 | 3212 | 16.34 |
| M2 | 201 | 196 | 1787 | 458 | 2642 | 16.37 |
| BA2 | 225 | 215 | 2001 | 482 | 2923 | 16.40 |
| AM5 | 246 | 247 | 2193 | 555 | 3241 | 16.59 |
| A5 | 236 | 217 | 2001 | 504 | 2958 | 16.71 |
| O2 | 232 | 234 | 2077 | 498 | 3041 | 16.72 |
| D1 | 237 | 229 | 2052 | 521 | 3039 | 16.73 |
| AC3 | 224 | 247 | 2069 | 525 | 3065 | 16.77 |
| BF4 | 207 | 235 | 1938 | 491 | 2871 | 16.81 |
| L3 | 209 | 206 | 1789 | 479 | 2683 | 16.89 |
| D2 | 254 | 246 | 2152 | 560 | 3212 | 17.01 |
| Q5 | 249 | 193 | 1898 | 496 | 2836 | 17.04 |
| CH3 | 234 | 266 | 2159 | 548 | 3207 | 17.04 |
| AR5 | 240 | 211 | 1946 | 477 | 2874 | 17.17 |
| CF1 | 259 | 243 | 2158 | 536 | 3196 | 17.18 |
| O1 | 229 | 245 | 2019 | 524 | 3017 | 17.19 |
| CF2 | 222 | 254 | 2040 | 499 | 3015 | 17.28 |
| BE4 | 248 | 214 | 1965 | 499 | 2926 | 17.28 |
| AI2 | 201 | 207 | 1726 | 428 | 2562 | 17.45 |
| M5 | 234 | 230 | 1953 | 474 | 2891 | 17.60 |
| R2 | 199 | 241 | 1871 | 429 | 2740 | 17.61 |
| AN4 | 183 | 169 | 1509 | 330 | 2191 | 17.62 |
| AB2 | 195 | 196 | 1625 | 416 | 2432 | 17.63 |
| G2 | 242 | 208 | 1877 | 466 | 2793 | 17.67 |
| BD3 | 217 | 237 | 1874 | 484 | 2812 | 17.71 |
| AG1 | 232 | 243 | 1936 | 489 | 2900 | 18.00 |
| AQ7 | 196 | 202 | 1632 | 396 | 2426 | 18.03 |
| AV2 | 231 | 240 | 1899 | 500 | 2870 | 18.04 |
| AE3 | 250 | 222 | 1923 | 477 | 2872 | 18.07 |
| I1 | 189 | 201 | 1611 | 366 | 2367 | 18.12 |
| BC2 | 278 | 238 | 2107 | 505 | 3128 | 18.14 |
| M3 | 264 | 221 | 1980 | 466 | 2931 | 18.20 |
| K5 | 255 | 220 | 1914 | 459 | 2848 | 18.36 |
| AO4 | 218 | 205 | 1668 | 434 | 2525 | 18.46 |
| Z5 | 247 | 241 | 1933 | 482 | 2903 | 18.53 |
| N2 | 292 | 209 | 2033 | 439 | 2973 | 18.58 |
| AB4 | 228 | 240 | 1852 | 441 | 2761 | 18.70 |
| CB3 | 255 | 270 | 2068 | 501 | 3094 | 18.72 |
| L4 | 232 | 239 | 1887 | 413 | 2771 | 18.76 |
| CD1 | 260 | 265 | 2030 | 529 | 3084 | 18.79 |
| AM2 | 244 | 286 | 2047 | 520 | 3097 | 18.90 |
| AE2 | 245 | 221 | 1813 | 440 | 2719 | 18.93 |
| CJ3 | 193 | 258 | 1726 | 449 | 2626 | 18.97 |
| AS4 | 208 | 229 | 1687 | 416 | 2540 | 19.01 |
| BI5 | 245 | 243 | 1839 | 507 | 2834 | 19.03 |
| AA4 | 237 | 254 | 1885 | 461 | 2837 | 19.14 |
| C4 | 245 | 260 | 1941 | 467 | 2913 | 19.17 |
| CJ2 | 233 | 296 | 2030 | 485 | 3044 | 19.23 |
| AP5 | 258 | 264 | 2007 | 472 | 3001 | 19.25 |
| AS5 | 240 | 228 | 1802 | 414 | 2684 | 19.30 |
| AO5 | 251 | 287 | 2047 | 488 | 3073 | 19.39 |
| BC1 | 265 | 269 | 2032 | 462 | 3028 | 19.55 |
| M4 | 230 | 276 | 1899 | 449 | 2854 | 19.66 |
| AK4 | 219 | 257 | 1769 | 437 | 2682 | 19.69 |
| AH4 | 258 | 266 | 1955 | 453 | 2932 | 19.84 |
| CE1 | 282 | 257 | 2013 | 457 | 3009 | 19.89 |
| W3 | 267 | 264 | 1982 | 450 | 2963 | 19.90 |
| K3 | 273 | 230 | 1844 | 458 | 2805 | 19.92 |
| G1 | 238 | 256 | 1792 | 455 | 2741 | 20.03 |
| I5 | 180 | 236 | 1517 | 371 | 2304 | 20.07 |
| R3 | 245 | 238 | 1765 | 427 | 2675 | 20.07 |
| AW2 | 286 | 263 | 1982 | 504 | 3035 | 20.11 |
| S2 | 253 | 246 | 1839 | 415 | 2753 | 20.16 |
| AJ1 | 279 | 250 | 1929 | 456 | 2914 | 20.19 |
| I2 | 290 | 261 | 2003 | 478 | 3032 | 20.22 |
| L5 | 249 | 229 | 1690 | 462 | 2630 | 20.22 |
| R4 | 262 | 269 | 1896 | 493 | 2920 | 20.23 |
| CG4 | 280 | 313 | 2141 | 513 | 3247 | 20.33 |
| CF4 | 310 | 288 | 2148 | 522 | 3268 | 20.37 |
| CC1 | 273 | 276 | 1970 | 465 | 2984 | 20.50 |
| AN1 | 239 | 236 | 1707 | 399 | 2581 | 20.51 |
| C3 | 274 | 266 | 1919 | 472 | 2931 | 20.53 |
| AZ2 | 276 | 242 | 1847 | 437 | 2802 | 20.61 |
| O3 | 282 | 309 | 2095 | 495 | 3181 | 20.73 |
| C1 | 291 | 311 | 2142 | 491 | 3235 | 20.76 |
| AO3 | 292 | 289 | 2031 | 509 | 3121 | 20.77 |
| F5 | 265 | 294 | 1993 | 446 | 2998 | 20.81 |
| B5 | 291 | 307 | 2070 | 531 | 3199 | 20.87 |
| AL1 | 297 | 276 | 2001 | 482 | 3056 | 20.94 |
| AD5 | 275 | 279 | 1930 | 470 | 2954 | 20.95 |
| CH1 | 284 | 270 | 1924 | 458 | 2936 | 21.09 |
| H4 | 276 | 284 | 1951 | 454 | 2965 | 21.12 |
| AL3 | 300 | 241 | 1874 | 425 | 2840 | 21.32 |
| V2 | 256 | 223 | 1614 | 392 | 2485 | 21.61 |
| AF1 | 253 | 282 | 1791 | 445 | 2771 | 21.65 |
| U3 | 289 | 249 | 1830 | 408 | 2776 | 21.74 |
| AA1 | 280 | 278 | 1907 | 408 | 2873 | 21.80 |
| BH1 | 280 | 292 | 1908 | 450 | 2930 | 21.93 |
| CD4 | 277 | 266 | 1814 | 421 | 2778 | 21.96 |
| T4 | 256 | 283 | 1762 | 452 | 2753 | 22.00 |
| AF2 | 301 | 320 | 2051 | 499 | 3171 | 22.00 |
| BG5 | 311 | 280 | 1957 | 450 | 2998 | 22.17 |
| AE1 | 305 | 254 | 1868 | 402 | 2829 | 22.23 |
| U5 | 232 | 274 | 1670 | 380 | 2556 | 22.28 |
| T5 | 299 | 271 | 1886 | 422 | 2878 | 22.29 |
| O5 | 283 | 285 | 1872 | 418 | 2858 | 22.38 |
| X3 | 294 | 283 | 1898 | 410 | 2885 | 22.54 |
| Z2 | 186 | 201 | 1255 | 316 | 1958 | 22.54 |
| CK1 | 301 | 286 | 1905 | 428 | 2920 | 22.67 |
| F1 | 325 | 298 | 1999 | 453 | 3075 | 22.88 |
| AM1 | 320 | 269 | 1885 | 431 | 2905 | 22.90 |
| AE5 | 305 | 286 | 1869 | 448 | 2908 | 22.96 |
| X4 | 302 | 312 | 1917 | 467 | 2998 | 23.16 |
| CH2 | 337 | 329 | 2108 | 475 | 3249 | 23.19 |
| CA4 | 329 | 319 | 2062 | 444 | 3154 | 23.25 |
| BI2 | 296 | 320 | 1996 | 382 | 2994 | 23.29 |
| AV4 | 309 | 236 | 1600 | 495 | 2640 | 23.38 |
| CI1 | 332 | 321 | 2041 | 454 | 3148 | 23.51 |
| E3 | 323 | 338 | 2036 | 477 | 3174 | 23.61 |
| Q1 | 287 | 302 | 1797 | 433 | 2819 | 23.70 |
| AX1 | 306 | 282 | 1824 | 400 | 2812 | 23.72 |
| AN2 | 309 | 322 | 1927 | 455 | 3013 | 23.77 |
| CG1 | 336 | 352 | 2089 | 475 | 3252 | 24.05 |
| K4 | 315 | 321 | 1949 | 419 | 3004 | 24.07 |
| AY3 | 302 | 248 | 1634 | 381 | 2565 | 24.43 |
| AH5 | 286 | 222 | 1501 | 338 | 2347 | 24.69 |
| AW4 | 347 | 333 | 2015 | 437 | 3132 | 24.78 |
| CG2 | 276 | 356 | 1803 | 459 | 2894 | 24.95 |
| Q3 | 241 | 361 | 1711 | 419 | 2732 | 25.21 |
| AZ1 | 285 | 254 | 1530 | 361 | 2430 | 25.41 |
| BG2 | 363 | 332 | 2000 | 421 | 3116 | 25.57 |
| V1 | 339 | 338 | 1908 | 389 | 2974 | 26.19 |
| AI3 | 335 | 309 | 1745 | 390 | 2779 | 26.75 |
| CK2 | 368 | 363 | 2013 | 408 | 3152 | 26.78 |
| AJ2 | 362 | 335 | 1875 | 378 | 2950 | 27.37 |
| CA3 | 404 | 379 | 2020 | 417 | 3220 | 28.33 |
| AP2 | 323 | 319 | 1625 | 333 | 2600 | 28.86 |
| AH2 | 358 | 399 | 1902 | 402 | 3061 | 28.91 |
| CJ1 | 408 | 449 | 1963 | 347 | 3167 | 32.27 |
| AQ3 | 323 | 313 | 1354 | 254 | 2244 | 34.19 |

**Supplemental Table S2. Col*-420*/Col fluorescent count data.** Genetic distance is calculated as cM = 100 × (1 – (1 − 2(*N_G_*+*N_R_*)/*N_T_*)^1/2^), where *N_G_* is the number of green alone seeds, *N_R_* is the number of red alone seeds and *N_T_* is the total number of seeds analysed.

| Individual | Green alone | Red alone | Both colours | No colour | Total | cM |
| --- | --- | --- | --- | --- | --- | --- |
| wt-1 | 169 | 189 | 1718 | 457 | 2533 | 15.30 |
| wt-2 | 273 | 307 | 2227 | 580 | 3387 | 18.91 |
| wt-3 | 279 | 235 | 2046 | 563 | 3123 | 18.10 |
| wt-4 | 241 | 259 | 2081 | 514 | 3095 | 17.73 |
| wt-5 | 235 | 283 | 1892 | 420 | 2830 | 20.38 |
| wt-6 | 302 | 213 | 1826 | 503 | 2844 | 20.14 |
| wt-7 | 268 | 177 | 1865 | 474 | 2784 | 17.52 |
| wt-8 | 278 | 224 | 1971 | 506 | 2979 | 18.58 |
| wt-9 | 212 | 163 | 1463 | 404 | 2242 | 18.42 |
| wt-10 | 263 | 292 | 2339 | 569 | 3463 | 17.57 |
| wt-11 | 407 | 242 | 2184 | 646 | 3479 | 20.82 |
| wt-12 | 416 | 286 | 2421 | 706 | 3829 | 20.42 |
| wt-13 | 375 | 239 | 2095 | 549 | 3258 | 21.06 |
| wt-14 | 346 | 189 | 1983 | 479 | 2997 | 19.81 |
| wt-15 | 321 | 281 | 2435 | 628 | 3665 | 18.06 |
| wt-16 | 315 | 240 | 1907 | 458 | 2920 | 21.27 |
| wt-17 | 237 | 190 | 1759 | 535 | 2721 | 17.17 |
| wt-18 | 150 | 135 | 1091 | 270 | 1646 | 19.15 |
| wt-19 | 295 | 257 | 2286 | 567 | 3405 | 17.79 |
| wt-20 | 325 | 313 | 2327 | 588 | 3553 | 19.95 |
| wt-21 | 224 | 295 | 2041 | 499 | 3059 | 18.72 |
| wt-22 | 273 | 281 | 2273 | 535 | 3362 | 18.12 |
| wt-23 | 165 | 202 | 1203 | 279 | 1849 | 22.35 |
| wt-24 | 159 | 218 | 1798 | 458 | 2633 | 15.52 |
| wt-25 | 239 | 266 | 1943 | 508 | 2956 | 18.86 |
| wt-26 | 294 | 267 | 2304 | 597 | 3462 | 17.79 |
| wt-27 | 331 | 339 | 2280 | 583 | 3533 | 21.21 |
| wt-28 | 329 | 277 | 2230 | 577 | 3413 | 19.70 |
| wt-29 | 159 | 143 | 1284 | 344 | 1930 | 17.11 |
| wt-30 | 246 | 214 | 2017 | 477 | 2954 | 17.02 |
| wt-31 | 210 | 242 | 1636 | 424 | 2512 | 19.99 |
| wt-32 | 254 | 210 | 1937 | 489 | 2890 | 17.61 |
| wt-33 | 261 | 226 | 1864 | 460 | 2811 | 19.16 |
| wt-34 | 142 | 198 | 1367 | 405 | 2112 | 17.66 |
| wt-35 | 276 | 272 | 2152 | 554 | 3254 | 18.56 |
| wt-36 | 337 | 362 | 2500 | 630 | 3829 | 20.32 |
| wt-37 | 221 | 204 | 1609 | 346 | 2380 | 19.82 |
| wt-38 | 245 | 226 | 1606 | 405 | 2482 | 21.23 |
| wt-39 | 226 | 186 | 1689 | 448 | 2549 | 17.74 |
| wt-40 | 363 | 250 | 2138 | 540 | 3291 | 20.79 |
| wt-41 | 139 | 118 | 1011 | 297 | 1565 | 18.05 |
| wt-42 | 196 | 213 | 1960 | 507 | 2876 | 15.41 |
| wt-43 | 372 | 318 | 2314 | 560 | 3564 | 21.72 |
| wt-44 | 381 | 309 | 2290 | 468 | 3448 | 22.56 |
| wt-45 | 372 | 331 | 2397 | 544 | 3644 | 21.63 |
| wt-46 | 338 | 316 | 2305 | 564 | 3523 | 20.71 |

**Supplemental Table S3.** **Col*-420***×**CSL F_1_ fluorescent count data.** The genotype column lists the CSL genotype of plants that were crossed to Col*-420*, where C and L denote Col and Ler genotypes respectively, for the 5 Arabidopsis chromosomes. Data presented is from replicate F_1_ individuals derived from these crosses. Genetic distance is calculated as cM = 100 × (1 – (1 − 2(*N_G_*+*N_R_*)/*N_T_*)^1/2^), where *N_G_* is the number of green alone seeds, *N_R_* is the number of red alone seeds and *N_T_* is the total number of seeds analysed. *X^2^* tests were performed by comparing the sum of green and red alone counts versus the sum of both colour and no colour counts between CCCCC and the indicated CSL genotype, using 2×2 contingency tables.

| Genotype | Green alone | Red alone | Both | None | Total | cM | *X^2^ P* |
| --- | --- | --- | --- | --- | --- | --- | --- |
| CCCCC | 244 | 238 | 1751 | 427 | 2660 | 20.15 |  |
| CCCCC | 261 | 223 | 1865 | 502 | 2851 | 18.73 |  |
| CCCCC | 214 | 233 | 1687 | 455 | 2589 | 19.09 |  |
| CCCCC | 210 | 237 | 1716 | 441 | 2604 | 18.96 | n.d. |
| CCCCL | 211 | 237 | 1663 | 507 | 2618 | 18.9 |  |
| CCCCL | 211 | 247 | 1732 | 459 | 2649 | 19.12 |  |
| CCCCL | 239 | 236 | 1687 | 508 | 2670 | 19.74 |  |
| CCCCL | 192 | 252 | 1575 | 637 | 2656 | 18.41 | 0.789 |
| CCCLC | 243 | 279 | 1584 | 451 | 2557 | 23.08 |  |
| CCCLC | 253 | 247 | 1788 | 460 | 2748 | 20.24 |  |
| CCCLC | 246 | 270 | 1777 | 462 | 2755 | 20.92 |  |
| CCCLC | 247 | 291 | 1751 | 434 | 2723 | 22.23 |  |
| CCCLC | 223 | 253 | 1727 | 435 | 2638 | 20.05 | 1.14×10^-3^ |
| CCCLL | 219 | 247 | 1699 | 449 | 2614 | 19.78 |  |
| CCCLL | 221 | 237 | 1762 | 453 | 2673 | 18.93 |  |
| CCCLL | 204 | 239 | 1709 | 432 | 2584 | 18.94 |  |
| CCCLL | 227 | 240 | 1627 | 454 | 2548 | 20.41 |  |
| CCCLL | 227 | 234 | 1748 | 464 | 2673 | 19.06 | 0.770 |
| CCLCC | 268 | 239 | 2001 | 508 | 3016 | 18.53 |  |
| CCLCC | 246 | 267 | 1748 | 557 | 2818 | 20.26 |  |
| CCLCC | 276 | 298 | 1864 | 486 | 2924 | 22.06 |  |
| CCLCC | 282 | 287 | 1636 | 489 | 2694 | 24 |  |
| CCLCC | 255 | 256 | 1723 | 535 | 2769 | 20.57 | 4.06×10^-3^ |
| CLCCL | 248 | 266 | 1781 | 551 | 2846 | 20.08 |  |
| CLCCL | 230 | 239 | 1696 | 515 | 2680 | 19.38 |  |
| CLCCL | 233 | 248 | 1684 | 532 | 2697 | 19.79 |  |
| CLCCL | 200 | 277 | 1604 | 524 | 2605 | 20.39 | 0.299 |
| CLCLC | 277 | 265 | 1788 | 404 | 2734 | 22.31 |  |
| CLCLC | 258 | 273 | 1686 | 371 | 2588 | 23.21 |  |
| CLCLC | 253 | 288 | 1779 | 390 | 2710 | 22.49 |  |
| CLCLC | 228 | 290 | 1662 | 433 | 2613 | 22.31 |  |
| CLCLC | 261 | 263 | 1711 | 434 | 2669 | 22.07 | 4.32×10^-7^ |
| CLCLL | 276 | 231 | 1817 | 505 | 2829 | 19.9 |  |
| CLCLL | 299 | 290 | 1756 | 504 | 2849 | 23.42 |  |
| CLCLL | 249 | 254 | 1861 | 477 | 2841 | 19.63 |  |
| CLCLL | 259 | 267 | 1853 | 474 | 2853 | 20.55 | 0.012 |
| CLLLL | 246 | 241 | 1874 | 419 | 2780 | 19.4 |  |
| CLLLL | 269 | 251 | 1797 | 418 | 2735 | 21.28 |  |
| CLLLL | 253 | 247 | 1794 | 391 | 2685 | 20.78 |  |
| CLLLL | 261 | 256 | 1973 | 435 | 2925 | 19.6 |  |
| CLLLL | 221 | 257 | 1846 | 422 | 2746 | 19.26 | 0.184 |
| LCCCC | 214 | 201 | 1917 | 524 | 2856 | 15.78 |  |
| LCCCC | 215 | 228 | 1873 | 507 | 2823 | 17.17 |  |
| LCCCC | 204 | 205 | 1884 | 516 | 2809 | 15.81 |  |
| LCCCC | 174 | 214 | 1840 | 456 | 2684 | 15.69 |  |
| LCCCC | 208 | 200 | 1895 | 520 | 2823 | 15.68 |  |
| LCCCC | 169 | 216 | 1844 | 489 | 2718 | 15.34 |  |
| LCCCC | 189 | 209 | 1871 | 455 | 2724 | 15.87 |  |
| LCCCC | 178 | 188 | 1919 | 499 | 2784 | 14.15 |  |
| LCCCC | 186 | 222 | 1943 | 440 | 2791 | 15.88 | 3.42×10^-12^ |
| LCCLC | 231 | 212 | 1685 | 454 | 2582 | 18.95 |  |
| LCCLC | 232 | 200 | 1877 | 467 | 2776 | 17.01 |  |
| LCCLC | 211 | 197 | 1747 | 471 | 2626 | 16.98 |  |
| LCCLC | 230 | 211 | 1789 | 490 | 2720 | 17.8 |  |
| LCCLC | 190 | 240 | 1766 | 495 | 2691 | 17.51 | 7.68×10^-3^ |
| LCCLL | 198 | 196 | 1914 | 505 | 2813 | 15.15 |  |
| LCCLL | 199 | 198 | 1850 | 462 | 2709 | 15.92 |  |
| LCCLL | 173 | 198 | 1877 | 481 | 2729 | 14.67 |  |
| LCCLL | 205 | 204 | 1899 | 484 | 2792 | 15.92 |  |
| LCCLL | 220 | 213 | 1857 | 458 | 2748 | 17.24 | 1.52×10^-9^ |
| LLCLL | 203 | 197 | 1988 | 517 | 2905 | 14.88 |  |
| LLCLL | 210 | 233 | 1986 | 522 | 2951 | 16.35 |  |
| LLCLL | 219 | 242 | 1920 | 544 | 2925 | 17.25 |  |
| LLCLL | 224 | 213 | 1843 | 469 | 2749 | 17.41 |  |
| LLCLL | 211 | 239 | 1941 | 541 | 2932 | 16.75 | 2.20×10^-6^ |
| LLLLL | 171 | 194 | 1770 | 439 | 2574 | 15.36 |  |
| LLLLL | 169 | 179 | 1766 | 518 | 2632 | 14.24 |  |
| LLLLL | 175 | 200 | 1880 | 497 | 2752 | 14.71 |  |
| LLLLL | 190 | 186 | 1848 | 494 | 2718 | 14.95 |  |
| LLLLL | 201 | 186 | 1878 | 529 | 2794 | 14.97 | 7.86×10^-15^ |

**Supplemental Table S4. Col-*I2f***×**CSL F_1_ fluorescent count data.** The genotype column lists the CSL genotype of plants that were crossed to Col-*I2f*, where C and L denote Col and Ler genotypes respectively, for the 5 Arabidopsis chromosomes. These data are from replicate F_1_ plants. cM was calculated as G/(G+Both), where ‘G’ denotes the number of pollen that were green alone (Green) and ‘Both’ denotes red and green colours. The number of recombinant (Green) and non-recombinant (Both) counts for CCCCC and CSL genotypes were used to construct 2×2 contingency tables and *X^2^* tests performed to test for significant differences. At least three biological replicates were analysed per genotype, with pollen extracted from 5-9 pooled individuals per replicate.

| Genotype | Red alone | Both | None | Green alone | cM | *X^2^ P* |
| --- | --- | --- | --- | --- | --- | --- |
| CCCCC | 2151 | 11657 | 14849 | 1063 | 8.36 |  |
| CCCCC | 1124 | 7048 | 7439 | 658 | 8.54 |  |
| CCCCC | 2226 | 14308 | 15314 | 1370 | 8.74 | n.d. |
| CCCCL | 3465 | 20313 | 27175 | 2192 | 9.74 |  |
| CCCCL | 4348 | 20238 | 30796 | 2193 | 9.78 |  |
| CCCCL | 4129 | 20923 | 30963 | 2188 | 9.47 |  |
| CCCCL | 4400 | 19922 | 27918 | 1953 | 8.93 | 3.49×10^-7^ |
| CCCLC | 4008 | 24983 | 28691 | 2405 | 8.78 |  |
| CCCLC | 3231 | 20406 | 22983 | 1930 | 8.64 |  |
| CCCLC | 3360 | 20611 | 24490 | 2003 | 8.86 |  |
| CCCLC | 3192 | 20098 | 22716 | 1834 | 8.36 | 0.544 |
| CCCLL | 3255 | 20423 | 26271 | 2069 | 9.2 |  |
| CCCLL | 3860 | 19808 | 30791 | 2007 | 9.2 |  |
| CCCLL | 3672 | 19921 | 28097 | 1956 | 8.94 |  |
| CCCLL | 4141 | 17937 | 27646 | 1856 | 9.38 | 6.57×10^-4^ |
| CCLCC | 3465 | 18583 | 24899 | 1999 | 9.71 |  |
| CCLCC | 3734 | 22947 | 29308 | 2470 | 9.72 |  |
| CCLCC | 1984 | 13117 | 15284 | 1368 | 9.44 |  |
| CCLCC | 2322 | 13468 | 16766 | 1414 | 9.5 | 1.23 x 10^-8^ |
| CLCCL | 3807 | 19792 | 28728 | 2193 | 9.97 |  |
| CLCCL | 4314 | 20719 | 28216 | 2355 | 10.21 |  |
| CLCCL | 3860 | 15972 | 27216 | 1570 | 8.95 |  |
| CLCCL | 4075 | 19887 | 29239 | 2350 | 10.57 | 1.65×10^-14^ |
| CLCLC | 4238 | 20517 | 25139 | 2097 | 9.27 |  |
| CLCLC | 4032 | 20037 | 25364 | 2108 | 9.52 |  |
| CLCLC | 3728 | 20904 | 22958 | 1992 | 8.7 |  |
| CLCLC | 4138 | 20775 | 24402 | 2077 | 9.09 | 1.15×10^-3^ |
| CLCLL | 4017 | 21731 | 29251 | 2341 | 9.72 |  |
| CLCLL | 3917 | 20302 | 24847 | 2125 | 9.48 |  |
| CLCLL | 3803 | 20102 | 26500 | 2041 | 9.22 |  |
| CLCLL | 4437 | 23808 | 28422 | 2624 | 9.93 | 6.95×10^-9^ |
| CLLLL | 3773 | 20144 | 22482 | 2170 | 9.72 |  |
| CLLLL | 3648 | 20656 | 24424 | 2538 | 10.94 |  |
| CLLLL | 3628 | 20174 | 24333 | 2318 | 10.31 |  |
| CLLLL | 3716 | 20950 | 24888 | 2476 | 10.57 | 5.73×10^-23^ |
| LCCCC | 2096 | 13954 | 19567 | 891 | 6 |  |
| LCCCC | 2419 | 16158 | 21404 | 1122 | 6.49 |  |
| LCCCC | 2543 | 18495 | 23520 | 1313 | 6.63 | 1.11×10^-33^ |
| LCCLC | 4546 | 29992 | 33028 | 2132 | 6.64 |  |
| LCCLC | 2466 | 17517 | 17691 | 1207 | 6.45 |  |
| LCCLC | 2258 | 17533 | 17643 | 1303 | 6.92 |  |
| LCCLC | 2559 | 17385 | 18273 | 1256 | 6.74 | 2.69×10^-31^ |
| LCCLL | 1994 | 20239 | 20083 | 1570 | 7.2 |  |
| LCCLL | 1905 | 21229 | 20366 | 1577 | 6.91 |  |
| LCCLL | 1868 | 20548 | 18653 | 1357 | 6.19 |  |
| LCCLL | 2185 | 21350 | 21890 | 1394 | 6.13 | 7.20×10^-34^ |
| LCLLL | 2617 | 20052 | 23516 | 1543 | 7.15 |  |
| LCLLL | 3156 | 25694 | 27140 | 1963 | 7.1 |  |
| LCLLL | 2587 | 20513 | 23460 | 1682 | 7.58 |  |
| LCLLL | 2997 | 22158 | 27138 | 1722 | 7.21 | 1.25×10^-15^ |
| LLCLL | 3063 | 20237 | 24617 | 1695 | 7.73 |  |
| LLCLL | 3627 | 23397 | 29967 | 1894 | 7.49 |  |
| LLCLL | 3071 | 21207 | 24021 | 1642 | 7.19 | 3.31×10^-10^ |
| LLLLL | 2522 | 24934 | 23249 | 2052 | 7.6 |  |
| LLLLL | 2218 | 21200 | 22190 | 1747 | 7.61 |  |
| LLLLL | 2548 | 21704 | 20391 | 1749 | 7.46 |  |
| LLLLL | 9123 | 80088 | 85322 | 6762 | 7.79 | 2.13×10^-8^ |

**Supplemental Table S5. Generalized Linear Model analysis of chromosome effects on *420* and *I2f* CSL F_1_ crossover frequency.** The number of crossover and non-crossover counts for replicate measurements were modelled (GLM) as binomial response variables, compared to categorical predictors for each chromosome (Col or Ler). A logistic link function was used.

| *420* | Estimate | Std. Error | z value | *P* |
| --- | --- | --- | --- | --- |
| Intercept | -1.491 | 0.0120 | -124.213 | 0 |
| Chr1 | -0.266 | 0.0128 | -20.682 | 5.05×10^-95^ |
| Chr2 | 0.040 | 0.0153 | 2.643 | 8.22×10^-3^ |
| Chr3 | -0.024 | 0.0154 | -1.587 | 0.113 |
| Chr4 | 0.059 | 0.0142 | 4.138 | 3.50×10^-5^ |
| Chr5 | -0.092 | 0.0141 | -6.499 | 8.06×10^-11^ |
|  |  |  |  |  |
| *I2f* | Estimate | Std. Error | z value | *P* |
| Intercept | -2.327 | 0.0071 | -325.683 | 0 |
| Chr1 | -0.322 | 0.0073 | -43.887 | 0 |
| Chr2 | 0.066 | 0.0071 | 9.180 | 4.32×10^-20^ |
| Chr3 | 0.074 | 0.0071 | 10.354 | 4.00×10^-25^ |
| Chr4 | -0.027 | 0.0078 | -3.463 | 5.34×10^-4^ |
| Chr5 | 0.057 | 0.0073 | 7.786 | 6.93×10^-15^ |

**Supplemental Table S6. Col-*420*×LLCLL F_2_ fluorescent count data.** Genetic distance is calculated as cM = 100 × (1 – (1 − 2(*N_G_*+*N_R_*)/*N_T_*)^1/2^), where *N_G_* is the number of green alone seeds, *N_R_* is the number of red alone seeds and *N_T_* is the total number of seeds analysed.

| Line | Green  colour | Red colour | Both colours | No colour | Total | cM |
| --- | --- | --- | --- | --- | --- | --- |
| 33A | 132 | 151 | 1918 | 519 | 2720 | 11.01 |
| 32D | 142 | 154 | 1936 | 607 | 2839 | 11.04 |
| 52I | 177 | 150 | 2139 | 623 | 3089 | 11.21 |
| 44C | 105 | 118 | 1437 | 429 | 2089 | 11.32 |
| 46A | 176 | 139 | 2031 | 531 | 2877 | 11.62 |
| 56F | 192 | 136 | 2091 | 575 | 2994 | 11.63 |
| 49D | 165 | 160 | 2019 | 600 | 2944 | 11.73 |
| 32H | 144 | 164 | 1932 | 541 | 2781 | 11.77 |
| 50D | 180 | 148 | 2109 | 513 | 2950 | 11.82 |
| 21G | 170 | 190 | 2276 | 571 | 3207 | 11.94 |
| 40C | 186 | 156 | 2130 | 549 | 3021 | 12.05 |
| 39I | 171 | 145 | 1921 | 545 | 2782 | 12.09 |
| 38A | 162 | 155 | 1887 | 559 | 2763 | 12.22 |
| 25D | 165 | 147 | 1855 | 535 | 2702 | 12.30 |
| 40A | 167 | 158 | 1880 | 568 | 2773 | 12.50 |
| 51D | 161 | 171 | 1949 | 549 | 2830 | 12.51 |
| 34H | 172 | 162 | 1982 | 530 | 2846 | 12.52 |
| 1D | 170 | 169 | 1969 | 580 | 2888 | 12.52 |
| 37C | 171 | 155 | 1917 | 534 | 2777 | 12.52 |
| 33G | 171 | 159 | 1956 | 524 | 2810 | 12.53 |
| 3A | 161 | 171 | 1956 | 535 | 2823 | 12.55 |
| 59I | 176 | 167 | 2014 | 555 | 2912 | 12.57 |
| 64B | 163 | 164 | 1923 | 521 | 2771 | 12.59 |
| 26A | 159 | 164 | 1855 | 553 | 2731 | 12.62 |
| 38G | 150 | 120 | 1560 | 450 | 2280 | 12.64 |
| 60A | 178 | 167 | 2009 | 553 | 2907 | 12.67 |
| 32G | 160 | 196 | 2054 | 584 | 2994 | 12.70 |
| 28C | 187 | 177 | 2131 | 561 | 3056 | 12.72 |
| 38H | 177 | 176 | 1998 | 599 | 2950 | 12.78 |
| 23E | 185 | 171 | 2041 | 573 | 2970 | 12.81 |
| 29A | 129 | 100 | 1324 | 355 | 1908 | 12.82 |
| 14C | 176 | 184 | 2053 | 583 | 2996 | 12.84 |
| 58B | 164 | 162 | 1835 | 547 | 2708 | 12.87 |
| 44B | 69 | 53 | 685 | 202 | 1009 | 12.93 |
| 20I | 175 | 169 | 1972 | 516 | 2832 | 12.99 |
| 33D | 174 | 160 | 1884 | 518 | 2736 | 13.06 |
| 1I | 188 | 159 | 1995 | 498 | 2840 | 13.07 |
| 61H | 168 | 164 | 1852 | 532 | 2716 | 13.08 |
| 4H | 149 | 157 | 1711 | 477 | 2494 | 13.13 |
| 28F | 169 | 172 | 1892 | 544 | 2777 | 13.14 |
| 56E | 196 | 187 | 2139 | 595 | 3117 | 13.15 |
| 30A | 168 | 153 | 1794 | 491 | 2606 | 13.19 |
| 6A | 187 | 152 | 1858 | 552 | 2749 | 13.20 |
| 38D | 189 | 169 | 1998 | 542 | 2898 | 13.23 |
| 16F | 193 | 205 | 2206 | 612 | 3216 | 13.25 |
| 13D | 160 | 199 | 1969 | 572 | 2900 | 13.26 |
| 14E | 170 | 180 | 1894 | 583 | 2827 | 13.26 |
| 19B | 194 | 174 | 2025 | 577 | 2970 | 13.27 |
| 53B | 203 | 189 | 2174 | 597 | 3163 | 13.27 |
| 20G | 176 | 162 | 1867 | 518 | 2723 | 13.30 |
| 6G | 171 | 162 | 1851 | 497 | 2681 | 13.31 |
| 40H | 207 | 171 | 2071 | 591 | 3040 | 13.32 |
| 51I | 157 | 209 | 2023 | 550 | 2939 | 13.34 |
| 63I | 179 | 154 | 1821 | 520 | 2674 | 13.34 |
| 18A | 193 | 179 | 2044 | 571 | 2987 | 13.34 |
| 11F | 150 | 162 | 1727 | 465 | 2504 | 13.35 |
| 45B | 183 | 167 | 1928 | 527 | 2805 | 13.37 |
| 8A | 179 | 185 | 2014 | 537 | 2915 | 13.38 |
| 7H | 180 | 159 | 1857 | 510 | 2706 | 13.43 |
| 30H | 181 | 140 | 1738 | 496 | 2555 | 13.47 |
| 57A | 180 | 202 | 2089 | 569 | 3040 | 13.47 |
| 43D | 209 | 153 | 1968 | 549 | 2879 | 13.48 |
| 12E | 186 | 162 | 1927 | 491 | 2766 | 13.49 |
| 51A | 176 | 168 | 1860 | 496 | 2700 | 13.68 |
| 21I | 168 | 200 | 1988 | 527 | 2883 | 13.70 |
| 46I | 174 | 164 | 1802 | 505 | 2645 | 13.72 |
| 57E | 182 | 173 | 1905 | 516 | 2776 | 13.73 |
| 63B | 168 | 161 | 1775 | 465 | 2569 | 13.75 |
| 9B | 187 | 200 | 2087 | 539 | 3013 | 13.80 |
| 24D | 200 | 172 | 1988 | 536 | 2896 | 13.80 |
| 7F | 55 | 42 | 512 | 143 | 752 | 13.86 |
| 37E | 196 | 182 | 2028 | 524 | 2930 | 13.86 |
| 58D | 232 | 190 | 2241 | 606 | 3269 | 13.87 |
| 11B | 173 | 181 | 1891 | 495 | 2740 | 13.88 |
| 36B | 195 | 195 | 2051 | 577 | 3018 | 13.89 |
| 5C | 197 | 171 | 1952 | 527 | 2847 | 13.89 |
| 62E | 176 | 182 | 1897 | 513 | 2768 | 13.90 |
| 8G | 175 | 174 | 1851 | 491 | 2691 | 13.94 |
| 39D | 194 | 175 | 1959 | 516 | 2844 | 13.95 |
| 32E | 54 | 50 | 543 | 154 | 801 | 13.96 |
| 43I | 180 | 186 | 1904 | 540 | 2810 | 14.01 |
| 36H | 170 | 220 | 2027 | 576 | 2993 | 14.01 |
| 25H | 185 | 179 | 1913 | 511 | 2788 | 14.04 |
| 24H | 169 | 182 | 1790 | 547 | 2688 | 14.04 |
| 15E | 183 | 200 | 1995 | 550 | 2928 | 14.07 |
| 34D | 171 | 183 | 1854 | 494 | 2702 | 14.09 |
| 45G | 192 | 203 | 2081 | 532 | 3008 | 14.13 |
| 49H | 200 | 179 | 1934 | 573 | 2886 | 14.13 |
| 35H | 184 | 217 | 2120 | 532 | 3053 | 14.13 |
| 45F | 157 | 132 | 1509 | 401 | 2199 | 14.14 |
| 62G | 187 | 176 | 1884 | 508 | 2755 | 14.18 |
| 26C | 178 | 203 | 1944 | 565 | 2890 | 14.19 |
| 19F | 211 | 173 | 1970 | 553 | 2907 | 14.22 |
| 19C | 162 | 189 | 1803 | 503 | 2657 | 14.22 |
| 48I | 99 | 101 | 1060 | 253 | 1513 | 14.23 |
| 8B | 201 | 164 | 1891 | 504 | 2760 | 14.24 |
| 24B | 203 | 210 | 2129 | 578 | 3120 | 14.25 |
| 33C | 178 | 198 | 1922 | 540 | 2838 | 14.27 |
| 59H | 197 | 194 | 2003 | 552 | 2946 | 14.29 |
| 62D | 199 | 181 | 1979 | 503 | 2862 | 14.30 |
| 58E | 210 | 187 | 2036 | 556 | 2989 | 14.31 |
| 20D | 80 | 76 | 793 | 222 | 1171 | 14.35 |
| 37G | 182 | 179 | 1821 | 521 | 2703 | 14.39 |
| 42F | 181 | 208 | 1979 | 541 | 2909 | 14.41 |
| 5D | 187 | 176 | 1867 | 482 | 2712 | 14.43 |
| 57I | 190 | 199 | 1948 | 569 | 2906 | 14.43 |
| 48B | 184 | 180 | 1870 | 484 | 2718 | 14.43 |
| 53E | 234 | 166 | 2028 | 557 | 2985 | 14.44 |
| 39F | 190 | 195 | 1971 | 517 | 2873 | 14.44 |
| 13F | 210 | 181 | 1972 | 552 | 2915 | 14.46 |
| 41G | 185 | 198 | 1957 | 515 | 2855 | 14.46 |
| 7A | 171 | 193 | 1874 | 474 | 2712 | 14.47 |
| 23I | 70 | 65 | 673 | 197 | 1005 | 14.48 |
| 31D | 165 | 224 | 1965 | 541 | 2895 | 14.49 |
| 1E | 167 | 185 | 1783 | 482 | 2617 | 14.50 |
| 25I | 189 | 170 | 1837 | 472 | 2668 | 14.51 |
| 48F | 185 | 159 | 1710 | 501 | 2555 | 14.52 |
| 47A | 182 | 188 | 1854 | 522 | 2746 | 14.53 |
| 58F | 215 | 186 | 2027 | 543 | 2971 | 14.56 |
| 42D | 201 | 182 | 1922 | 532 | 2837 | 14.56 |
| 29C | 184 | 183 | 1874 | 477 | 2718 | 14.56 |
| 44E | 213 | 169 | 1892 | 549 | 2823 | 14.60 |
| 28H | 188 | 169 | 1787 | 493 | 2637 | 14.60 |
| 5F | 179 | 210 | 1931 | 550 | 2870 | 14.62 |
| 4E | 184 | 174 | 1801 | 482 | 2641 | 14.62 |
| 35G | 170 | 166 | 1662 | 480 | 2478 | 14.63 |
| 42C | 201 | 172 | 1898 | 476 | 2747 | 14.65 |
| 44I | 187 | 182 | 1813 | 532 | 2714 | 14.67 |
| 47E | 199 | 194 | 1938 | 547 | 2878 | 14.74 |
| 3I | 219 | 171 | 1954 | 510 | 2854 | 14.75 |
| 41H | 203 | 198 | 1960 | 573 | 2934 | 14.76 |
| 43F | 82 | 98 | 888 | 246 | 1314 | 14.79 |
| 54E | 211 | 190 | 2016 | 507 | 2924 | 14.81 |
| 31I | 184 | 195 | 1864 | 520 | 2763 | 14.81 |
| 41C | 205 | 180 | 1922 | 498 | 2805 | 14.82 |
| 23B | 212 | 187 | 1991 | 507 | 2897 | 14.88 |
| 2G | 180 | 189 | 1828 | 482 | 2679 | 14.88 |
| 19I | 132 | 155 | 1448 | 347 | 2082 | 14.89 |
| 61G | 213 | 199 | 2023 | 552 | 2987 | 14.90 |
| 58A | 188 | 200 | 1895 | 525 | 2808 | 14.93 |
| 53G | 205 | 220 | 2097 | 552 | 3074 | 14.94 |
| 16D | 202 | 190 | 1887 | 555 | 2834 | 14.95 |
| 63F | 199 | 181 | 1883 | 484 | 2747 | 14.95 |
| 4A | 177 | 176 | 1706 | 492 | 2551 | 14.96 |
| 5G | 197 | 199 | 1970 | 493 | 2859 | 14.97 |
| 26H | 193 | 192 | 1879 | 513 | 2777 | 14.99 |
| 35E | 202 | 182 | 1867 | 516 | 2767 | 15.00 |
| 3E | 177 | 201 | 1849 | 496 | 2723 | 15.01 |
| 19E | 119 | 156 | 1338 | 360 | 1973 | 15.07 |
| 37B | 205 | 182 | 1889 | 500 | 2776 | 15.08 |
| 44G | 207 | 198 | 1954 | 542 | 2901 | 15.10 |
| 21A | 241 | 184 | 2034 | 583 | 3042 | 15.11 |
| 6B | 210 | 208 | 2018 | 554 | 2990 | 15.12 |
| 22E | 216 | 177 | 1873 | 541 | 2807 | 15.15 |
| 50H | 197 | 202 | 1908 | 542 | 2849 | 15.15 |
| 28E | 189 | 205 | 1897 | 520 | 2811 | 15.17 |
| 38I | 224 | 189 | 1988 | 542 | 2943 | 15.19 |
| 4G | 195 | 179 | 1785 | 502 | 2661 | 15.21 |
| 30I | 190 | 176 | 1764 | 474 | 2604 | 15.21 |
| 8H | 120 | 118 | 1150 | 305 | 1693 | 15.22 |
| 45E | 201 | 207 | 1953 | 540 | 2901 | 15.22 |
| 24A | 66 | 75 | 682 | 176 | 999 | 15.28 |
| 21E | 249 | 182 | 2053 | 564 | 3048 | 15.31 |
| 49C | 203 | 219 | 2015 | 542 | 2979 | 15.34 |
| 50C | 234 | 194 | 2074 | 514 | 3016 | 15.37 |
| 27H | 160 | 178 | 1637 | 404 | 2379 | 15.39 |
| 49I | 215 | 209 | 1972 | 588 | 2984 | 15.39 |
| 12H | 209 | 176 | 1805 | 518 | 2708 | 15.40 |
| 3D | 147 | 167 | 1524 | 369 | 2207 | 15.42 |
| 9F | 183 | 214 | 1956 | 437 | 2790 | 15.42 |
| 17I | 185 | 184 | 1761 | 462 | 2592 | 15.43 |
| 50G | 169 | 205 | 1758 | 493 | 2625 | 15.44 |
| 36D | 211 | 255 | 2203 | 592 | 3261 | 15.49 |
| 22B | 200 | 208 | 1947 | 497 | 2852 | 15.51 |
| 61A | 202 | 232 | 2055 | 540 | 3029 | 15.53 |
| 3G | 214 | 206 | 1977 | 534 | 2931 | 15.54 |
| 23F | 163 | 156 | 1520 | 380 | 2219 | 15.59 |
| 60H | 189 | 214 | 1901 | 497 | 2801 | 15.61 |
| 28I | 199 | 203 | 1882 | 505 | 2789 | 15.64 |
| 54A | 201 | 246 | 2076 | 578 | 3101 | 15.64 |
| 33H | 157 | 193 | 1607 | 462 | 2419 | 15.70 |
| 25A | 222 | 207 | 2002 | 531 | 2962 | 15.72 |
| 30D | 196 | 203 | 1864 | 489 | 2752 | 15.74 |
| 54D | 195 | 196 | 1782 | 519 | 2692 | 15.77 |
| 58C | 211 | 177 | 1816 | 467 | 2671 | 15.77 |
| 45I | 182 | 223 | 1873 | 502 | 2780 | 15.82 |
| 62B | 218 | 217 | 1975 | 574 | 2984 | 15.83 |
| 2C | 192 | 210 | 1869 | 484 | 2755 | 15.85 |
| 11D | 194 | 173 | 1695 | 449 | 2511 | 15.88 |
| 16G | 245 | 184 | 1975 | 530 | 2934 | 15.88 |
| 31G | 175 | 183 | 1655 | 428 | 2441 | 15.94 |
| 21B | 216 | 202 | 1909 | 516 | 2843 | 15.98 |
| 27E | 218 | 179 | 1815 | 487 | 2699 | 15.99 |
| 59G | 229 | 207 | 1979 | 549 | 2964 | 15.99 |
| 23A | 190 | 181 | 1713 | 435 | 2519 | 16.01 |
| 53C | 206 | 224 | 1944 | 544 | 2918 | 16.02 |
| 18I | 196 | 174 | 1677 | 463 | 2510 | 16.03 |
| 46C | 214 | 186 | 1844 | 469 | 2713 | 16.03 |
| 33F | 202 | 203 | 1850 | 490 | 2745 | 16.04 |
| 1H | 165 | 152 | 1445 | 383 | 2145 | 16.07 |
| 4F | 188 | 225 | 1893 | 488 | 2794 | 16.07 |
| 60G | 216 | 230 | 2047 | 524 | 3017 | 16.07 |
| 61B | 237 | 210 | 2042 | 534 | 3023 | 16.08 |
| 62I | 228 | 223 | 2063 | 532 | 3046 | 16.10 |
| 20E | 209 | 208 | 1888 | 507 | 2812 | 16.13 |
| 63G | 230 | 224 | 2074 | 533 | 3061 | 16.13 |
| 25G | 223 | 194 | 1881 | 513 | 2811 | 16.14 |
| 57G | 224 | 193 | 1859 | 534 | 2810 | 16.14 |
| 6E | 200 | 204 | 1814 | 504 | 2722 | 16.15 |
| 31E | 209 | 199 | 1828 | 491 | 2727 | 16.29 |
| 13H | 223 | 215 | 1953 | 532 | 2923 | 16.32 |
| 29F | 195 | 202 | 1752 | 494 | 2643 | 16.36 |
| 18H | 217 | 209 | 1875 | 532 | 2833 | 16.38 |
| 36F | 224 | 221 | 1969 | 544 | 2958 | 16.39 |
| 27B | 217 | 216 | 1914 | 528 | 2875 | 16.41 |
| 10D | 251 | 185 | 1909 | 546 | 2891 | 16.43 |
| 29G | 226 | 196 | 1906 | 469 | 2797 | 16.44 |
| 9A | 224 | 227 | 2013 | 525 | 2989 | 16.44 |
| 48E | 124 | 142 | 1165 | 330 | 1761 | 16.46 |
| 29H | 213 | 195 | 1831 | 462 | 2701 | 16.46 |
| 59F | 226 | 215 | 1941 | 532 | 2914 | 16.49 |
| 4I | 209 | 230 | 1954 | 497 | 2890 | 16.56 |
| 17F | 207 | 208 | 1852 | 465 | 2732 | 16.56 |
| 10F | 220 | 190 | 1840 | 445 | 2695 | 16.59 |
| 1C | 195 | 233 | 1884 | 496 | 2808 | 16.62 |
| 48D | 224 | 181 | 1830 | 422 | 2657 | 16.62 |
| 42A | 206 | 221 | 1876 | 498 | 2801 | 16.63 |
| 27C | 213 | 209 | 1860 | 486 | 2768 | 16.63 |
| 53A | 236 | 247 | 2090 | 591 | 3164 | 16.65 |
| 42G | 215 | 238 | 1997 | 515 | 2965 | 16.67 |
| 29B | 177 | 194 | 1618 | 438 | 2427 | 16.68 |
| 10E | 211 | 215 | 1864 | 490 | 2780 | 16.72 |
| 28G | 209 | 206 | 1814 | 476 | 2705 | 16.74 |
| 11G | 196 | 217 | 1804 | 474 | 2691 | 16.75 |
| 14I | 42 | 36 | 347 | 83 | 508 | 16.76 |
| 7G | 180 | 194 | 1634 | 427 | 2435 | 16.76 |
| 47C | 228 | 225 | 2013 | 482 | 2948 | 16.77 |
| 4B | 253 | 214 | 2069 | 500 | 3036 | 16.79 |
| 37A | 151 | 166 | 1371 | 364 | 2052 | 16.87 |
| 11A | 219 | 225 | 1848 | 574 | 2866 | 16.92 |
| 64D | 242 | 238 | 2064 | 554 | 3098 | 16.93 |
| 4C | 232 | 219 | 1935 | 522 | 2908 | 16.94 |
| 32F | 195 | 213 | 1739 | 483 | 2630 | 16.95 |
| 50A | 225 | 218 | 1913 | 498 | 2854 | 16.96 |
| 39C | 250 | 232 | 2096 | 524 | 3102 | 16.98 |
| 49F | 221 | 216 | 1847 | 528 | 2812 | 16.98 |
| 16B | 266 | 200 | 1977 | 555 | 2998 | 16.99 |
| 14F | 240 | 194 | 1898 | 460 | 2792 | 16.99 |
| 26E | 173 | 176 | 1471 | 423 | 2243 | 17.01 |
| 12B | 219 | 223 | 1886 | 512 | 2840 | 17.01 |
| 34F | 243 | 218 | 1989 | 509 | 2959 | 17.03 |
| 3F | 257 | 201 | 1921 | 560 | 2939 | 17.03 |
| 57B | 224 | 229 | 1932 | 521 | 2906 | 17.04 |
| 11I | 221 | 193 | 1768 | 467 | 2649 | 17.09 |
| 45C | 223 | 222 | 1888 | 514 | 2847 | 17.09 |
| 34E | 239 | 235 | 2017 | 541 | 3032 | 17.09 |
| 27I | 206 | 217 | 1803 | 475 | 2701 | 17.13 |
| 22D | 176 | 209 | 1661 | 412 | 2458 | 17.13 |
| 60D | 243 | 203 | 1915 | 483 | 2844 | 17.15 |
| 63C | 230 | 201 | 1852 | 464 | 2747 | 17.16 |
| 41I | 211 | 252 | 1971 | 514 | 2948 | 17.18 |
| 39E | 232 | 235 | 1979 | 527 | 2973 | 17.18 |
| 59D | 232 | 236 | 2020 | 490 | 2978 | 17.19 |
| 31B | 230 | 223 | 1931 | 494 | 2878 | 17.22 |
| 56A | 206 | 198 | 1726 | 433 | 2563 | 17.25 |
| 61D | 221 | 236 | 1953 | 484 | 2894 | 17.29 |
| 47D | 169 | 159 | 1410 | 338 | 2076 | 17.30 |
| 51F | 245 | 231 | 1981 | 552 | 3009 | 17.32 |
| 14A | 237 | 220 | 1922 | 505 | 2884 | 17.35 |
| 37D | 150 | 159 | 1287 | 354 | 1950 | 17.35 |
| 22A | 232 | 221 | 1884 | 521 | 2858 | 17.36 |
| 32C | 222 | 244 | 1947 | 525 | 2938 | 17.37 |
| 39A | 226 | 218 | 1867 | 482 | 2793 | 17.41 |
| 55G | 204 | 224 | 1762 | 495 | 2685 | 17.47 |
| 20C | 223 | 208 | 1742 | 527 | 2700 | 17.49 |
| 24E | 243 | 214 | 1873 | 530 | 2860 | 17.51 |
| 14G | 246 | 216 | 1890 | 534 | 2886 | 17.55 |
| 63E | 248 | 225 | 1982 | 498 | 2953 | 17.56 |
| 39H | 123 | 114 | 962 | 280 | 1479 | 17.57 |
| 1F | 216 | 242 | 1893 | 507 | 2858 | 17.57 |
| 2E | 227 | 209 | 1837 | 444 | 2717 | 17.60 |
| 61F | 207 | 207 | 1745 | 419 | 2578 | 17.61 |
| 3H | 248 | 226 | 1963 | 510 | 2947 | 17.64 |
| 28D | 199 | 202 | 1641 | 450 | 2492 | 17.65 |
| 31A | 216 | 239 | 1856 | 515 | 2826 | 17.66 |
| 1A | 214 | 245 | 1907 | 479 | 2845 | 17.70 |
| 38F | 214 | 217 | 1781 | 459 | 2671 | 17.70 |
| 16E | 234 | 242 | 1985 | 487 | 2948 | 17.72 |
| 19D | 236 | 233 | 1907 | 525 | 2901 | 17.74 |
| 7C | 235 | 221 | 1880 | 483 | 2819 | 17.75 |
| 21C | 148 | 153 | 1223 | 336 | 1860 | 17.76 |
| 23H | 213 | 229 | 1846 | 443 | 2731 | 17.76 |
| 48A | 161 | 154 | 1270 | 361 | 1946 | 17.77 |
| 63A | 171 | 185 | 1435 | 404 | 2195 | 17.80 |
| 18G | 172 | 159 | 1390 | 319 | 2040 | 17.81 |
| 43B | 234 | 241 | 1945 | 502 | 2922 | 17.85 |
| 58I | 239 | 219 | 1914 | 445 | 2817 | 17.85 |
| 19A | 239 | 241 | 1976 | 495 | 2951 | 17.86 |
| 12A | 250 | 241 | 2022 | 500 | 3013 | 17.90 |
| 1B | 237 | 228 | 1862 | 525 | 2852 | 17.91 |
| 26G | 223 | 223 | 1827 | 462 | 2735 | 17.91 |
| 25C | 114 | 100 | 857 | 241 | 1312 | 17.92 |
| 16I | 259 | 191 | 1835 | 473 | 2758 | 17.92 |
| 17A | 212 | 222 | 1723 | 496 | 2653 | 17.97 |
| 30F | 213 | 223 | 1779 | 441 | 2656 | 18.04 |
| 8I | 242 | 249 | 1983 | 516 | 2990 | 18.05 |
| 12G | 212 | 209 | 1692 | 450 | 2563 | 18.06 |
| 2B | 214 | 275 | 1947 | 522 | 2958 | 18.18 |
| 42H | 66 | 76 | 584 | 132 | 858 | 18.21 |
| 46F | 128 | 112 | 959 | 248 | 1447 | 18.25 |
| 30C | 240 | 210 | 1791 | 472 | 2713 | 18.25 |
| 31C | 244 | 245 | 1989 | 460 | 2938 | 18.32 |
| 29D | 233 | 244 | 1915 | 469 | 2861 | 18.36 |
| 6F | 205 | 236 | 1744 | 460 | 2645 | 18.36 |
| 59B | 245 | 255 | 2023 | 474 | 2997 | 18.37 |
| 18B | 225 | 241 | 1811 | 515 | 2792 | 18.38 |
| 12C | 223 | 212 | 1703 | 467 | 2605 | 18.39 |
| 23D | 234 | 234 | 1832 | 499 | 2799 | 18.42 |
| 11H | 200 | 190 | 1518 | 424 | 2332 | 18.42 |
| 44A | 135 | 146 | 1109 | 287 | 1677 | 18.46 |
| 30G | 244 | 208 | 1752 | 484 | 2688 | 18.53 |
| 61E | 249 | 283 | 2122 | 506 | 3160 | 18.56 |
| 11E | 238 | 230 | 1852 | 454 | 2774 | 18.60 |
| 22G | 290 | 232 | 2030 | 542 | 3094 | 18.60 |
| 30B | 220 | 213 | 1674 | 458 | 2565 | 18.61 |
| 20B | 73 | 79 | 586 | 162 | 900 | 18.62 |
| 8F | 281 | 244 | 2051 | 523 | 3099 | 18.69 |
| 12F | 249 | 242 | 1910 | 496 | 2897 | 18.70 |
| 16H | 269 | 256 | 2079 | 492 | 3096 | 18.71 |
| 12D | 242 | 217 | 1791 | 456 | 2706 | 18.71 |
| 22F | 170 | 196 | 1388 | 403 | 2157 | 18.72 |
| 12I | 231 | 232 | 1808 | 451 | 2722 | 18.77 |
| 26D | 215 | 252 | 1815 | 462 | 2744 | 18.78 |
| 51H | 257 | 253 | 1983 | 496 | 2989 | 18.84 |
| 40I | 276 | 240 | 2003 | 503 | 3022 | 18.85 |
| 21D | 236 | 251 | 1872 | 487 | 2846 | 18.90 |
| 16C | 240 | 235 | 1803 | 488 | 2766 | 18.97 |
| 32B | 230 | 247 | 1838 | 456 | 2771 | 19.02 |
| 41H | 153 | 170 | 1241 | 312 | 1876 | 19.03 |
| 44F | 94 | 92 | 702 | 190 | 1078 | 19.07 |
| 24I | 268 | 244 | 1976 | 479 | 2967 | 19.08 |
| 41E | 261 | 260 | 2012 | 474 | 3007 | 19.16 |
| 62H | 259 | 271 | 2002 | 526 | 3058 | 19.17 |
| 1G | 191 | 208 | 1524 | 377 | 2300 | 19.19 |
| 53H | 260 | 244 | 1908 | 489 | 2901 | 19.22 |
| 58H | 220 | 208 | 1604 | 427 | 2459 | 19.26 |
| 64A | 257 | 267 | 1992 | 491 | 3007 | 19.29 |
| 41D | 258 | 249 | 1919 | 469 | 2895 | 19.39 |
| 15B | 255 | 286 | 2026 | 522 | 3089 | 19.39 |
| 17G | 244 | 246 | 1854 | 452 | 2796 | 19.41 |
| 27A | 275 | 257 | 1942 | 553 | 3027 | 19.47 |
| 34J | 271 | 242 | 1918 | 487 | 2918 | 19.48 |
| 36E | 272 | 262 | 1985 | 515 | 3034 | 19.50 |
| 41F | 120 | 122 | 886 | 240 | 1368 | 19.61 |
| 27F | 222 | 252 | 1776 | 428 | 2678 | 19.63 |
| 7E | 249 | 229 | 1792 | 426 | 2696 | 19.66 |
| 25B | 238 | 266 | 1901 | 436 | 2841 | 19.68 |
| 22H | 169 | 204 | 1391 | 336 | 2100 | 19.70 |
| 25E | 264 | 253 | 1931 | 456 | 2904 | 19.75 |
| 33I | 275 | 265 | 2011 | 474 | 3025 | 19.81 |
| 61C | 149 | 171 | 1169 | 302 | 1791 | 19.83 |
| 62C | 245 | 270 | 1878 | 484 | 2877 | 19.88 |
| 45H | 245 | 251 | 1827 | 440 | 2763 | 19.94 |
| 17B | 276 | 293 | 2076 | 524 | 3169 | 19.94 |
| 25F | 275 | 275 | 2003 | 503 | 3056 | 20.00 |
| 34I | 256 | 255 | 1861 | 461 | 2833 | 20.05 |
| 20F | 247 | 253 | 1821 | 451 | 2772 | 20.05 |
| 27D | 226 | 267 | 1794 | 443 | 2730 | 20.07 |
| 48C | 111 | 103 | 755 | 200 | 1169 | 20.38 |
| 27G | 243 | 238 | 1738 | 402 | 2621 | 20.44 |
| 24F | 272 | 241 | 1838 | 434 | 2785 | 20.53 |
| 48G | 215 | 216 | 1499 | 389 | 2319 | 20.74 |
| 34A | 260 | 242 | 1729 | 467 | 2698 | 20.76 |
| 8E | 280 | 259 | 1836 | 521 | 2896 | 20.77 |
| 17H | 191 | 208 | 1377 | 359 | 2135 | 20.87 |
| 2F | 274 | 275 | 1914 | 472 | 2935 | 20.89 |
| 23G | 293 | 292 | 2059 | 470 | 3114 | 20.99 |
| 14B | 243 | 254 | 1722 | 408 | 2627 | 21.16 |
| 60I | 249 | 298 | 1875 | 451 | 2873 | 21.31 |
| 8C | 301 | 259 | 1866 | 505 | 2931 | 21.39 |
| 15C | 265 | 257 | 1738 | 447 | 2707 | 21.62 |
| 17D | 173 | 153 | 1047 | 313 | 1686 | 21.69 |
| 31H | 59 | 74 | 440 | 110 | 683 | 21.86 |
| 37H | 197 | 235 | 1371 | 404 | 2207 | 21.99 |
| 34C | 272 | 303 | 1885 | 472 | 2932 | 22.04 |
| 29I | 271 | 257 | 1717 | 446 | 2691 | 22.05 |
| 10A | 255 | 279 | 1733 | 453 | 2720 | 22.07 |
| 2A | 175 | 230 | 1327 | 330 | 2062 | 22.08 |
| 23C | 316 | 273 | 1903 | 492 | 2984 | 22.20 |
| 14H | 223 | 215 | 1430 | 348 | 2216 | 22.24 |
| 13B | 161 | 138 | 970 | 231 | 1500 | 22.45 |
| 2D | 281 | 247 | 1737 | 378 | 2643 | 22.51 |
| 50B | 286 | 269 | 1806 | 409 | 2770 | 22.59 |
| 22I | 309 | 265 | 1853 | 416 | 2843 | 22.79 |
| 47G | 218 | 269 | 1539 | 375 | 2401 | 22.91 |
| 19G | 251 | 214 | 1466 | 344 | 2275 | 23.11 |
| 13A | 328 | 278 | 1895 | 462 | 2963 | 23.13 |
| 24C | 286 | 292 | 1790 | 450 | 2818 | 23.20 |
| 54G | 310 | 299 | 1918 | 427 | 2954 | 23.34 |
| 38B | 248 | 245 | 1492 | 350 | 2335 | 23.99 |
| 43G | 95 | 87 | 545 | 132 | 859 | 24.09 |
| 51G | 316 | 382 | 2114 | 469 | 3281 | 24.20 |
| 26B | 320 | 289 | 1812 | 437 | 2858 | 24.25 |
| 36I | 292 | 328 | 1868 | 416 | 2904 | 24.30 |
| 16A | 299 | 304 | 1797 | 401 | 2801 | 24.54 |
| 17E | 339 | 309 | 1866 | 457 | 2971 | 24.91 |
| 10I | 236 | 197 | 1253 | 292 | 1978 | 25.02 |
| 44H | 151 | 173 | 905 | 247 | 1476 | 25.10 |
| 18E | 318 | 363 | 1953 | 394 | 3028 | 25.82 |
| 20A | 307 | 280 | 1660 | 344 | 2591 | 26.05 |
| 35F | 378 | 320 | 1897 | 438 | 3033 | 26.53 |
| 50F | 312 | 336 | 1759 | 390 | 2797 | 26.74 |
| 29E | 334 | 326 | 1774 | 414 | 2848 | 26.75 |
| 47I | 317 | 332 | 1737 | 328 | 2714 | 27.77 |
| 55C | 406 | 367 | 1949 | 418 | 3140 | 28.75 |

**Supplemental Table S7. MLH1 foci counts in *rQTL* recombinants.** To test for significant differences between genotypes, Mann-Whitney-Wilcoxon tests (MWW) were used. A significant difference was observed between the high (*rQTL1^Col^rQTL4^Ler^*) and low (*rQTL1^Ler^rQTL4^Col^*) recombination *rQTL* genotypes (MWW *P=*0.0396).

| MLH1 foci | *rQTL1^Ler^*  *rQTL4^Col^* | *rQTL1^Ler^*  *rQTL4^Ler^* | *rQTL1^Col^*  *rQTL4^Col^* | *rQTL1^Col^*  *rQTL4^Ler^* |
| --- | --- | --- | --- | --- |
| 5 | 0 | 0 | 2 | 0 |
| 6 | 4 | 4 | 2 | 1 |
| 7 | 3 | 5 | 5 | 2 |
| 8 | 8 | 7 | 9 | 3 |
| 9 | 3 | 4 | 6 | 8 |
| 10 | 2 | 1 | 5 | 2 |
| 11 | 2 | 1 | 1 | 1 |
| 12 | 1 | 0 | 2 | 2 |
| 13 | 0 | 0 | 0 | 2 |

**Supplemental Table S8. Chiasmata counts in *rQTL* recombinants.** To test for significant differences between genotypes, Mann-Whitney-Wilcoxon tests (MWW) were used. A significant difference was observed between the high (*rQTL1^Col^rQTL4^Ler^*) and low (*rQTL1^Ler^rQTL4^Col^*) recombination *rQTL* genotypes (MWW *P=*2.20×10^-5^).

| Chiasma | *rQTL1^Ler^*  *rQTL4^Col^* | *rQTL1^Ler^*  *rQTL4^Ler^* | *rQTL1^Col^*  *rQTL4^Col^* | *rQTL1^Col^*  *rQTL4^Ler^* |
| --- | --- | --- | --- | --- |
| 5 | 1 | 1 | 0 | 0 |
| 6 | 6 | 11 | 1 | 0 |
| 7 | 12 | 17 | 5 | 4 |
| 8 | 17 | 13 | 17 | 11 |
| 9 | 9 | 8 | 26 | 18 |
| 10 | 4 | 2 | 7 | 11 |
| 11 | 0 | 0 | 3 | 1 |
| 12 | 0 | 0 | 1 | 0 |

**Supplemental Table S9. *420* fluorescent count data for *rQTL* F_3_ recombinants analysed cytogenetically.** Genetic distance is calculated as cM = 100 × (1 – (1 − 2(*N_G_*+*N_R_*)/*N_T_*)^1/2^), where *N_G_* is the number of green alone seeds, *N_R_* is the number of red alone seeds and *N_T_* is the total number of seeds analysed. These data are from F_3_ individuals, the siblings of which were analysed cytogenetically (Supplemental Fig. S1 and Supplemental Tables S7-S8).

| Individual | *rQTL1* | *rQTL4* | Green alone | Red alone | Both colours | No colour | Total | cM |
| --- | --- | --- | --- | --- | --- | --- | --- | --- |
| Col | Col | Col | 157 | 159 | 1182 | 293 | 1791 | 19.56 |
| Col | Col | Col | 119 | 125 | 1023 | 249 | 1516 | 17.65 |
| Col | Col | Col | 127 | 125 | 1009 | 258 | 1519 | 18.26 |
| Col | Col | Col | 144 | 141 | 1072 | 317 | 1674 | 18.79 |
| I6 | Col | Ler | 223 | 242 | 1379 | 322 | 2166 | 24.46 |
| I6 | Col | Ler | 255 | 221 | 1251 | 282 | 2009 | 27.47 |
| I6 | Col | Ler | 223 | 207 | 1085 | 236 | 1751 | 28.67 |
| I6 | Col | Ler | 182 | 168 | 996 | 237 | 1583 | 25.31 |
| I6 | Col | Ler | 189 | 210 | 948 | 191 | 1538 | 30.64 |
| I6 | Col | Ler | 252 | 224 | 1276 | 296 | 2048 | 26.85 |
| 13E | Ler | Col | 125 | 116 | 1231 | 359 | 1831 | 14.17 |
| 13E | Ler | Col | 135 | 124 | 1443 | 391 | 2093 | 13.25 |
| 13E | Ler | Col | 141 | 120 | 1411 | 392 | 2064 | 13.57 |
| 13E | Ler | Col | 122 | 126 | 1353 | 367 | 1968 | 13.51 |
| 13E | Ler | Col | 173 | 180 | 1320 | 355 | 2028 | 19.26 |
| 13E | Ler | Col | 108 | 86 | 1079 | 269 | 1542 | 13.49 |
| 13E | Ler | Col | 99 | 92 | 977 | 275 | 1443 | 14.25 |

**Supplemental Table S10. Col-*420* LCCCC×F_2_ fluorescent count data.** Genetic distance is calculated as cM = 100 × (1 – (1 − 2(*N_G_*+*N_R_*)/*N_T_*)^1/2^), where *N_G_* is the number of green alone seeds, *N_R_* is the number of red alone seeds and *N_T_* is the total number of seeds analysed. The LCCCC parent line was obtained from an *esd7* backcross population.

| Individual | Green alone | Red alone | Both colours | No colour | Total | cM |
| --- | --- | --- | --- | --- | --- | --- |
| 1 | 95 | 102 | 957 | 216 | 1370 | 15.60 |
| 2 | 43 | 43 | 481 | 143 | 710 | 12.95 |
| 3 | 59 | 71 | 764 | 209 | 1103 | 12.58 |
| 4 | 109 | 99 | 977 | 234 | 1419 | 15.93 |
| 5 | 106 | 114 | 1177 | 316 | 1713 | 13.79 |
| 6 | 78 | 76 | 772 | 179 | 1105 | 15.07 |
| 7 | 127 | 144 | 1072 | 282 | 1625 | 18.36 |
| 8 | 126 | 145 | 1014 | 228 | 1513 | 19.89 |
| 9 | 169 | 144 | 1244 | 364 | 1921 | 17.89 |
| 10 | 122 | 142 | 1126 | 301 | 1691 | 17.07 |
| 11 | 97 | 127 | 1063 | 268 | 1555 | 15.63 |
| 12 | 138 | 137 | 1192 | 270 | 1737 | 17.33 |
| 13 | 76 | 109 | 957 | 267 | 1409 | 14.13 |
| 14 | 65 | 48 | 630 | 193 | 936 | 12.91 |
| 15 | 79 | 77 | 924 | 241 | 1321 | 12.60 |
| 16 | 102 | 114 | 1127 | 271 | 1614 | 14.42 |
| 17 | 109 | 129 | 1074 | 285 | 1597 | 16.22 |
| 18 | 97 | 81 | 988 | 277 | 1443 | 13.21 |
| 19 | 100 | 125 | 1218 | 314 | 1757 | 13.75 |
| 20 | 110 | 94 | 1151 | 270 | 1625 | 13.46 |
| 21 | 129 | 136 | 1096 | 243 | 1604 | 18.17 |
| 22 | 93 | 102 | 1040 | 285 | 1520 | 13.78 |
| 23 | 108 | 104 | 1098 | 271 | 1581 | 14.45 |
| 24 | 75 | 86 | 787 | 201 | 1149 | 15.16 |
| 25 | 103 | 85 | 1036 | 260 | 1484 | 13.59 |
| 26 | 97 | 112 | 1122 | 310 | 1641 | 13.67 |
| 27 | 70 | 78 | 726 | 212 | 1086 | 14.71 |
| 28 | 117 | 105 | 929 | 254 | 1405 | 17.30 |
| 29 | 94 | 86 | 1197 | 304 | 1681 | 11.35 |
| 30 | 78 | 79 | 680 | 183 | 1020 | 16.80 |
| 31 | 89 | 98 | 1074 | 329 | 1590 | 12.55 |
| 32 | 74 | 82 | 884 | 232 | 1272 | 13.13 |
| 33 | 99 | 97 | 1056 | 247 | 1499 | 14.06 |
| 34 | 131 | 114 | 1092 | 282 | 1619 | 16.49 |
| 35 | 109 | 112 | 1142 | 325 | 1688 | 14.08 |
| 36 | 82 | 91 | 1120 | 325 | 1618 | 11.33 |
| 37 | 138 | 164 | 1461 | 337 | 2100 | 15.60 |
| 38 | 162 | 157 | 1302 | 364 | 1985 | 17.62 |
| 39 | 98 | 109 | 1285 | 341 | 1833 | 12.01 |
| 40 | 175 | 156 | 1427 | 347 | 2105 | 17.20 |
| 41 | 145 | 138 | 1372 | 376 | 2031 | 15.07 |
| 42 | 128 | 117 | 1361 | 359 | 1965 | 13.36 |
| 43 | 134 | 144 | 1310 | 351 | 1939 | 15.55 |
| 44 | 108 | 111 | 1272 | 320 | 1811 | 12.93 |
| 45 | 163 | 151 | 1463 | 373 | 2150 | 15.86 |
| 46 | 145 | 154 | 1385 | 344 | 2028 | 16.03 |
| 47 | 176 | 159 | 1332 | 343 | 2010 | 18.35 |
| 48 | 129 | 135 | 1276 | 359 | 1899 | 15.03 |
| 49 | 164 | 123 | 1302 | 386 | 1975 | 15.78 |
| 50 | 94 | 114 | 1102 | 256 | 1566 | 14.31 |
| 51 | 123 | 136 | 1262 | 332 | 1853 | 15.12 |
| 52 | 87 | 78 | 1049 | 316 | 1530 | 11.44 |
| 53 | 140 | 130 | 1252 | 325 | 1847 | 15.88 |
| 54 | 105 | 108 | 1187 | 307 | 1707 | 13.37 |
| 55 | 121 | 138 | 1149 | 298 | 1706 | 16.55 |
| 56 | 94 | 101 | 1282 | 318 | 1795 | 11.53 |
| 57 | 149 | 136 | 1236 | 339 | 1860 | 16.72 |
| 58 | 103 | 85 | 892 | 264 | 1344 | 15.13 |
| 59 | 140 | 119 | 1105 | 280 | 1644 | 17.24 |
| 60 | 115 | 121 | 1239 | 300 | 1775 | 14.32 |
| 61 | 79 | 89 | 1001 | 278 | 1447 | 12.38 |
| 62 | 71 | 58 | 725 | 225 | 1079 | 12.77 |
| 63 | 96 | 140 | 1062 | 288 | 1586 | 16.19 |
| 64 | 111 | 125 | 1096 | 272 | 1604 | 15.99 |
| 65 | 116 | 145 | 1089 | 277 | 1627 | 17.59 |
| 66 | 92 | 104 | 1245 | 325 | 1766 | 11.79 |
| 67 | 117 | 112 | 1181 | 325 | 1735 | 14.21 |
| 68 | 144 | 151 | 1270 | 321 | 1886 | 17.10 |
| 69 | 90 | 110 | 1032 | 269 | 1501 | 14.35 |
| 70 | 112 | 118 | 1121 | 285 | 1636 | 15.22 |
| 71 | 94 | 97 | 959 | 263 | 1413 | 14.58 |
| 72 | 105 | 118 | 1111 | 301 | 1635 | 14.72 |
| 73 | 106 | 105 | 977 | 286 | 1474 | 15.52 |
| 74 | 98 | 112 | 1052 | 338 | 1600 | 14.12 |
| 75 | 109 | 92 | 1094 | 312 | 1607 | 13.41 |
| 76 | 115 | 132 | 1149 | 304 | 1700 | 15.77 |
| 77 | 106 | 147 | 1123 | 299 | 1675 | 16.46 |
| 78 | 105 | 109 | 1056 | 274 | 1544 | 14.98 |
| 79 | 93 | 97 | 1232 | 337 | 1759 | 11.46 |
| 80 | 87 | 98 | 1255 | 357 | 1797 | 10.89 |
| 81 | 115 | 72 | 1100 | 279 | 1566 | 12.75 |
| 82 | 141 | 114 | 1334 | 336 | 1925 | 14.26 |
| 83 | 144 | 130 | 1323 | 364 | 1961 | 15.11 |
| 84 | 90 | 94 | 840 | 248 | 1272 | 15.70 |
| 85 | 105 | 98 | 1140 | 328 | 1671 | 12.99 |
| 86 | 91 | 110 | 1192 | 326 | 1719 | 12.47 |
| 87 | 76 | 98 | 1222 | 312 | 1708 | 10.77 |
| 88 | 118 | 99 | 1275 | 331 | 1823 | 12.71 |
| 89 | 82 | 87 | 1112 | 316 | 1597 | 11.21 |
| 90 | 124 | 123 | 1193 | 269 | 1709 | 15.68 |
| 91 | 103 | 101 | 1161 | 312 | 1677 | 13.01 |
| 92 | 120 | 152 | 1107 | 262 | 1641 | 18.24 |
| 93 | 123 | 120 | 1170 | 302 | 1715 | 15.35 |
| 94 | 104 | 85 | 1185 | 342 | 1716 | 11.70 |
| 95 | 106 | 122 | 1219 | 305 | 1752 | 13.99 |
| 96 | 110 | 125 | 1190 | 305 | 1730 | 14.66 |
| 97 | 130 | 141 | 1162 | 287 | 1720 | 17.24 |
| 98 | 108 | 121 | 1286 | 381 | 1896 | 12.91 |
| 99 | 106 | 117 | 1050 | 285 | 1558 | 15.52 |
| 100 | 121 | 137 | 1322 | 351 | 1931 | 14.40 |
| 101 | 107 | 134 | 1046 | 269 | 1556 | 16.92 |
| 102 | 123 | 119 | 1154 | 317 | 1713 | 15.30 |
| 103 | 121 | 165 | 1250 | 338 | 1874 | 16.65 |
| 104 | 86 | 79 | 972 | 268 | 1405 | 12.53 |
| 105 | 138 | 136 | 1231 | 306 | 1811 | 16.49 |
| 106 | 118 | 105 | 1078 | 326 | 1627 | 14.80 |
| 107 | 100 | 96 | 1096 | 305 | 1597 | 13.14 |
| 108 | 96 | 84 | 881 | 247 | 1308 | 14.87 |
| 109 | 85 | 105 | 1253 | 288 | 1731 | 11.66 |
| 110 | 112 | 112 | 1089 | 306 | 1619 | 14.95 |
| 111 | 94 | 89 | 905 | 282 | 1370 | 14.39 |
| 112 | 135 | 152 | 1132 | 271 | 1690 | 18.74 |
| 113 | 117 | 128 | 1240 | 348 | 1833 | 14.40 |
| 114 | 98 | 127 | 1071 | 296 | 1592 | 15.30 |
| 115 | 118 | 125 | 1270 | 313 | 1826 | 14.34 |
| 116 | 100 | 95 | 1037 | 305 | 1537 | 13.61 |
| 117 | 126 | 100 | 1160 | 307 | 1693 | 14.38 |
| 118 | 87 | 90 | 934 | 225 | 1336 | 14.27 |
| 119 | 102 | 125 | 1008 | 270 | 1505 | 16.43 |
| 120 | 88 | 84 | 943 | 248 | 1363 | 13.54 |
| 121 | 64 | 71 | 1029 | 306 | 1470 | 9.65 |
| 122 | 114 | 100 | 1064 | 281 | 1559 | 14.83 |
| 123 | 137 | 143 | 1155 | 279 | 1714 | 17.95 |
| 124 | 102 | 87 | 1086 | 310 | 1585 | 12.74 |
| 125 | 75 | 74 | 909 | 293 | 1351 | 11.72 |
| 126 | 103 | 123 | 969 | 274 | 1469 | 16.79 |
| 127 | 84 | 106 | 1181 | 290 | 1661 | 12.18 |
| 128 | 89 | 78 | 887 | 243 | 1297 | 13.83 |
| 129 | 109 | 135 | 1094 | 246 | 1584 | 16.82 |
| 130 | 109 | 115 | 1039 | 313 | 1576 | 15.40 |
| 131 | 93 | 89 | 991 | 264 | 1437 | 13.59 |
| 132 | 90 | 94 | 975 | 268 | 1427 | 13.85 |
| 133 | 99 | 104 | 964 | 272 | 1439 | 15.27 |
| 134 | 91 | 91 | 950 | 251 | 1383 | 14.16 |
| 135 | 117 | 85 | 986 | 271 | 1459 | 14.96 |
| 136 | 97 | 91 | 904 | 234 | 1326 | 15.36 |
| 137 | 115 | 116 | 1064 | 275 | 1570 | 15.99 |
| 138 | 107 | 120 | 1016 | 253 | 1496 | 16.54 |
| 139 | 88 | 111 | 1019 | 276 | 1494 | 14.35 |
| 140 | 94 | 88 | 867 | 222 | 1271 | 15.52 |
| 141 | 102 | 74 | 913 | 224 | 1313 | 14.45 |
| 142 | 95 | 90 | 991 | 262 | 1438 | 13.82 |
| 143 | 95 | 98 | 933 | 246 | 1372 | 15.23 |
| 144 | 102 | 105 | 992 | 247 | 1446 | 15.52 |
| 145 | 116 | 120 | 1030 | 252 | 1518 | 16.99 |
| 146 | 103 | 101 | 893 | 207 | 1304 | 17.11 |
| 147 | 104 | 115 | 944 | 223 | 1386 | 17.30 |
| 148 | 126 | 121 | 1054 | 275 | 1576 | 17.14 |
| 149 | 90 | 104 | 1030 | 272 | 1496 | 13.94 |
| 150 | 102 | 94 | 1116 | 304 | 1616 | 12.97 |
| 151 | 78 | 78 | 1029 | 245 | 1430 | 11.58 |
| 152 | 102 | 101 | 1043 | 243 | 1489 | 14.72 |
| 153 | 86 | 86 | 910 | 255 | 1337 | 13.82 |
| 154 | 106 | 104 | 1016 | 287 | 1513 | 15.01 |
| 155 | 136 | 120 | 1048 | 246 | 1550 | 18.17 |
| 156 | 79 | 81 | 1003 | 285 | 1448 | 11.74 |
| 157 | 101 | 88 | 947 | 290 | 1426 | 14.27 |
| 158 | 91 | 92 | 1144 | 278 | 1605 | 12.14 |
| 159 | 94 | 118 | 912 | 274 | 1398 | 16.53 |
| 160 | 90 | 85 | 909 | 228 | 1312 | 14.37 |
| 161 | 97 | 104 | 871 | 222 | 1294 | 16.97 |
| 162 | 95 | 155 | 1131 | 248 | 1629 | 16.75 |

**Supplemental Table S11. Col-*420*×LCCCC F_3_ fluorescent count data.** Genetic distance is calculated as cM = 100 × (1 – (1 − 2(*N_G_*+*N_R_*)/*N_T_*)^1/2^), where *N_G_* is the number of green alone seeds, *N_R_* is the number of red alone seeds and *N_T_* is the total number of seeds analysed.

| Individual | Green | Red | Both | None | Total | cM |
| --- | --- | --- | --- | --- | --- | --- |
| 1_11 | 224 | 253 | 1833 | 434 | 2744 | 19.23 |
| 1_15 | 207 | 223 | 2047 | 533 | 3010 | 15.48 |
| 1_6 | 214 | 251 | 1909 | 501 | 2875 | 17.75 |
| 10_5 | 184 | 183 | 1604 | 463 | 2434 | 16.43 |
| 100_11 | 304 | 241 | 1997 | 466 | 3008 | 20.15 |
| 100_13 | 187 | 209 | 1708 | 477 | 2581 | 16.74 |
| 100_15 | 207 | 266 | 1815 | 445 | 2733 | 19.14 |
| 100_16 | 183 | 180 | 1444 | 424 | 2231 | 17.87 |
| 100_4 | 196 | 179 | 1543 | 453 | 2371 | 17.32 |
| 100_5 | 157 | 144 | 1559 | 446 | 2306 | 14.04 |
| 102_15 | 276 | 226 | 2155 | 593 | 3250 | 16.87 |
| 102_8 | 266 | 247 | 2201 | 612 | 3326 | 16.84 |
| 103_16 | 259 | 306 | 2205 | 609 | 3379 | 18.42 |
| 103_2 | 250 | 231 | 2281 | 610 | 3372 | 15.46 |
| 104_3 | 241 | 243 | 2257 | 568 | 3309 | 15.89 |
| 104_6 | 201 | 231 | 2315 | 624 | 3371 | 13.76 |
| 105_3 | 225 | 270 | 2158 | 616 | 3269 | 16.50 |
| 106_4 | 196 | 236 | 2244 | 579 | 3255 | 14.29 |
| 106_5 | 234 | 227 | 2216 | 555 | 3232 | 15.46 |
| 107_11 | 257 | 226 | 2162 | 575 | 3220 | 16.33 |
| 107_14 | 221 | 243 | 2209 | 613 | 3286 | 15.29 |
| 107_9 | 219 | 198 | 2170 | 652 | 3239 | 13.83 |
| 108_10 | 165 | 232 | 2092 | 565 | 3054 | 13.98 |
| 108_12 | 237 | 249 | 2047 | 529 | 3062 | 17.38 |
| 108_3 | 206 | 221 | 2135 | 551 | 3113 | 14.81 |
| 109_10 | 279 | 282 | 2150 | 507 | 3218 | 19.29 |
| 109_4 | 216 | 229 | 2247 | 621 | 3313 | 14.48 |
| 109_7 | 264 | 226 | 2229 | 589 | 3308 | 16.11 |
| 11_3 | 197 | 245 | 1937 | 505 | 2884 | 16.72 |
| 11_9 | 215 | 267 | 1979 | 506 | 2967 | 17.84 |
| 110_1 | 223 | 263 | 2125 | 555 | 3166 | 16.75 |
| 111_16 | 218 | 222 | 2194 | 589 | 3223 | 14.74 |
| 112_11 | 260 | 276 | 2119 | 515 | 3170 | 18.65 |
| 112_2 | 244 | 278 | 2082 | 532 | 3136 | 18.32 |
| 113_11 | 266 | 259 | 2047 | 544 | 3116 | 18.57 |
| 113_2 | 239 | 235 | 2064 | 528 | 3066 | 16.89 |
| 115_13 | 239 | 257 | 2109 | 613 | 3218 | 16.83 |
| 115_14 | 221 | 270 | 2116 | 501 | 3108 | 17.29 |
| 115_9 | 229 | 277 | 2028 | 552 | 3086 | 18.02 |
| 116_16 | 202 | 197 | 2024 | 622 | 3045 | 14.10 |
| 116_3 | 212 | 227 | 1977 | 531 | 2947 | 16.21 |
| 117_14 | 238 | 233 | 2054 | 593 | 3118 | 16.46 |
| 117_2 | 195 | 179 | 2071 | 603 | 3048 | 13.13 |
| 118_9 | 196 | 217 | 2137 | 542 | 3092 | 14.39 |
| 119_3 | 244 | 248 | 2051 | 596 | 3139 | 17.14 |
| 119_6 | 234 | 211 | 2137 | 616 | 3198 | 15.05 |
| 12_13 | 193 | 184 | 1656 | 422 | 2455 | 16.76 |
| 12_14 | 220 | 206 | 1820 | 526 | 2772 | 16.77 |
| 12_16 | 191 | 247 | 2142 | 567 | 3147 | 15.05 |
| 12_4 | 214 | 263 | 1928 | 520 | 2925 | 17.91 |
| 12_6 | 204 | 248 | 1891 | 477 | 2820 | 17.57 |
| 12_8 | 285 | 268 | 2039 | 546 | 3138 | 19.53 |
| 120_1 | 276 | 290 | 2095 | 489 | 3150 | 19.96 |
| 120_16 | 260 | 238 | 2144 | 568 | 3210 | 16.95 |
| 120_2 | 278 | 282 | 2089 | 568 | 3217 | 19.26 |
| 121_13 | 233 | 234 | 2082 | 591 | 3140 | 16.18 |
| 122_13 | 285 | 276 | 2079 | 466 | 3106 | 20.08 |
| 122_2 | 244 | 217 | 2073 | 555 | 3089 | 16.24 |
| 123_3 | 207 | 208 | 2111 | 583 | 3109 | 14.38 |
| 123_8 | 244 | 263 | 2073 | 536 | 3116 | 17.87 |
| 124_5 | 259 | 232 | 2097 | 524 | 3112 | 17.27 |
| 124_8 | 239 | 227 | 2061 | 534 | 3061 | 16.60 |
| 125_1 | 222 | 203 | 2166 | 562 | 3153 | 14.54 |
| 125_13 | 244 | 233 | 2140 | 561 | 3178 | 16.35 |
| 125_2 | 213 | 229 | 2192 | 567 | 3201 | 14.92 |
| 127_12 | 200 | 245 | 2054 | 495 | 2994 | 16.17 |
| 127_3 | 252 | 304 | 2008 | 540 | 3104 | 19.89 |
| 127_7 | 193 | 232 | 2034 | 573 | 3032 | 15.17 |
| 127_8 | 233 | 225 | 1971 | 522 | 2951 | 16.96 |
| 128_13 | 251 | 263 | 2009 | 498 | 3021 | 18.78 |
| 128_5 | 255 | 255 | 2042 | 488 | 3040 | 18.48 |
| 130_14 | 213 | 228 | 1996 | 533 | 2970 | 16.15 |
| 131_3 | 218 | 222 | 2020 | 490 | 2950 | 16.23 |
| 131_6 | 292 | 267 | 2032 | 547 | 3138 | 19.77 |
| 131_7 | 203 | 208 | 2034 | 623 | 3068 | 14.44 |
| 132_11 | 275 | 295 | 2054 | 522 | 3146 | 20.15 |
| 132_2 | 245 | 235 | 2102 | 538 | 3120 | 16.79 |
| 133_2 | 207 | 187 | 2155 | 616 | 3165 | 13.34 |
| 133_7 | 249 | 277 | 2108 | 582 | 3216 | 17.97 |
| 134_12 | 243 | 235 | 2113 | 553 | 3144 | 16.58 |
| 134_2 | 260 | 295 | 2105 | 476 | 3136 | 19.62 |
| 134_4 | 237 | 234 | 2040 | 574 | 3085 | 16.65 |
| 135_1 | 231 | 238 | 2069 | 505 | 3043 | 16.83 |
| 135_5 | 210 | 227 | 2022 | 537 | 2996 | 15.84 |
| 137_11 | 260 | 259 | 2022 | 540 | 3081 | 18.57 |
| 139_6 | 227 | 234 | 2079 | 579 | 3119 | 16.07 |
| 14_15 | 209 | 296 | 1935 | 495 | 2935 | 19.01 |
| 140_2 | 211 | 265 | 2114 | 569 | 3159 | 16.42 |
| 140_4 | 205 | 232 | 2104 | 548 | 3089 | 15.32 |
| 141_13 | 265 | 249 | 2083 | 564 | 3161 | 17.85 |
| 141_3 | 248 | 246 | 2005 | 516 | 3015 | 18.01 |
| 141_6 | 266 | 283 | 2050 | 528 | 3127 | 19.45 |
| 141_7 | 302 | 273 | 1970 | 513 | 3058 | 21.01 |
| 143_1 | 236 | 237 | 2088 | 565 | 3126 | 16.49 |
| 143_16 | 231 | 241 | 2028 | 525 | 3025 | 17.06 |
| 143_4 | 260 | 263 | 2003 | 484 | 3010 | 19.22 |
| 144_10 | 252 | 251 | 2041 | 530 | 3074 | 17.98 |
| 144_16 | 227 | 244 | 2039 | 565 | 3075 | 16.71 |
| 145_14 | 241 | 254 | 2092 | 542 | 3129 | 17.32 |
| 145_5 | 213 | 271 | 2013 | 576 | 3073 | 17.24 |
| 146_8 | 258 | 244 | 1958 | 557 | 3017 | 18.32 |
| 147_10 | 229 | 207 | 2051 | 545 | 3032 | 15.60 |
| 148_11 | 242 | 232 | 2184 | 538 | 3196 | 16.13 |
| 148_2 | 294 | 307 | 2043 | 525 | 3169 | 21.22 |
| 148_7 | 257 | 291 | 2114 | 544 | 3206 | 18.87 |
| 149_12 | 239 | 250 | 2060 | 578 | 3127 | 17.10 |
| 149_15 | 246 | 270 | 2057 | 476 | 3049 | 18.67 |
| 149_3 | 219 | 220 | 2121 | 573 | 3133 | 15.16 |
| 15_12 | 260 | 267 | 2128 | 597 | 3252 | 17.79 |
| 150_6 | 245 | 198 | 2103 | 563 | 3109 | 15.44 |
| 151_7 | 233 | 222 | 2083 | 537 | 3075 | 16.09 |
| 151_9 | 207 | 289 | 2036 | 523 | 3055 | 17.82 |
| 152_12 | 251 | 234 | 2155 | 526 | 3166 | 16.72 |
| 153_11 | 229 | 222 | 2147 | 571 | 3169 | 15.42 |
| 153_9 | 203 | 203 | 2183 | 587 | 3176 | 13.73 |
| 155_4 | 237 | 224 | 2132 | 582 | 3175 | 15.76 |
| 155_9 | 193 | 244 | 2148 | 607 | 3192 | 14.78 |
| 156_11 | 219 | 208 | 2042 | 562 | 3031 | 15.25 |
| 157_10 | 271 | 312 | 2084 | 522 | 3189 | 20.35 |
| 157_4 | 289 | 322 | 2091 | 537 | 3239 | 21.09 |
| 157_5 | 228 | 267 | 2068 | 581 | 3144 | 17.23 |
| 158_10 | 228 | 227 | 2062 | 568 | 3085 | 16.03 |
| 158_4 | 250 | 253 | 2028 | 563 | 3094 | 17.85 |
| 159_13 | 296 | 295 | 2085 | 491 | 3167 | 20.83 |
| 160_1 | 245 | 234 | 2009 | 584 | 3072 | 17.05 |
| 160_6 | 235 | 261 | 2048 | 533 | 3077 | 17.68 |
| 160_8 | 190 | 218 | 2151 | 567 | 3126 | 14.04 |
| 161_16 | 240 | 262 | 2064 | 517 | 3083 | 17.88 |
| 164_11 | 191 | 219 | 2062 | 590 | 3062 | 14.43 |
| 166_13 | 257 | 259 | 2023 | 537 | 3076 | 18.48 |
| 166_4 | 216 | 253 | 2114 | 533 | 3116 | 16.40 |
| 169_1 | 225 | 298 | 2044 | 521 | 3088 | 18.68 |
| 169_13 | 247 | 306 | 2071 | 519 | 3143 | 19.49 |
| 169_2 | 236 | 262 | 1994 | 591 | 3083 | 17.72 |
| 169_3 | 267 | 236 | 2127 | 542 | 3172 | 17.37 |
| 17_3 | 242 | 264 | 2110 | 545 | 3161 | 17.55 |
| 170_1 | 211 | 275 | 2119 | 500 | 3105 | 17.12 |
| 170_3 | 289 | 293 | 2098 | 529 | 3209 | 20.17 |
| 170_5 | 263 | 306 | 2183 | 571 | 3323 | 18.91 |
| 171_7 | 251 | 224 | 2147 | 593 | 3215 | 16.06 |
| 172_7 | 240 | 233 | 2283 | 577 | 3333 | 15.37 |
| 173_1 | 224 | 221 | 2128 | 587 | 3160 | 15.24 |
| 173_11 | 245 | 248 | 2104 | 608 | 3205 | 16.79 |
| 173_6 | 283 | 268 | 2010 | 542 | 3103 | 19.70 |
| 175_5 | 302 | 247 | 2043 | 551 | 3143 | 19.34 |
| 176_11 | 238 | 224 | 2094 | 601 | 3157 | 15.90 |
| 176_13 | 227 | 212 | 2105 | 563 | 3107 | 15.30 |
| 176_9 | 294 | 291 | 2084 | 530 | 3199 | 20.36 |
| 178_13 | 298 | 265 | 2086 | 531 | 3180 | 19.63 |
| 179_6 | 259 | 229 | 2079 | 537 | 3104 | 17.20 |
| 18_5 | 272 | 256 | 2175 | 531 | 3234 | 17.93 |
| 180_11 | 219 | 229 | 2148 | 602 | 3198 | 15.16 |
| 180_13 | 208 | 205 | 2210 | 617 | 3240 | 13.68 |
| 182_13 | 235 | 230 | 2135 | 540 | 3140 | 16.11 |
| 183_14 | 229 | 232 | 2092 | 598 | 3151 | 15.89 |
| 184_6 | 237 | 246 | 2080 | 571 | 3134 | 16.83 |
| 185_1 | 308 | 253 | 2120 | 551 | 3232 | 19.20 |
| 185_11 | 293 | 276 | 2080 | 527 | 3176 | 19.89 |
| 185_3 | 245 | 249 | 2157 | 593 | 3244 | 16.61 |
| 185_6 | 266 | 232 | 2182 | 563 | 3243 | 16.76 |
| 185_8 | 234 | 223 | 2077 | 584 | 3118 | 15.92 |
| 186_11 | 252 | 264 | 2079 | 539 | 3134 | 18.10 |
| 186_14 | 271 | 260 | 2105 | 572 | 3208 | 18.21 |
| 186_8 | 250 | 236 | 2071 | 505 | 3062 | 17.38 |
| 186_9 | 214 | 237 | 2127 | 610 | 3188 | 15.32 |
| 187_14 | 300 | 289 | 2047 | 521 | 3157 | 20.83 |
| 187_15 | 237 | 235 | 2114 | 515 | 3101 | 16.60 |
| 187_16 | 211 | 226 | 2087 | 577 | 3101 | 15.26 |
| 187_3 | 257 | 248 | 2057 | 528 | 3090 | 17.95 |
| 187_7 | 227 | 235 | 2092 | 532 | 3086 | 16.30 |
| 188_11 | 242 | 274 | 2072 | 599 | 3187 | 17.77 |
| 188_7 | 232 | 255 | 2066 | 527 | 3080 | 17.31 |
| 188_8 | 138 | 184 | 1677 | 389 | 2388 | 14.54 |
| 188_9 | 256 | 272 | 2112 | 555 | 3195 | 18.18 |
| 189_11 | 229 | 232 | 2123 | 536 | 3120 | 16.07 |
| 19_11 | 171 | 230 | 1808 | 463 | 2672 | 16.34 |
| 19_15 | 179 | 217 | 1870 | 494 | 2760 | 15.56 |
| 19_9 | 243 | 252 | 2066 | 506 | 3067 | 17.71 |
| 190_3 | 229 | 244 | 2016 | 548 | 3037 | 17.02 |
| 192_10 | 262 | 256 | 2013 | 516 | 3047 | 18.76 |
| 192_11 | 247 | 263 | 2051 | 558 | 3119 | 17.97 |
| 192_14 | 218 | 223 | 2071 | 585 | 3097 | 15.43 |
| 193_10 | 225 | 250 | 2023 | 569 | 3067 | 16.92 |
| 193_12 | 206 | 185 | 2098 | 607 | 3096 | 13.55 |
| 193_15 | 277 | 309 | 2034 | 479 | 3099 | 21.14 |
| 194_10 | 231 | 254 | 2039 | 515 | 3039 | 17.49 |
| 194_2 | 215 | 239 | 2021 | 564 | 3039 | 16.26 |
| 195_5 | 214 | 265 | 2046 | 592 | 3117 | 16.77 |
| 196_8 | 231 | 288 | 2040 | 542 | 3101 | 18.44 |
| 197_14 | 243 | 240 | 1963 | 498 | 2944 | 18.03 |
| 198_1 | 198 | 187 | 2097 | 589 | 3071 | 13.44 |
| 199_11 | 185 | 205 | 2015 | 525 | 2930 | 14.34 |
| 199_14 | 245 | 223 | 1982 | 533 | 2983 | 17.16 |
| 199_16 | 230 | 209 | 1972 | 510 | 2921 | 16.37 |
| 2_4 | 218 | 188 | 1719 | 465 | 2590 | 17.15 |
| 2_8 | 238 | 243 | 2107 | 575 | 3163 | 16.58 |
| 20_11 | 252 | 221 | 2164 | 572 | 3209 | 16.02 |
| 200_1 | 178 | 199 | 2010 | 516 | 2903 | 13.96 |
| 201_14 | 216 | 253 | 1934 | 477 | 2880 | 17.88 |
| 201_2 | 236 | 226 | 1920 | 544 | 2926 | 17.28 |
| 201_9 | 234 | 252 | 1963 | 454 | 2903 | 18.44 |
| 202_7 | 162 | 212 | 1815 | 528 | 2717 | 14.87 |
| 21_16 | 242 | 264 | 2056 | 530 | 3092 | 17.98 |
| 22_14 | 215 | 238 | 2277 | 581 | 3311 | 14.77 |
| 22_2 | 323 | 313 | 2221 | 582 | 3439 | 20.62 |
| 22_4 | 253 | 250 | 2209 | 538 | 3250 | 16.91 |
| 23_6 | 216 | 247 | 2175 | 573 | 3211 | 15.64 |
| 24_12 | 295 | 245 | 2192 | 561 | 3293 | 18.02 |
| 25_4 | 236 | 247 | 1970 | 511 | 2964 | 17.90 |
| 25_5 | 280 | 290 | 2028 | 453 | 3051 | 20.86 |
| 26_1 | 285 | 301 | 2129 | 460 | 3175 | 20.57 |
| 26_16 | 203 | 221 | 1405 | 368 | 2197 | 21.64 |
| 27_7 | 299 | 291 | 2086 | 533 | 3209 | 20.48 |
| 28_13 | 239 | 289 | 2195 | 578 | 3301 | 17.53 |
| 28_15 | 263 | 224 | 2299 | 624 | 3410 | 15.48 |
| 29_2 | 252 | 257 | 2082 | 488 | 3079 | 18.18 |
| 29_4 | 211 | 220 | 2070 | 540 | 3041 | 15.35 |
| 3_11 | 227 | 224 | 2117 | 548 | 3116 | 15.71 |
| 3_4 | 235 | 281 | 2021 | 490 | 3027 | 18.82 |
| 30_10 | 216 | 229 | 2015 | 518 | 2978 | 16.27 |
| 31_1 | 213 | 278 | 2060 | 533 | 3084 | 17.44 |
| 31_4 | 270 | 244 | 2030 | 464 | 3008 | 18.87 |
| 32_9 | 207 | 247 | 1992 | 566 | 3012 | 16.42 |
| 34_10 | 195 | 236 | 2053 | 540 | 3024 | 15.45 |
| 34_16 | 241 | 275 | 1968 | 507 | 2991 | 19.07 |
| 35_1 | 212 | 283 | 1978 | 489 | 2962 | 18.41 |
| 36_12 | 255 | 287 | 2012 | 494 | 3048 | 19.73 |
| 36_14 | 190 | 189 | 1617 | 480 | 2476 | 16.70 |
| 37_11 | 273 | 276 | 2098 | 535 | 3182 | 19.07 |
| 37_14 | 279 | 290 | 2078 | 551 | 3198 | 19.74 |
| 37_2 | 252 | 271 | 2032 | 516 | 3071 | 18.80 |
| 39_1 | 186 | 206 | 1613 | 431 | 2436 | 17.65 |
| 4_16 | 239 | 183 | 1871 | 479 | 2772 | 16.60 |
| 4_9 | 227 | 256 | 2081 | 563 | 3127 | 16.87 |
| 41_1 | 235 | 247 | 2089 | 559 | 3130 | 16.81 |
| 42_2 | 225 | 214 | 2073 | 610 | 3122 | 15.22 |
| 43_13 | 222 | 221 | 1971 | 510 | 2924 | 16.51 |
| 43_15 | 250 | 224 | 2210 | 597 | 3281 | 15.68 |
| 44_2 | 193 | 194 | 1444 | 407 | 2238 | 19.12 |
| 44_4 | 254 | 228 | 2031 | 544 | 3057 | 17.26 |
| 45_1 | 202 | 282 | 1995 | 500 | 2979 | 17.84 |
| 45_13 | 327 | 284 | 1920 | 486 | 3017 | 22.87 |
| 45_2 | 285 | 219 | 2040 | 502 | 3046 | 18.20 |
| 45_3 | 183 | 190 | 1589 | 409 | 2371 | 17.21 |
| 47_3 | 234 | 247 | 2036 | 532 | 3049 | 17.27 |
| 47_9 | 227 | 239 | 2035 | 543 | 3044 | 16.70 |
| 5_1 | 240 | 176 | 1786 | 447 | 2649 | 17.18 |
| 5_10 | 214 | 233 | 1907 | 540 | 2894 | 16.87 |
| 5_3 | 246 | 243 | 2071 | 515 | 3075 | 17.42 |
| 50_12 | 239 | 270 | 2046 | 498 | 3053 | 18.36 |
| 51_9 | 236 | 236 | 2188 | 586 | 3246 | 15.79 |
| 52_2 | 281 | 222 | 2228 | 564 | 3295 | 16.65 |
| 53_14 | 247 | 250 | 2297 | 595 | 3389 | 15.93 |
| 53_15 | 280 | 257 | 2168 | 526 | 3231 | 18.29 |
| 53_4 | 297 | 334 | 2138 | 514 | 3283 | 21.54 |
| 55_12 | 272 | 312 | 1971 | 429 | 2984 | 21.99 |
| 55_8 | 263 | 280 | 2097 | 513 | 3153 | 19.03 |
| 56_14 | 295 | 249 | 2036 | 524 | 3104 | 19.41 |
| 56_6 | 217 | 192 | 1935 | 520 | 2864 | 15.48 |
| 57_15 | 272 | 293 | 2099 | 548 | 3212 | 19.49 |
| 58_10 | 225 | 238 | 2052 | 530 | 3045 | 16.58 |
| 58_5 | 193 | 294 | 1953 | 490 | 2930 | 18.29 |
| 59_8 | 262 | 272 | 2117 | 511 | 3162 | 18.62 |
| 6_15 | 256 | 228 | 2087 | 572 | 3143 | 16.81 |
| 60_14 | 307 | 287 | 1928 | 450 | 2972 | 22.52 |
| 60_2 | 283 | 263 | 2024 | 527 | 3097 | 19.54 |
| 60_7 | 298 | 254 | 2154 | 504 | 3210 | 19.00 |
| 61_2 | 238 | 219 | 1631 | 426 | 2514 | 20.22 |
| 61_3 | 168 | 128 | 1276 | 343 | 1915 | 16.88 |
| 61_6 | 243 | 280 | 2095 | 569 | 3187 | 18.04 |
| 63_10 | 242 | 259 | 2130 | 584 | 3215 | 17.03 |
| 63_4 | 307 | 289 | 2054 | 474 | 3124 | 21.36 |
| 64_11 | 266 | 228 | 2094 | 553 | 3141 | 17.21 |
| 64_4 | 266 | 210 | 1981 | 502 | 2959 | 17.64 |
| 65_11 | 253 | 241 | 2148 | 535 | 3177 | 16.99 |
| 65_5 | 279 | 272 | 1994 | 467 | 3012 | 20.37 |
| 66_16 | 210 | 234 | 1579 | 365 | 2388 | 20.74 |
| 69_1 | 272 | 270 | 2133 | 568 | 3243 | 18.41 |
| 69_10 | 272 | 337 | 2016 | 459 | 3084 | 22.21 |
| 7_2 | 236 | 245 | 2050 | 595 | 3126 | 16.80 |
| 71_15 | 280 | 288 | 2139 | 565 | 3272 | 19.20 |
| 71_6 | 303 | 324 | 1958 | 499 | 3084 | 22.97 |
| 72_4 | 268 | 256 | 1827 | 458 | 2809 | 20.82 |
| 72_8 | 265 | 253 | 2040 | 495 | 3053 | 18.72 |
| 75_2 | 181 | 160 | 1733 | 488 | 2562 | 14.34 |
| 76_3 | 280 | 287 | 2120 | 561 | 3248 | 19.32 |
| 78_14 | 255 | 222 | 2133 | 612 | 3222 | 16.10 |
| 78_2 | 242 | 234 | 2088 | 553 | 3117 | 16.66 |
| 78_9 | 199 | 233 | 2072 | 566 | 3070 | 15.23 |
| 79_15 | 297 | 250 | 2039 | 520 | 3106 | 19.52 |
| 79_16 | 202 | 205 | 1666 | 453 | 2526 | 17.67 |
| 79_4 | 196 | 194 | 1713 | 512 | 2615 | 16.23 |
| 79_7 | 298 | 250 | 2100 | 483 | 3131 | 19.38 |
| 80_12 | 268 | 264 | 1947 | 457 | 2936 | 20.15 |
| 80_13 | 250 | 267 | 2067 | 510 | 3094 | 18.40 |
| 82_16 | 218 | 168 | 1757 | 490 | 2633 | 15.93 |
| 89_3 | 218 | 200 | 1688 | 384 | 2490 | 18.50 |
| 89_9 | 173 | 237 | 1745 | 435 | 2590 | 17.33 |
| 9_16 | 308 | 279 | 2112 | 535 | 3234 | 20.19 |
| 9_4 | 287 | 264 | 2134 | 534 | 3219 | 18.90 |
| 91_1 | 258 | 228 | 1685 | 431 | 2602 | 20.85 |
| 91_2 | 312 | 204 | 2143 | 543 | 3202 | 17.68 |
| 93_2 | 304 | 276 | 2150 | 541 | 3271 | 19.67 |
| 94_1 | 259 | 230 | 2056 | 568 | 3113 | 17.18 |
| 95_11 | 291 | 269 | 1978 | 460 | 2998 | 20.85 |
| 96_7 | 305 | 240 | 2086 | 510 | 3141 | 19.19 |
| 98_3 | 265 | 254 | 2095 | 463 | 3077 | 18.60 |

**Supplemental Table S12. Sanger sequencing analysis of *HEI10* polymorphisms.** Position coordinates are given for the TAIR10 reference assembly. Decimal values are used when an insertion/deletion polymorphism is observed between a given accession and Col-0. The *HEI10* start codon is located at 19,965,146 bp. The *rQTL1* candidate column indicates ‘Yes’ if a polymorphism was shared between Ler-0, Bur-0 and Cvi-0 but not with Ct-1.

| Position | Col-0 | Ler-0 | Cvi-0 | Ct-1 | Bur-0 | Non-synonymous | *rQTL1* |
| --- | --- | --- | --- | --- | --- | --- | --- |
| 19961828 | G | G | A | A | G | No | No |
| 19961840 | T | TT | T | T | TT | No | No |
| 19961853 | A | T | T | T | T | No | No |
| 19961889 | T | A | T | T | A | No | No |
| 19961903.5 | - | - | T | - | - | No | No |
| 19961985 | A | A | A | A | T | No | No |
| 19962239 | G | G | G | A | G | No | No |
| 19962254 | T | G | T | T | G | No | No |
| 19962525 | G | G | G | C | G | No | No |
| 19962610 | G | T | T | T | T | No | No |
| 19962626 | A | G | G | G | G | No | No |
| 19962643 | T | T | A | A | T | No | No |
| 19962883.5 | - | A | - | - | A | No | No |
| 19962885 | T | A | T | T | A | No | No |
| 19962916 | G | G | G | A | G | No | No |
| 19962983 | T | T | A | A | T | No | No |
| 19962990 | C | C | C | T | C | No | No |
| 19963043 | TGTC-------T | TGTCTTTTTTTT | ------------ | TGTC-------T | TGTC-TTTTTTT | No | Yes |
| 19963055 | T | T | A | T | T | No | No |
| 19963056 | T | C | T | C | C | No | No |
| 19963060 | T | T | A | T | T | No | No |
| 19963084 | C | T | T | C | T | No | Yes |
| 19963105 | G | G | G | A | G | No | No |
| 19963152 | T | G | G | T | G | No | Yes |
| 19963171 | T | T | A | T | T | No | No |
| 19963195 | G | A | G | G | A | No | No |
| 19963201 | C | T | C | C | T | No | No |
| 19963218 | C | C | T | C | C | No | No |
| 19963286 | G | A | G | G | A | No | No |
| 19963290 | C | C | T | C | C | No | No |
| 19963300 | T | A | A | T | A | No | Yes |
| 19963403 | C | C | T | C | C | No | No |
| 19963414 | C | C | A | C | C | No | No |
| 19963423 | G | G | A | G | G | No | No |
| 19963453 | G | G | A | G | G | No | No |
| 19963487 | G | T | G | G | T | No | No |
| 19963644 | T | T | A | T | T | No | No |
| 19963646 | C | G | G | C | G | No | Yes |
| 19963688 | C | C | A | C | C | No | No |
| 19963692 | A | A | C | A | A | No | No |
| 19963741 | G | G | A | G | G | No | No |
| 19963816 | C | T | C | C | T | No | No |
| 19963857 | T | T | C | T | T | No | No |
| 19963946 | C | C | T | C | C | No | No |
| 19963956 | A | G | G | A | G | No | Yes |
| 19963971.5 | - | A | - | - | - | No | No |
| 19963990.5 | - | - | GAAGAGAG | - | - | No | No |
| 19964001 | C | T | C | C | C | No | No |
| 19964033 | A | A | G | A | A | No | No |
| 19964061 | A | G | G | A | G | No | Yes |
| 19964113 | T | C | C | T | C | No | Yes |
| 19964115.5 | - | A | A | - | A | No | Yes |
| 19964245 | C | T | T | C | T | No | Yes |
| 19964535 | A | A | G | A | A | No | No |
| 19964647 | A | A | T | A | A | No | No |
| 19964949 | G | G | A | G | G | No | No |
| 19965279 | A | A | G | A | A | No | No |
| 19965686 | G | G | A | G | G | No | No |
| 19966347 | A | G | A | A | G | No | No |
| 19966367 | G | G | A | G | G | No | No |
| 19966374 | G | A | G | G | A | No | No |
| 19966492 | G | G | G | A | G | No | No |
| 19966525 | A | G | G | A | G | No | Yes |
| 19966592 | A | G | G | A | G | Yes | Yes |
| 19967091.5 | - | A | - | - | A | No | No |
| 19967101 | C | C | G | C | C | No | No |

**Supplemental Table S13. Col-*420*×Bur-0 F_2_ fluorescent count data.** Genetic distance is calculated as cM = 100 × (1 – (1 − 2(*N_G_*+*N_R_*)/*N_T_*)^1/2^), where *N_G_* is the number of green alone seeds, *N_R_* is the number of red alone seeds and *N_T_* is the total number of seeds analysed.

| Individual | Green alone | Red alone | Both colours | No colour | Total | cM |
| --- | --- | --- | --- | --- | --- | --- |
| 1A3 | 102 | 102 | 1191 | 325 | 1720 | 12.66 |
| 1A5 | 142 | 113 | 1521 | 449 | 2225 | 12.21 |
| 1B2 | 35 | 49 | 779 | 211 | 1074 | 8.15 |
| 1B4 | 88 | 117 | 819 | 192 | 1216 | 18.59 |
| 1B5 | 61 | 88 | 922 | 266 | 1337 | 11.85 |
| 1B6 | 125 | 167 | 1384 | 352 | 2028 | 15.62 |
| 1C1 | 12 | 17 | 232 | 74 | 335 | 9.07 |
| 1C2 | 108 | 140 | 1241 | 336 | 1825 | 14.66 |
| 1C3 | 86 | 96 | 597 | 122 | 901 | 22.80 |
| 1C5 | 59 | 82 | 1105 | 325 | 1571 | 9.42 |
| 1D3 | 72 | 93 | 827 | 195 | 1187 | 15.03 |
| 1E2 | 193 | 166 | 1278 | 314 | 1951 | 20.50 |
| 1E3 | 107 | 99 | 945 | 219 | 1370 | 16.38 |
| 1E5 | 124 | 127 | 1074 | 204 | 1529 | 18.04 |
| 1E6 | 195 | 169 | 1355 | 301 | 2020 | 20.02 |
| 1F3 | 150 | 114 | 1409 | 371 | 2044 | 13.88 |
| 1F4 | 158 | 173 | 1341 | 303 | 1975 | 18.46 |
| 1F5 | 163 | 156 | 1448 | 371 | 2138 | 16.24 |
| 1F6 | 117 | 120 | 1401 | 349 | 1987 | 12.74 |
| 1G1 | 83 | 95 | 922 | 249 | 1349 | 14.20 |
| 1G4 | 147 | 176 | 1327 | 327 | 1977 | 17.95 |
| 1G5 | 191 | 175 | 1240 | 257 | 1863 | 22.08 |
| 1H1 | 116 | 124 | 974 | 235 | 1449 | 18.22 |
| 1H2 | 84 | 93 | 915 | 247 | 1339 | 14.23 |
| 1H4 | 117 | 106 | 988 | 272 | 1483 | 16.38 |
| 1H5 | 140 | 135 | 1390 | 340 | 2005 | 14.81 |
| 1H6 | 96 | 92 | 1315 | 382 | 1885 | 10.53 |
| 2A3 | 125 | 119 | 1094 | 279 | 1617 | 16.44 |
| 2B5 | 172 | 166 | 1388 | 333 | 2059 | 18.04 |
| 2B6 | 123 | 134 | 1182 | 313 | 1752 | 15.94 |
| 2C1 | 66 | 63 | 1124 | 336 | 1589 | 8.48 |
| 2C3 | 133 | 126 | 1186 | 303 | 1748 | 16.12 |
| 2C4 | 84 | 118 | 889 | 226 | 1317 | 16.74 |
| 2C5 | 111 | 98 | 746 | 192 | 1147 | 20.28 |
| 2D1 | 55 | 89 | 1072 | 286 | 1502 | 10.10 |
| 2D2 | 74 | 79 | 1129 | 303 | 1585 | 10.17 |
| 2D3 | 56 | 67 | 905 | 262 | 1290 | 10.04 |
| 2D5 | 130 | 121 | 1133 | 299 | 1683 | 16.23 |
| 2D6 | 115 | 126 | 1382 | 375 | 1998 | 12.89 |
| 2E3 | 88 | 96 | 1269 | 357 | 1810 | 10.74 |
| 2E4 | 161 | 159 | 1277 | 353 | 1950 | 18.04 |
| 2E5 | 64 | 90 | 999 | 300 | 1453 | 11.23 |
| 2E6 | 33 | 40 | 693 | 197 | 963 | 7.89 |
| 2F4 | 124 | 133 | 1137 | 284 | 1678 | 16.71 |
| 2G1 | 94 | 103 | 1293 | 308 | 1798 | 11.63 |
| 2G3 | 119 | 134 | 1182 | 317 | 1752 | 15.67 |
| 2G4 | 70 | 65 | 1121 | 314 | 1570 | 9.00 |
| 2G6 | 163 | 165 | 1314 | 326 | 1968 | 18.35 |
| 2H2 | 79 | 74 | 935 | 241 | 1329 | 12.26 |
| 2H4 | 196 | 180 | 1316 | 338 | 2030 | 20.66 |
| 3A1 | 133 | 122 | 1074 | 255 | 1584 | 17.66 |
| 3A3 | 86 | 101 | 741 | 163 | 1091 | 18.93 |
| 3A4 | 121 | 154 | 1335 | 327 | 1937 | 15.38 |
| 3A6 | 60 | 65 | 1099 | 298 | 1522 | 8.58 |
| 3B3 | 104 | 128 | 1193 | 303 | 1728 | 14.47 |
| 3C2 | 63 | 69 | 1174 | 327 | 1633 | 8.44 |
| 3C6 | 112 | 148 | 1316 | 350 | 1926 | 14.56 |
| 3D2 | 136 | 153 | 1439 | 412 | 2140 | 14.57 |
| 3D3 | 142 | 151 | 1392 | 351 | 2036 | 15.61 |
| 3D4 | 111 | 117 | 1248 | 288 | 1764 | 13.89 |
| 3E1 | 190 | 157 | 1017 | 213 | 1577 | 25.17 |
| 3E3 | 71 | 87 | 1077 | 315 | 1550 | 10.77 |
| 3E5 | 122 | 138 | 1121 | 232 | 1613 | 17.68 |
| 3F1 | 102 | 103 | 1052 | 274 | 1531 | 14.43 |
| 3F3 | 106 | 107 | 1399 | 370 | 1982 | 11.40 |
| 3G2 | 103 | 133 | 1497 | 427 | 2160 | 11.60 |
| 3G5 | 158 | 178 | 1247 | 306 | 1889 | 19.73 |
| 3H1 | 81 | 71 | 947 | 250 | 1349 | 11.99 |
| 4A1 | 119 | 150 | 1083 | 240 | 1592 | 18.63 |
| 4A3 | 35 | 59 | 751 | 219 | 1064 | 9.26 |
| 4A5 | 144 | 155 | 1409 | 402 | 2110 | 15.35 |
| 4A6 | 80 | 106 | 1065 | 259 | 1510 | 13.19 |
| 4B1 | 127 | 151 | 1377 | 346 | 2001 | 15.02 |
| 4B3 | 68 | 88 | 866 | 265 | 1287 | 12.96 |
| 4B7 | 103 | 106 | 1074 | 229 | 1512 | 14.94 |
| 4C1 | 163 | 168 | 1179 | 256 | 1766 | 21.40 |
| 4C2 | 133 | 124 | 1220 | 287 | 1764 | 15.82 |
| 4C3 | 114 | 142 | 1397 | 361 | 2014 | 13.64 |
| 4D1 | 106 | 80 | 1100 | 280 | 1566 | 12.68 |
| 4D2 | 113 | 128 | 1102 | 238 | 1581 | 16.63 |
| 4D3 | 61 | 59 | 734 | 210 | 1064 | 12.00 |
| 4D4 | 130 | 116 | 1337 | 348 | 1931 | 13.67 |
| 4D6 | 91 | 100 | 997 | 270 | 1458 | 14.09 |
| 4D8 | 102 | 100 | 931 | 239 | 1372 | 16.00 |
| 4E4 | 152 | 152 | 1326 | 286 | 1916 | 17.38 |
| 4E7 | 69 | 55 | 1336 | 379 | 1839 | 6.99 |
| 4E8 | 162 | 171 | 1299 | 286 | 1918 | 19.21 |
| 4F1 | 52 | 78 | 646 | 192 | 968 | 14.48 |
| 4F2 | 85 | 103 | 748 | 181 | 1117 | 18.55 |
| 4F6 | 129 | 152 | 1357 | 356 | 1994 | 15.26 |
| 4F8 | 149 | 155 | 1437 | 369 | 2110 | 15.63 |
| 4G3 | 75 | 63 | 744 | 202 | 1084 | 13.66 |
| 4G7 | 163 | 143 | 1408 | 354 | 2068 | 16.09 |
| 4H3 | 176 | 150 | 1314 | 338 | 1978 | 18.12 |
| 4H4 | 68 | 80 | 1160 | 336 | 1644 | 9.45 |
| 4H5 | 137 | 177 | 1280 | 308 | 1902 | 18.16 |
| 4H8 | 59 | 79 | 1479 | 437 | 2054 | 6.96 |
| 5A3 | 134 | 142 | 983 | 274 | 1533 | 20.00 |
| 5A4 | 147 | 163 | 1250 | 331 | 1891 | 18.02 |
| 5A6 | 123 | 133 | 1378 | 341 | 1975 | 13.93 |
| 5A7 | 123 | 120 | 1160 | 272 | 1675 | 15.75 |
| 5A8 | 69 | 60 | 555 | 125 | 809 | 17.47 |
| 5A9 | 142 | 175 | 1631 | 439 | 2387 | 14.30 |
| 5B1 | 84 | 95 | 1380 | 381 | 1940 | 9.70 |
| 5B2 | 44 | 50 | 863 | 242 | 1199 | 8.17 |
| 5B6 | 49 | 50 | 502 | 146 | 747 | 14.27 |
| 5B7 | 197 | 192 | 1345 | 281 | 2015 | 21.65 |
| 5B8 | 230 | 214 | 1581 | 404 | 2429 | 20.35 |
| 5B9 | 109 | 116 | 1217 | 323 | 1765 | 13.68 |
| 5C1 | 72 | 72 | 1088 | 352 | 1584 | 9.55 |
| 5C2 | 99 | 98 | 716 | 164 | 1077 | 20.37 |
| 5C3 | 80 | 88 | 1222 | 339 | 1729 | 10.24 |
| 5C5 | 72 | 73 | 820 | 200 | 1165 | 13.34 |
| 5C7 | 95 | 127 | 1617 | 462 | 2301 | 10.16 |
| 5C8 | 115 | 129 | 1321 | 340 | 1905 | 13.75 |
| 5D1 | 92 | 94 | 999 | 279 | 1464 | 13.63 |
| 5D2 | 99 | 137 | 1067 | 292 | 1595 | 16.09 |
| 5D3 | 79 | 96 | 1298 | 397 | 1870 | 9.84 |
| 5D6 | 84 | 131 | 1249 | 337 | 1801 | 12.75 |
| 5D7 | 132 | 157 | 1599 | 450 | 2338 | 13.24 |
| 5D9 | 120 | 108 | 1045 | 322 | 1595 | 15.50 |
| 5E1 | 128 | 130 | 1040 | 284 | 1582 | 17.91 |
| 5E2 | 78 | 90 | 732 | 211 | 1111 | 16.48 |
| 5E3 | 109 | 105 | 1030 | 263 | 1507 | 15.38 |
| 5E4 | 178 | 182 | 1353 | 312 | 2025 | 19.72 |
| 5E5 | 88 | 89 | 917 | 229 | 1323 | 14.42 |
| 5E8 | 170 | 203 | 1484 | 373 | 2230 | 18.42 |
| 5E9 | 137 | 110 | 1211 | 337 | 1795 | 14.87 |
| 5F3 | 137 | 160 | 1068 | 286 | 1651 | 19.99 |
| 5F5 | 42 | 45 | 660 | 214 | 961 | 9.50 |
| 5F6 | 138 | 124 | 1063 | 259 | 1584 | 18.20 |
| 5F8 | 140 | 166 | 1343 | 350 | 1999 | 16.70 |
| 5G2 | 84 | 101 | 728 | 162 | 1075 | 19.02 |
| 5G5 | 71 | 116 | 745 | 235 | 1167 | 17.57 |
| 5G7 | 98 | 96 | 1166 | 376 | 1736 | 11.88 |
| 5G9 | 123 | 146 | 1089 | 263 | 1621 | 18.26 |
| 5H1 | 50 | 43 | 940 | 266 | 1299 | 7.44 |
| 5H2 | 146 | 193 | 1510 | 381 | 2230 | 16.58 |
| 5H6 | 80 | 102 | 1384 | 353 | 1919 | 9.98 |
| 5H7 | 203 | 136 | 1384 | 331 | 2054 | 18.15 |
| 5H8 | 95 | 105 | 1551 | 466 | 2217 | 9.47 |
| 5H9 | 100 | 111 | 1388 | 343 | 1942 | 11.53 |

**Supplemental Table S14. HEI10 immunocytology data in Col, Ler and *rQTL* recombinants.** Nuclei were immunostained for the axis protein ASY1 to determine the meiotic stage. Total HEI10 immunofluorescent signal was measured from leptotene nuclei using ImageJ software analysis as described in methods. Col and Ler HEI10 intensity were not significantly different (Mann-Whitney-Wilcoxon test *P=*0.776).

| Genotype | 13E | Ler | Col | I6 |
| --- | --- | --- | --- | --- |
| *rQTL1* | Ler | Ler | Col | Col |
| *rQTL4* | Col | Ler | Col | Ler |
|  | 1.3 | 0.6 | 0.7 | 0.8 |
|  | 1.4 | 0.7 | 0.9 | 0.9 |
|  | 1.4 | 0.8 | 0.9 | 0.9 |
|  | 1.5 | 0.9 | 1 | 1 |
|  | 1.5 | 1.1 | 1.1 | 1 |
|  | 1.6 | 1.2 | 1.2 | 1.1 |
|  | 1.7 | 1.2 | 1.3 | 1.2 |
|  | 1.8 | 1.3 | 1.4 | 1.2 |
|  | 1.8 | 1.5 | 1.4 | 1.2 |
|  | 2 | 1.6 | 1.5 | 1.2 |
|  | 2 | 1.8 | 1.5 | 1.2 |
|  | 2.1 | 1.9 | 1.5 | 1.3 |
|  | 2.1 | 1.9 | 1.7 | 1.4 |
|  | 2.2 | 2.4 | 1.7 | 1.5 |
|  | 2.2 | 2.5 | 1.9 | 1.6 |
|  | 2.4 | 2.6 | 2 | 1.7 |
|  | 2.6 | 2.7 | 2.2 | 1.7 |
|  | 2.7 | 2.8 | 2.9 | 1.8 |
|  | 2.8 | 2.9 | 3.1 | 1.9 |
|  | 3.1 | 2.9 | 3.7 | 2.1 |
| Mean | 2.0 | 1.8 | 1.7 | 1.3 |
| StDev | 0.5 | 0.8 | 0.8 | 0.4 |

**Supplemental Table S15. HEI10 foci number data in Col, Ler and *rQTL* recombinants.** Nuclei were immunostained for the axis protein ASY1 to determine the meiotic stage. HEI10 recombination foci were scored at leptotene stage. Foci number were not significantly different between Col and Ler (Mann-Whitney-Wilcoxon test *P=*0.337).

| Genotype | Col | Ler | 13E | I6 |
| --- | --- | --- | --- | --- |
| *rQTL1* | Col | Ler | Ler | Col |
| *rQTL4* | Col | Ler | Col | Ler |
|  | 150 | 115 | 142 | 129 |
|  | 154 | 130 | 145 | 134 |
|  | 166 | 138 | 151 | 136 |
|  | 168 | 143 | 158 | 139 |
|  | 168 | 152 | 159 | 144 |
|  | 170 | 163 | 160 | 145 |
|  | 172 | 168 | 163 | 151 |
|  | 178 | 171 | 166 | 153 |
|  | 182 | 175 | 167 | 157 |
|  | 184 | 176 | 168 | 158 |
|  | 184 | 185 | 171 | 159 |
|  | 185 | 187 | 171 | 165 |
|  | 185 | 197 | 175 | 165 |
|  | 187 | 198 | 179 | 165 |
|  | 189 | 198 | 185 | 169 |
|  | 193 | 200 | 188 | 174 |
|  | 196 | 200 | 189 | 174 |
|  | 196 | 218 | 191 | 181 |
|  | 202 | 232 | 202 | 181 |
|  | 212 | 238 | 210 | 192 |
|  | 220 |  |  |  |
| Mean | 183 | 179 | 172 | 159 |
| StDev | 17.0 | 32.0 | 17.6 | 16.9 |

**Supplemental Table S16. *HEI10* qRT-PCR analysis.** The relative expression levels of *HEI10* were measured by quantitative PCR. The meiosis-specific gene *DMC1* was used as control for ΔCt calculations. ANOVA analysis showed no significant difference in expression between genotypes (*P*=0.127).

| Template | *HEI10* | St.Dev. |
| --- | --- | --- |
| Col | 0.250 | 0.428 |
| Col/Ler F_1_ | -0.444 | 0.131 |
| Ler | -0.066 | 0.278 |

**Supplemental Table S17.** **Col/Ler *rQTL* recombinant** ***420*×*hei10-2* fluorescent count data.** Four recombinant individuals were identified that were homozygous for *rQTL4^Ler^* and either homozygous for *rQTL1^Col^* or *rQTL1^Ler^* and that carried the *420* crossover reporter. These parent lines (55C, 47I, 27H and 54E) were then crossed to wild type (wt, Col) or to *hei10-2* (*hei10*) heterozygotes. Replicate progeny from these crosses were then grown, genotyped for *hei10*, and *420* crossover frequency measured. *420* genetic distance is calculated as cM = 100 × (1–(1−2(*N_G_*+*N_R_*)/*N_T_*)^1/2^), where *N_G_* is the number of green alone seeds, *N_R_* is the number of red alone seeds and *N_T_* is the total number of seeds analysed. To test for significant differences between wild type and *hei10/+* progeny from a given parent line (e.g. 55C), the number of recombinant and non-recombinant seed for each genotype class were used to construct 2×2 contingency tables and *X^2^* tests performed.

| *rQTL1* | *rQTL4* | Parent line | Cross | Green | Red | Both | None | Total | cM | *X^2^ P* |
| --- | --- | --- | --- | --- | --- | --- | --- | --- | --- | --- |
| Col | Ler | 55C | wt | 258 | 267 | 2013 | 467 | 3005 | 19.34 |  |
| Col | Ler | 55C | wt | 334 | 264 | 2078 | 462 | 3138 | 21.33 |  |
| Col | Ler | 55C | wt | 328 | 282 | 2034 | 459 | 3103 | 22.1 |  |
| Col | Ler | 55C | wt | 333 | 257 | 2090 | 487 | 3167 | 20.79 |  |
| Col | Ler | 55C | wt | 244 | 316 | 1966 | 451 | 2977 | 21.02 |  |
| Col | Ler | 55C | wt | 285 | 295 | 1921 | 452 | 2953 | 22.08 |  |
| Col | Ler | 55C | wt | 266 | 282 | 1915 | 438 | 2901 | 21.12 | n.d. |
| Col | Ler | 55C | *hei10* | 249 | 249 | 2054 | 543 | 3095 | 17.65 |  |
| Col | Ler | 55C | *hei10* | 228 | 228 | 1956 | 558 | 2970 | 16.76 |  |
| Col | Ler | 55C | *hei10* | 251 | 219 | 2089 | 580 | 3139 | 16.3 |  |
| Col | Ler | 55C | *hei10* | 246 | 233 | 2041 | 491 | 3011 | 17.43 |  |
| Col | Ler | 55C | *hei10* | 214 | 232 | 1863 | 473 | 2782 | 17.58 |  |
| Col | Ler | 55C | *hei10* | 200 | 258 | 1775 | 446 | 2679 | 18.88 | 9.97×10^-15^ |
| Col | Ler | 47I | wt | 283 | 269 | 1960 | 471 | 2983 | 20.63 |  |
| Col | Ler | 47I | wt | 240 | 254 | 1887 | 500 | 2881 | 18.94 | n.d. |
| Col | Ler | 47I | *hei10* | 184 | 189 | 1748 | 546 | 2667 | 15.13 |  |
| Col | Ler | 47I | *hei10* | 263 | 221 | 2041 | 494 | 3019 | 17.58 |  |
| Col | Ler | 47I | *hei10* | 224 | 182 | 2055 | 529 | 2990 | 14.65 |  |
| Col | Ler | 47I | *hei10* | 217 | 201 | 2018 | 532 | 2968 | 15.25 |  |
| Col | Ler | 47I | *hei10* | 164 | 205 | 1950 | 535 | 2854 | 13.89 |  |
| Col | Ler | 47I | *hei10* | 171 | 159 | 1570 | 498 | 2398 | 14.87 | 5.18 x10^-12^ |
| Ler | Ler | 27H | wt | 221 | 254 | 2108 | 539 | 3122 | 16.59 |  |
| Ler | Ler | 27H | wt | 305 | 247 | 2194 | 625 | 3371 | 17.99 |  |
| Ler | Ler | 27H | wt | 256 | 253 | 2104 | 590 | 3203 | 17.41 |  |
| Ler | Ler | 27H | wt | 283 | 246 | 2233 | 582 | 3344 | 17.32 |  |
| Ler | Ler | 27H | wt | 238 | 223 | 2103 | 545 | 3109 | 16.13 |  |
| Ler | Ler | 27H | wt | 252 | 236 | 2119 | 613 | 3220 | 16.52 |  |
| Ler | Ler | 27H | wt | 246 | 260 | 2129 | 542 | 3177 | 17.45 | n.d. |
| Ler | Ler | 27H | *hei10* | 232 | 193 | 2152 | 570 | 3147 | 14.57 |  |
| Ler | Ler | 27H | *hei10* | 187 | 201 | 2308 | 597 | 3293 | 12.57 |  |
| Ler | Ler | 27H | *hei10* | 213 | 238 | 2170 | 559 | 3180 | 15.36 |  |
| Ler | Ler | 27H | *hei10* | 217 | 193 | 2316 | 644 | 3370 | 13.01 | 2.77×10^-12^ |
| Ler | Ler | 54E | wt | 227 | 189 | 2164 | 637 | 3217 | 13.9 |  |
| Ler | Ler | 54E | wt | 266 | 231 | 2222 | 572 | 3291 | 16.46 |  |
| Ler | Ler | 54E | wt | 262 | 226 | 2214 | 584 | 3286 | 16.16 |  |
| Ler | Ler | 54E | wt | 218 | 241 | 2122 | 503 | 3084 | 16.19 |  |
| Ler | Ler | 54E | wt | 244 | 250 | 2166 | 592 | 3252 | 16.56 | n.d. |
| Ler | Ler | 54E | *hei10* | 222 | 169 | 2299 | 673 | 3363 | 12.39 |  |
| Ler | Ler | 54E | *hei10* | 193 | 178 | 2280 | 590 | 3241 | 12.19 |  |
| Ler | Ler | 54E | *hei10* | 161 | 165 | 2086 | 600 | 3012 | 11.48 |  |
| Ler | Ler | 54E | *hei10* | 160 | 185 | 2125 | 621 | 3091 | 11.87 | <2.2×10^-16^ |

**Supplemental Table S18. Col-*420*×ZMM crossover pathway mutant F_1_ fluorescent count data.** We obtained characterized loss of function alleles of ZMM crossover pathway mutants and crossed these with Col-*420*. Replicate measurements were made of mutant heterozygotes, which were compared to a control set of Col-*420* wild type (Supplemental Table S2). To test for significant differences, recombinant and non-recombinant count data for wild type and mutant heterozygotes were used to construct 2×2 contingency tables and *X^2^* tests performed.

| Genotype | Green | Red | Both | None | cM | *X^2^ P* |
| --- | --- | --- | --- | --- | --- | --- |
| *hei10/+* | 210 | 305 | 2573 | 685 | 14.74 |  |
| *hei10/+* | 172 | 162 | 1972 | 490 | 12.76 |  |
| *hei10/+* | 83 | 54 | 714 | 190 | 14.16 |  |
| *hei10/+* | 269 | 265 | 2717 | 806 | 14.17 |  |
| *hei10/+* | 257 | 321 | 2896 | 716 | 14.91 | 1.63×10^-38^ |
| *msh4-1/+* | 361 | 327 | 2555 | 615 | 19.79 |  |
| *msh4-1/+* | 261 | 215 | 2102 | 531 | 16.71 |  |
| *msh4-1/+* | 280 | 246 | 2073 | 536 | 18.49 |  |
| *msh4-1/+* | 208 | 210 | 1608 | 417 | 18.9 |  |
| *msh4-1/+* | 252 | 215 | 1826 | 412 | 19.09 |  |
| *msh4-1/+* | 288 | 267 | 2153 | 564 | 18.71 | 0.164 |
| *msh5-1/+* | 370 | 287 | 2318 | 581 | 20.6 |  |
| *msh5-1/+* | 239 | 216 | 1929 | 458 | 17.55 |  |
| *msh5-1/+* | 128 | 183 | 1269 | 277 | 18.45 |  |
| *msh5-1/+* | 225 | 237 | 1774 | 423 | 19.22 |  |
| *msh5-1/+* | 230 | 248 | 1956 | 496 | 17.92 | 0.476 |
| *shoc1/+* | 324 | 236 | 1990 | 457 | 20.78 |  |
| *shoc1/+* | 140 | 188 | 1281 | 311 | 18.86 |  |
| *shoc1/+* | 128 | 104 | 771 | 181 | 22.02 |  |
| *shoc1/+* | 102 | 125 | 895 | 216 | 18.72 |  |
| *shoc1/+* | 101 | 112 | 882 | 241 | 17.47 |  |
| *shoc1/+* | 214 | 242 | 1871 | 436 | 18.15 |  |
| *shoc1/+* | 157 | 155 | 1462 | 332 | 16.11 | 0.445 |
| *ptd1/+* | 347 | 305 | 2506 | 624 | 19.06 |  |
| *ptd1/+* | 294 | 266 | 2326 | 577 | 17.75 |  |
| *ptd1/+* | 275 | 263 | 1986 | 431 | 20.26 |  |
| *ptd1/+* | 341 | 312 | 2517 | 681 | 18.71 |  |
| *ptd1/+* | 350 | 363 | 2703 | 650 | 19.42 |  |
| *ptd1/+* | 340 | 268 | 2350 | 544 | 19.21 | 0.758 |

**Supplemental Table S19. Col-*420 HEI10* T_1_ fluorescent count data.** Genetic distance is calculated as cM = 100 × (1 – (1 − 2(*N_G_*+*N_R_*)/*N_T_*)^1/2^), where *N_G_* is the number of green alone seeds, *N_R_* is the number of red alone seeds and *N_T_* is the total number of seeds analysed. In some cases transgenic lines had a *trans* configuration of FTL T-DNAs, where genetic distance is calclulated as cM = 100 - (100 × (1 – (1 − 2(*N_G_*+*N_R_*)/*N_T_*)^1/2^)).

| Genotype | Green alone | Red alone | Both colors | No color | Total | cM |
| --- | --- | --- | --- | --- | --- | --- |
| HEI10 *Col::Col* | 210 | 236 | 1051 | 211 | 1708 | 30.88 |
| HEI10 *Col::Col* | 266 | 246 | 1161 | 224 | 1897 | 32.16 |
| HEI10 *Col::Col* | 193 | 154 | 903 | 240 | 1490 | 26.91 |
| HEI10 *Col::Col* | 285 | 283 | 1280 | 192 | 2040 | 33.43 |
| HEI10 *Col::Col* | 387 | 406 | 1805 | 355 | 2953 | 31.96 |
| HEI10 *Col::Col* | 201 | 262 | 1199 | 259 | 1921 | 28.03 |
| HEI10 *Col::Col* | 271 | 278 | 760 | 51 | 1360 | 43.89 |
| HEI10 *Col::Col* | 322 | 301 | 1890 | 405 | 2918 | 24.3 |
| HEI10 *Col::Col* | 96 | 104 | 527 | 99 | 826 | 28.19 |
| HEI10 *Col::Col* | 66 | 24 | 525 | 122 | 737 | 13.07 |
| HEI10 *Col::Col* | 346 | 397 | 939 | 69 | 1751 | 38.9 |
| HEI10 *Col::Col* | 271 | 288 | 1597 | 356 | 2512 | 25.51 |
| HEI10 *Col::Col* | 321 | 303 | 1796 | 437 | 2857 | 24.95 |
| HEI10 *Col::Col* | 120 | 136 | 813 | 183 | 1252 | 23.12 |
| HEI10 *Col::Col* | 174 | 202 | 1023 | 200 | 1599 | 27.22 |
| HEI10 *Col::Col* | 487 | 535 | 1929 | 288 | 3239 | 39.26 |
| HEI10 *Col::Col* | 332 | 401 | 2045 | 479 | 3257 | 25.85 |
| HEI10 *Col::Col* | 341 | 334 | 1503 | 289 | 2467 | 32.71 |
| HEI10 *Col::Col* | 341 | 342 | 1377 | 193 | 2253 | 37.25 |
| HEI10 *Col::Col* | 253 | 381 | 1411 | 179 | 2224 | 34.44 |
| HEI10 *Col::Col* | 393 | 387 | 1835 | 365 | 2980 | 30.97 |
| HEI10 *Col::Col* | 316 | 339 | 1860 | 406 | 2921 | 25.74 |
| HEI10 *Col::Col* | 375 | 420 | 1760 | 339 | 2894 | 32.87 |
| HEI10 *Col::Col* | 327 | 419 | 1637 | 277 | 2660 | 33.74 |
| HEI10 *Col::Col* | 337 | 314 | 1906 | 395 | 2952 | 25.24 |
| HEI10 *Col::Col* | 428 | 373 | 1225 | 186 | 2212 | 47.49 |
| HEI10 *Col::Col* | 200 | 257 | 1327 | 329 | 2113 | 24.67 |
| HEI10 *Col::Col* | 210 | 209 | 1018 | 191 | 1628 | 30.34 |
| HEI10 *Col::Col* | 334 | 358 | 1844 | 355 | 2891 | 27.8 |
| HEI10 *Col::Col* | 306 | 271 | 2025 | 485 | 3087 | 20.87 |
| HEI10 *Col::Col* | 162 | 185 | 1005 | 261 | 1613 | 24.52 |
| HEI10 *Col::Col* | 194 | 151 | 948 | 214 | 1507 | 26.37 |
| HEI10 *Col::Col* | 102 | 90 | 715 | 184 | 1091 | 19.5 |
| HEI10 *Col::Col* | 149 | 131 | 733 | 235 | 1248 | 25.75 |
| HEI10 *Col::Col* | 87 | 114 | 627 | 131 | 959 | 23.79 |
| HEI10 *Col::Col* | 23 | 139 | 201 | 98 | 461 | 45.49 |
| HEI10 *Col::Col* | 135 | 134 | 906 | 229 | 1404 | 21.46 |
| HEI10 *Col::Col* | 108 | 127 | 829 | 227 | 1291 | 20.25 |
| HEI10 *Col::Col* | 116 | 128 | 623 | 156 | 1023 | 27.68 |
| HEI10 *Col::Col* | 236 | 260 | 1196 | 285 | 1977 | 29.41 |
| HEI10 *Col::Col* | 319 | 335 | 1781 | 393 | 2828 | 26.69 |
| HEI10 *Col::Col* | 259 | 282 | 1517 | 314 | 2372 | 26.25 |
| HEI10 *Col::Col* | 307 | 311 | 1838 | 418 | 2874 | 24.51 |
| HEI10 *Col::Col* | 290 | 302 | 1907 | 459 | 2958 | 22.56 |
| HEI10 *Col::Col* | 241 | 225 | 1207 | 221 | 1894 | 28.73 |
| HEI10 *Col::Col* | 218 | 212 | 1353 | 291 | 2074 | 23.49 |
| HEI10 *Col::Col* | 268 | 250 | 1661 | 375 | 2554 | 22.91 |
| HEI10 *Col::Col* | 169 | 160 | 1237 | 267 | 1833 | 19.94 |
| HEI10 *Col::Col* | 173 | 169 | 369 | 13 | 724 | 23.51 |
| HEI10 *Col::Col* | 230 | 179 | 1108 | 264 | 1781 | 26.47 |
| HEI10 *Col::Col* | 110 | 194 | 1158 | 322 | 1784 | 18.81 |
| HEI10 *Col::Col* | 434 | 432 | 1845 | 347 | 3058 | 34.15 |
| HEI10 *Ler::Ler* | 296 | 284 | 1613 | 329 | 2522 | 26.51 |
| HEI10 *Ler::Ler* | 287 | 307 | 1921 | 499 | 3014 | 22.16 |
| HEI10 *Ler::Ler* | 186 | 162 | 1013 | 215 | 1576 | 25.28 |
| HEI10 *Ler::Ler* | 145 | 184 | 1184 | 262 | 1775 | 20.67 |
| HEI10 *Ler::Ler* | 148 | 143 | 669 | 153 | 1113 | 30.93 |
| HEI10 *Ler::Ler* | 238 | 224 | 1362 | 281 | 2105 | 25.1 |
| HEI10 *Ler::Ler* | 248 | 257 | 1279 | 231 | 2015 | 29.38 |
| HEI10 *Ler::Ler* | 187 | 187 | 1237 | 329 | 1940 | 21.61 |
| HEI10 *Ler::Ler* | 149 | 155 | 1071 | 279 | 1654 | 20.48 |
| HEI10 *Ler::Ler* | 201 | 193 | 1376 | 298 | 2068 | 21.33 |
| HEI10 *Ler::Ler* | 243 | 235 | 1923 | 428 | 2829 | 18.63 |
| HEI10 *Ler::Ler* | 226 | 211 | 1694 | 377 | 2508 | 19.28 |
| HEI10 *Ler::Ler* | 292 | 84 | 595 | 464 | 1435 | 31.01 |
| HEI10 *Ler::Ler* | 257 | 218 | 1235 | 295 | 2005 | 27.46 |
| HEI10 *Ler::Ler* | 122 | 111 | 770 | 212 | 1215 | 21.48 |
| HEI10 *Ler::Ler* | 153 | 118 | 954 | 278 | 1503 | 20.04 |
| HEI10 *Ler::Ler* | 125 | 133 | 842 | 239 | 1339 | 21.6 |
| HEI10 *Ler::Ler* | 324 | 414 | 1595 | 251 | 2584 | 34.52 |
| HEI10 *Ler::Ler* | 367 | 378 | 1671 | 314 | 2730 | 32.6 |
| HEI10 *Ler::Ler* | 130 | 128 | 575 | 99 | 932 | 33.19 |
| HEI10 *Ler::Ler* | 330 | 319 | 1868 | 410 | 2927 | 25.4 |
| HEI10 *Ler::Ler* | 110 | 112 | 559 | 111 | 892 | 29.13 |
| HEI10 *Ler::Ler* | 106 | 179 | 1040 | 283 | 1608 | 19.66 |
| HEI10 *Ler::Ler* | 48 | 37 | 321 | 83 | 489 | 19.23 |
| HEI10 *Ler::Ler* | 126 | 99 | 945 | 202 | 1372 | 18.02 |
| HEI10 *Ler::Ler* | 63 | 74 | 462 | 161 | 760 | 20.03 |
| HEI10 *Ler::Ler* | 156 | 149 | 1000 | 246 | 1551 | 22.11 |
| HEI10 *Ler::Ler* | 209 | 261 | 1782 | 464 | 2716 | 19.14 |
| HEI10 *Ler::Ler* | 470 | 421 | 1962 | 361 | 3214 | 33.25 |
| HEI10 *Ler::Ler* | 93 | 97 | 819 | 190 | 1199 | 17.35 |
| HEI10 *Ler::Ler* | 362 | 371 | 1532 | 275 | 2540 | 34.97 |
| HEI10 *Ler::Ler* | 297 | 356 | 1916 | 419 | 2988 | 24.97 |
| HEI10 *Ler::Ler* | 271 | 287 | 1869 | 457 | 2884 | 21.7 |
| HEI10 *Ler::Ler* | 253 | 290 | 2045 | 464 | 3052 | 19.74 |
| HEI10 *Ler::Ler* | 278 | 346 | 1916 | 420 | 2960 | 23.95 |
| HEI10 *Ler::Ler* | 29 | 23 | 105 | 27 | 184 | 34.06 |
| HEI10 *Ler::Ler* | 156 | 136 | 907 | 207 | 1406 | 23.54 |
| HEI10 *Ler::Ler* | 43 | 57 | 397 | 111 | 608 | 18.08 |
| HEI10 *Ler::Ler* | 334 | 330 | 1331 | 184 | 2179 | 37.51 |
| HEI10 *Ler::Ler* | 291 | 323 | 2010 | 432 | 3056 | 22.66 |
| HEI10 *Ler::Ler* | 327 | 284 | 1796 | 379 | 2786 | 25.07 |
| HEI10 *Ler::Ler* | 321 | 331 | 1746 | 402 | 2800 | 26.91 |
| HEI10 *Ler::Ler* | 540 | 511 | 1150 | 33 | 2234 | 24.31 |
| HEI10 *Ler::Ler* | 213 | 211 | 1586 | 420 | 2430 | 19.31 |
| HEI10 *Ler::Ler* | 208 | 206 | 1253 | 346 | 2013 | 23.27 |
| HEI10 *Ler::Ler* | 162 | 191 | 1072 | 246 | 1671 | 24.01 |
| HEI10 *Ler::Ler* | 92 | 97 | 627 | 136 | 952 | 22.35 |
| HEI10 *Ler::Ler* | 384 | 386 | 1497 | 258 | 2525 | 37.54 |
| HEI10 *Ler::Ler* | 225 | 221 | 1085 | 191 | 1722 | 30.57 |
| HEI10 *Ler::Ler* | 202 | 192 | 1355 | 322 | 2071 | 21.29 |
| HEI10 *Ler::Ler* | 392 | 346 | 1774 | 309 | 2821 | 30.95 |
| HEI10 *Ler::Ler* | 379 | 295 | 1167 | 247 | 2088 | 40.47 |
| HEI10 *Ler::Ler* | 243 | 246 | 1808 | 464 | 2761 | 19.64 |
| HEI10 *Ler::Ler* | 289 | 347 | 1712 | 331 | 2679 | 27.53 |
| HEI10 *Ler::Ler* | 252 | 305 | 2051 | 506 | 3114 | 19.86 |
| HEI10 *Ler::Ler* | 415 | 449 | 1722 | 253 | 2839 | 37.44 |
| HEI10 *Ler::Ler* | 269 | 320 | 1939 | 401 | 2929 | 22.68 |
| Empty | 291 | 235 | 1820 | 500 | 2846 | 20.6 |
| Empty | 201 | 227 | 1820 | 456 | 2704 | 17.33 |
| Empty | 168 | 183 | 2009 | 381 | 2741 | 13.75 |
| Empty | 220 | 271 | 1724 | 476 | 2691 | 20.31 |
| Empty | 236 | 270 | 1754 | 514 | 2774 | 20.3 |
| Empty | 283 | 215 | 1817 | 544 | 2859 | 19.28 |
| Empty | 274 | 251 | 2007 | 527 | 3059 | 18.96 |
| Empty | 211 | 235 | 1616 | 388 | 2450 | 20.26 |
| Empty | 200 | 289 | 2129 | 490 | 3108 | 17.22 |
| Empty | 208 | 247 | 1982 | 533 | 2970 | 16.72 |
| *HEI10 Ler::Col* | 300 | 387 | 1202 | 261 | 2150 | 39.92 |
| *HEI10 Ler::Col* | 173 | 256 | 1374 | 409 | 2212 | 21.76 |
| *HEI10 Ler::Col* | 247 | 146 | 969 | 257 | 1619 | 28.27 |
| *HEI10 Ler::Col* | 319 | 324 | 1313 | 275 | 2231 | 34.92 |
| *HEI10 Ler::Col* | 151 | 194 | 657 | 142 | 1144 | 37 |
| *HEI10 Ler::Col* | 64 | 67 | 246 | 52 | 429 | 37.61 |
| *HEI10 Ler::Col* | 119 | 102 | 343 | 33 | 597 | 49.05 |
| *HEI10 Ler::Col* | 272 | 283 | 1268 | 449 | 2272 | 28.48 |
| *HEI10 Ler::Col* | 243 | 244 | 972 | 209 | 1668 | 35.5 |
| *HEI10 Ler::Col* | 234 | 214 | 1027 | 215 | 1690 | 31.46 |
| *HEI10 Ler::Col* | 84 | 81 | 457 | 167 | 789 | 23.73 |
| *HEI10 Ler::Col* | 195 | 251 | 1342 | 354 | 2142 | 23.61 |
| *HEI10 Ler::Col* | 248 | 233 | 1681 | 411 | 2573 | 20.87 |
| *HEI10 Ler::Col* | 48 | 47 | 289 | 104 | 488 | 21.86 |
| *HEI10 Ler::Col* | 373 | 313 | 1713 | 434 | 2833 | 28.19 |
| *HEI10 Ler::Col* | 186 | 183 | 896 | 176 | 1441 | 30.15 |
| *HEI10 Ler::Col* | 80 | 117 | 494 | 151 | 842 | 27.06 |
| *HEI10 Ler::Col* | 228 | 239 | 1413 | 315 | 2195 | 24.21 |
| *HEI10 Ler::Col* | 308 | 336 | 1775 | 383 | 2802 | 26.49 |
| *HEI10 Ler::Col* | 373 | 360 | 1473 | 249 | 2455 | 36.53 |
| *HEI10 Ler::Col* | 253 | 301 | 1158 | 235 | 1947 | 34.36 |
| *HEI10 Ler::Col* | 91 | 62 | 491 | 132 | 776 | 22.18 |
| *HEI10 Ler::Col* | 238 | 267 | 1565 | 437 | 2507 | 22.73 |
| *HEI10 Ler::Col* | 241 | 289 | 1506 | 419 | 2455 | 24.62 |
| *HEI10 Ler::Col* | 72 | 84 | 451 | 85 | 692 | 25.9 |
| *HEI10 Col::Ler* | 205 | 254 | 1564 | 369 | 2392 | 21.5 |
| *HEI10 Col::Ler* | 135 | 155 | 692 | 216 | 1198 | 28.18 |
| *HEI10 Col::Ler* | 256 | 290 | 1321 | 292 | 2159 | 29.7 |
| *HEI10 Col::Ler* | 41 | 52 | 2023 | 730 | 2846 | 3.32 |
| *HEI10 Col::Ler* | 331 | 303 | 1637 | 364 | 2635 | 27.97 |
| *HEI10 Col::Ler* | 287 | 301 | 1883 | 423 | 2894 | 22.95 |
| *HEI10 Col::Ler* | 171 | 256 | 1446 | 315 | 2188 | 21.92 |
| *HEI10 Col::Ler* | 330 | 284 | 1667 | 409 | 2690 | 26.28 |
| *HEI10 Col::Ler* | 272 | 281 | 1780 | 458 | 2791 | 22.3 |
| *HEI10 Col::Ler* | 280 | 268 | 1529 | 437 | 2514 | 24.9 |
| *HEI10 Col::Ler* | 125 | 153 | 767 | 232 | 1277 | 24.86 |
| *HEI10 Col::Ler* | 283 | 302 | 2094 | 507 | 3186 | 20.45 |
| *HEI10 Col::Ler* | 58 | 65 | 383 | 69 | 575 | 24.36 |
| *HEI10 Col::Ler* | 236 | 274 | 1575 | 352 | 2437 | 23.75 |
| *HEI10 Col::Ler* | 236 | 274 | 1575 | 352 | 2437 | 23.75 |
| *HEI10 Col::Ler* | 200 | 226 | 1577 | 400 | 2403 | 19.66 |
| *HEI10 Col::Ler* | 127 | 179 | 1130 | 280 | 1716 | 19.79 |
| *HEI10 Col::Ler* | 113 | 142 | 1002 | 246 | 1503 | 18.72 |
| *HEI10 Col::Ler* | 111 | 139 | 729 | 160 | 1139 | 25.1 |
| *HEI10 Col::Ler* | 276 | 265 | 1539 | 375 | 2455 | 25.22 |
| *HEI10 Col::Ler* | 106 | 110 | 496 | 105 | 817 | 31.35 |
| *HEI10 Col::Ler* | 282 | 283 | 1808 | 497 | 2870 | 22.14 |
| *HEI10 Col::Ler* | 124 | 138 | 601 | 167 | 1030 | 29.91 |
| *HEI10 Col::Ler* | 370 | 362 | 1609 | 402 | 2743 | 31.72 |
| *HEI10 Col::Ler* | 226 | 247 | 1522 | 393 | 2388 | 22.29 |
| *HEI10 Col::Ler* | 227 | 313 | 1883 | 479 | 2902 | 20.76 |
| *HEI10 Col::Ler* | 258 | 258 | 1730 | 337 | 2583 | 22.51 |
| *HEI10 Col::Ler* | 284 | 285 | 1605 | 450 | 2624 | 24.75 |
| *HEI10 Col::Ler* | 248 | 271 | 1655 | 518 | 2692 | 21.62 |
| *HEI10 Col::Ler* | 284 | 329 | 1818 | 456 | 2887 | 24.15 |
| *HEI10 Col::Ler* | 307 | 295 | 2011 | 490 | 3103 | 21.77 |
| *HEI10 Col::Ler* | 250 | 265 | 1295 | 325 | 2135 | 28.06 |
| *HEI10 Col::Ler* | 221 | 325 | 1250 | 339 | 2135 | 30.11 |
| *HEI10 Col::Ler* | 123 | 166 | 1037 | 289 | 1615 | 19.87 |
| *HEI10 Col::Ler* | 265 | 269 | 1137 | 284 | 1955 | 32.64 |
| *HEI10 Col::Ler* | 255 | 246 | 1659 | 467 | 2627 | 21.35 |
| *HEI10 Col::Ler* | 268 | 272 | 1690 | 433 | 2663 | 22.9 |
| *HEI10 Col::Ler* | 271 | 263 | 1341 | 462 | 2337 | 26.31 |
| *HEI10 Col::Ler* | 245 | 286 | 1250 | 201 | 1982 | 31.87 |
| *HEI10 Col::Ler* | 72 | 77 | 376 | 113 | 638 | 27 |
| *HEI10 Col::Ler* | 269 | 232 | 1838 | 464 | 2803 | 19.84 |
| *HEI10 Col::Ler* | 243 | 260 | 1367 | 349 | 2219 | 26.06 |
| *HEI10 Col::Ler* | 335 | 358 | 1803 | 346 | 2842 | 28.42 |
| *HEI10 Col::Ler* | 316 | 265 | 1871 | 447 | 2899 | 22.59 |
| *HEI10 Col::Ler* | 347 | 325 | 1452 | 309 | 2433 | 33.1 |
| *HEI10 Col::Ler* | 215 | 212 | 1243 | 380 | 2050 | 23.62 |
| *HEI10 Col::Ler* | 318 | 295 | 1516 | 324 | 2453 | 29.27 |
| *HEI10 Col::Ler* | 202 | 213 | 915 | 184 | 1514 | 32.79 |
| *HEI10 Col::Ler* | 317 | 243 | 1450 | 313 | 2323 | 28.04 |
| *HEI10 Col::Ler* | 342 | 370 | 1459 | 323 | 2494 | 34.5 |
| *HEI10 Col::Ler* | 239 | 293 | 1383 | 251 | 2166 | 28.67 |
| *HEI10 Col::Ler* | 288 | 290 | 1724 | 394 | 2696 | 24.42 |
| *HEI10 Col::Ler* | 97 | 222 | 841 | 258 | 1418 | 25.83 |
| *HEI10 Col::Ler* | 259 | 263 | 1664 | 402 | 2588 | 22.76 |
| *HEI10 Col::Ler* | 245 | 252 | 1468 | 411 | 2376 | 23.73 |

**Supplemental Table S20. *HEI10* T_1_ qRT-PCR analysis.** The relative expression levels of *HEI10* were measured by quantitative RT-PCR. The meiosis-specific gene *DMC1* was used as control for ΔCt calculations. Data for reference Col-*420* lines was used to calculate ΔΔCt and expression (fold change) values.

|  |  | *HEI10* | |
| --- | --- | --- | --- |
| Individual | Crossover (cM) | ΔΔCt | Expression (FC) |
| *HEI10^Ler^*-25 | 18.02 | -1.997 | 3.991 |
| *HEI10^Col^*-30 | 20.87 | -2.020 | 4.056 |
| *HEI10^Ler^*-10 | 21.33 | -1.803 | 3.490 |
| *HEI10^Col^*-44 | 22.56 | -1.673 | 3.190 |
| *HEI10^Ler^*-40 | 22.66 | -2.270 | 4.823 |
| *HEI10^Col^*-47 | 22.91 | -1.790 | 3.458 |
| *HEI10^Col^*-46 | 23.49 | -1.740 | 3.340 |
| *HEI10^Col^*-43 | 24.51 | -2.213 | 4.637 |
| *HEI10^Col^*-27 | 24.67 | -0.920 | 1.892 |
| *HEI10^Col^*-13 | 24.95 | -1.747 | 3.356 |
| *HEI10^Ler^*-3 | 25.28 | -2.193 | 4.574 |
| *HEI10^Col^*-42 | 26.25 | -1.743 | 3.348 |
| *HEI10^Col^*-50 | 26.47 | -2.217 | 4.648 |
| *HEI10^Col^*-29 | 27.80 | -2.000 | 4.000 |
| *HEI10^Col^*-6 | 28.03 | -1.910 | 3.758 |
| *HEI10^Col^*-9 | 28.19 | -1.910 | 3.758 |
| *HEI10^Col^*-45 | 28.73 | -2.980 | 7.890 |
| *HEI10^Col^*-28 | 30.34 | -2.470 | 5.540 |
| *HEI10^Ler^*-49 | 30.57 | -2.750 | 6.727 |
| *HEI10^Ler^*-5 | 30.93 | -2.873 | 7.328 |
| *HEI10^Ler^*-51 | 30.95 | -2.853 | 7.227 |
| *HEI10^Col^*-5 | 31.96 | -2.253 | 4.768 |
| *HEI10^Col^*-18 | 32.71 | -0.990 | 1.986 |
| *HEI10^Ler^*-29 | 33.25 | -3.243 | 9.470 |
| *HEI10^Ler^*-31 | 34.97 | -2.587 | 6.007 |
| *HEI10^Col^*-19 | 37.25 | -2.813 | 7.029 |
| *HEI10^Col^*-11 | 38.90 | -1.643 | 3.124 |
| *HEI10^Col^*-7 | 43.89 | -3.760 | 13.548 |
| *HEI10^Col^*-26 | 47.49 | -3.743 | 13.392 |

**Supplemental Table S21.** **HEI10 immunocytology data in Col and *HEI10^Col^* T_3_ line C2-6.** Nuclei were immunostained for the axis protein ASY1 to determine the meiotic stage. The total immunofluorescence signal of HEI10 was measured from leptotene nuclei using ImageJ software analysis as described in the Methods. HEI10 signal was normalised to the signal of an adjacent fluorescent microsphere bead. The plants analysed were T_3_ progeny of line C2-6 (Supplemental Table S24). The *HEI10****^Col^*** line showed significantly greater intensity compared with Col, with (Mann-Whitney-Wilcoxon test, *P*=3.90×10^-4^) and without (Mann-Whitney-Wilcoxon test, *P*=4.69×10^-4^) fluorescent bead normalization.

| Meiocyte | Col intensity | Col bead  intensity | Col relative  intensity | *HEI10-Col* C2 T_3_  intensity | *HEI10-Col* C2 T_3_  bead intensity | *HEI10-Col* C2 T_3_  relative intensity |
| --- | --- | --- | --- | --- | --- | --- |
| 1 | 1.3 | 1 | 1.3 | 1.3 | 1 | 1.3 |
| 2 | 1.6 | 1 | 1.6 | 1.6 | 1 | 1.6 |
| 3 | 1.6 | 1 | 1.6 | 1.6 | 1 | 1.6 |
| 4 | 1.4 | 0.9 | 1.6 | 1.7 | 1 | 1.7 |
| 5 | 1.6 | 1 | 1.6 | 1.8 | 1 | 1.8 |
| 6 | 1.6 | 1 | 1.6 | 1.7 | 0.9 | 1.9 |
| 7 | 1.6 | 1 | 1.6 | 2 | 1 | 2 |
| 8 | 1.5 | 0.9 | 1.7 | 2.1 | 1 | 2.1 |
| 9 | 1.8 | 1 | 1.8 | 2 | 0.9 | 2.2 |
| 10 | 1.6 | 0.9 | 1.8 | 2 | 0.9 | 2.2 |
| 11 | 1.6 | 0.9 | 1.8 | 2.3 | 1 | 2.3 |
| 12 | 1.8 | 1 | 1.8 | 2.6 | 1.1 | 2.4 |
| 13 | 1.9 | 1 | 1.9 | 2.8 | 1.1 | 2.5 |
| 14 | 1.7 | 0.9 | 1.9 | 2.5 | 1 | 2.5 |
| 15 | 1.9 | 1 | 1.9 | 2.5 | 1 | 2.5 |
| 16 | 1.7 | 0.9 | 1.9 | 2.6 | 1 | 2.6 |
| 17 | 1.9 | 1 | 1.9 | 2.7 | 1 | 2.7 |
| 18 | 2 | 1 | 2 | 2.8 | 1 | 2.8 |
| 19 | 1.9 | 0.9 | 2.1 | 2.9 | 1 | 2.9 |
| 20 | 1.9 | 0.9 | 2.1 | 3 | 1 | 3 |
| 21 | 2.1 | 1 | 2.1 | 2.7 | 0.9 | 3 |
| 22 | 2 | 0.9 | 2.2 | 3 | 1 | 3 |
| 23 | 2.2 | 1 | 2.2 | 3.1 | 1 | 3.1 |
| 24 | 2.1 | 0.9 | 2.3 | 3.1 | 1 | 3.1 |
| 25 | 2.1 | 0.9 | 2.3 | 2.9 | 0.9 | 3.2 |
| 26 | 2.1 | 0.9 | 2.3 | 3.2 | 1 | 3.2 |
| 27 | 2.2 | 0.9 | 2.4 | 3.3 | 1 | 3.3 |
| 28 | 2.2 | 0.9 | 2.4 | 3.4 | 1 | 3.4 |
| 29 | 2.7 | 1 | 2.7 | 3.5 | 1 | 3.5 |
| 30 | 2.7 | 1 | 2.7 | 3.6 | 1 | 3.6 |
| 31 | 2.5 | 0.9 | 2.8 | 3.2 | 0.9 | 3.6 |
| 32 | 2.8 | 1 | 2.8 | 3.7 | 1 | 3.7 |
| 33 | 2.9 | 1 | 2.9 | 3.8 | 1 | 3.8 |
| 34 | 2.9 | 1 | 2.9 | 3.5 | 0.9 | 3.9 |
| 35 | 2.6 | 0.9 | 2.9 | 3.9 | 1 | 3.9 |
| 36 | 2.5 | 0.9 | 3 | 3.7 | 0.9 | 4.1 |
| 37 | 2.9 | 0.9 | 3 | 3.3 | 0.8 | 4.1 |
| 38 | 2.8 | 0.9 | 3.1 | 3.9 | 0.9 | 4.3 |
| 39 | 3.3 | 1 | 3.3 | 4.5 | 1 | 4.5 |
| 40 | 3.6 | 1 | 3.6 | 3.7 | 0.8 | 4.6 |
| 41 | 3.2 | 0.9 | 3.6 | 4.6 | 1 | 4.6 |
| 42 | 3.2 | 0.9 | 3.6 | 1.9 | 0.4 | 4.8 |
| 43 | 3.5 | 0.9 | 3.9 | 2.9 | 0.6 | 4.8 |
| 44 | 4 | 0.9 | 4.4 | 4.2 | 0.8 | 5.3 |
| 45 | 4.1 | 0.9 | 4.6 | 4.4 | 0.6 | 7.3 |
| Mean | 2.3 | 0.9 | 2.4 | 2.9 | 0.9 | 3.2 |
| StDev | 0.7 | 0.0 | 0.8 | 0.8 | 0.1 | 1.2 |

**Supplemental Table S22.** **HEI10 foci number data in Col and *HEI10^Col^* T_3_ line C2-6.** Nuclei were immunostained for the axis protein ASY1 to determine the meiotic stage. HEI10 recombination foci were scored at leptotene stage for all nuclei. The plants analysed were T_3_ progeny of line C2-6 (Supplemental Table S24).

| Meiocyte | Col | *HEI10^Col^* T_3_ |
| --- | --- | --- |
| 1 | 150 | 162 |
| 2 | 154 | 143 |
| 3 | 166 | 146 |
| 4 | 168 | 146 |
| 5 | 168 | 153 |
| 6 | 170 | 163 |
| 7 | 172 | 174 |
| 8 | 178 | 174 |
| 9 | 182 | 174 |
| 10 | 184 | 174 |
| 11 | 184 | 177 |
| 12 | 185 | 181 |
| 13 | 185 | 186 |
| 14 | 187 | 188 |
| 15 | 189 | 191 |
| 16 | 193 | 194 |
| 17 | 196 | 195 |
| 18 | 196 | 204 |
| 19 | 202 | 205 |
| 20 | 212 | 206 |
| 21 | 220 | 214 |
| Mean | 183 | 179 |
| StDev | 17 | 20 |

**Supplemental Table S23. MLH1 foci in wild type (Col) and *HEI10^Col^*.** To test for significant differences between genotypes, Mann-Whitney-Wilcoxon tests (MWW) were used. A significant difference was observed between Col and *HEI10^Col^* C2 T_3_ (MWW *P*=4.83×10^-8^). The plants analysed were T_3_ progeny of line C2-6 (Supplemental Table S24).

| Meiocyte | Col | *HEI10^Col^* C2 T_3_ |
| --- | --- | --- |
| 1 | 6 | 10 |
| 2 | 7 | 11 |
| 3 | 7 | 11 |
| 4 | 8 | 12 |
| 5 | 8 | 12 |
| 6 | 8 | 13 |
| 7 | 8 | 14 |
| 8 | 8 | 14 |
| 9 | 9 | 14 |
| 10 | 9 | 15 |
| 11 | 9 | 15 |
| 12 | 9 | 15 |
| 13 | 10 | 15 |
| 14 | 10 | 16 |
| 15 | 10 | 16 |
| 16 | 10 | 16 |
| 17 | 10 | 16 |
| 18 | 11 | 17 |
| 19 | 11 | 17 |
| 20 | 12 | 17 |
| 21 | 15 | 17 |
| 22 |  | 17 |
| 23 |  | 17 |
| 24 |  | 18 |
| 25 |  | 18 |
| 26 |  | 18 |
| 27 |  | 19 |
| Mean | 9.3 | 14.4 |
| StDev | 1.9 | 2.1 |

**Supplemental Table S24.** ***420* fluorescent count data from *HEI10^Col^* transformant lines used for genotyping by sequencing.** The *420/++* HEI10Col C2 T_1_ line was self-fertilized and T_2_ progeny measured for recombination rate. The T_2_ lines C2-3 (red) and C2-6 (blue) were crossed to Ler to generate the C2_Ler_3 (red) and C2_Ler_6 T_2_/Ler (blue) F_1_ lines, respectively. The C2_Ler_6_3 T_2_/Ler F_1_ line (green) was used as the parent for the F_2_ population analysed by genotyping by sequencing. As a control, Col-420 x Ler F_1_ plants were scored, and line #1 (green) was used as the parent for the control F_2_ population analysed by genotyping by sequencing (Choi et al. 2016). Genetic distance is calculated as cM = 100 × (1 – (1 − 2(*N_G_*+*N_R_*)/*N_T_*)^1/2^), where *N_G_* is the number of green alone seeds, *N_R_* is the number of red alone seeds and *N_T_* is the total number of seeds analysed.

| Generation | Line | Green alone | Red alone | Both colours | No colour | Total | cM |
| --- | --- | --- | --- | --- | --- | --- | --- |
| T_1_ | C2 | 327 | 419 | 1637 | 277 | 2660 | 33.74 |
| T_2_ | C2-1 | 323 | 308 | 1125 | 186 | 1942 | 40.83 |
| T_2_ | C2-2 | 401 | 391 | 1687 | 348 | 2827 | 33.69 |
| T_2_ | C2-3 | 448 | 470 | 1546 | 240 | 2704 | 43.34 |
| T_2_ | C2-6 | 496 | 448 | 1604 | 239 | 2787 | 43.2 |
| T_2_ | C2-7 | 388 | 435 | 1699 | 297 | 2819 | 35.49 |
| T_2_ | C2-8 | 325 | 357 | 1308 | 247 | 2237 | 37.53 |
| T_2_ | C2-9 | 576 | 611 | 1535 | 121 | 2843 | 59.38 |
| T_2_/Ler F_1_ | C2_Ler_3_2 | 405 | 429 | 1777 | 271 | 2882 | 35.1 |
| T_2_/Ler F_1_ | C2_Ler_3_4 | 374 | 436 | 1638 | 258 | 2706 | 36.65 |
| T_2_/Ler F_1_ | C2_Ler_3_5 | 324 | 440 | 1576 | 238 | 2578 | 36.18 |
| T_2_/Ler F_1_ | C2_Ler_6_1 | 394 | 404 | 1543 | 232 | 2573 | 38.38 |
| T_2_/Ler F_1_ | C2_Ler_6_2 | 397 | 389 | 1532 | 258 | 2576 | 37.57 |
| T_2_/Ler F_1_ | C2_Ler_6_3 | 377 | 377 | 1360 | 222 | 2336 | 40.46 |
| T_2_/Ler F_1_ | C2_Ler_6_4 | 411 | 473 | 1701 | 296 | 2881 | 37.85 |
| T_2_/Ler F_1_ | C2_Ler_6_5 | 412 | 445 | 1576 | 326 | 2759 | 38.46 |
|  |  |  |  |  |  |  |  |
| Col-*420*/Ler F_1_ | #1 | 167 | 186 | 1803 | 486 | 2642 | 14.40 |
| Col-*420*/Ler F_1_ | #2 | 171 | 209 | 2116 | 594 | 3090 | 13.16 |
| Col-*420*/Ler F_1_ | #3 | 190 | 182 | 1982 | 503 | 2857 | 14.00 |
| Col-*420*/Ler F_1_ | #4 | 250 | 269 | 2386 | 597 | 3502 | 16.12 |
| Col-*420*/Ler F_1_ | #5 | 177 | 204 | 1891 | 506 | 2778 | 14.81 |

**Supplemental Table S25.** **Total aligning read pairs for barcoded libraries generated from wild type and *HEI10^Col^* F_2_ individuals used for genotyping-by-sequencing.** The L lane indicates lane number. The barcode numbers correspond to published adapter sequences (Rowan et al. 2015). The wild type libraries are as reported in (Choi et al. 2016). Fastq sequencing files are available from ArrayExpress accessions E-MTAB-4657 (wild type, (Choi et al. 2016)) and E-MTAB-4967 (*HEI10*).

| Barcode | Genotype | L | Matching reads | Genotype | L | Matching reads | Genotype | L | Matching reads | Genotype | L | Matching reads |
| --- | --- | --- | --- | --- | --- | --- | --- | --- | --- | --- | --- | --- |
| 1 | Wild type | 1 | 3405732 | Wild type | 2 | 2788922 | *HEI10* | 1 | 397177 | *HEI10* | 2 | 3006553 |
| 2 | Wild type | 1 | 3371021 | Wild type | 2 | 326024 | *HEI10* | 1 | 410222 | *HEI10* | 2 | 2144641 |
| 3 | Wild type | 1 | 2705685 | Wild type | 2 | 662598 | *HEI10* | 1 | 588544 | *HEI10* | 2 | 640006 |
| 4 | Wild type | 1 | 4181386 | Wild type | 2 | 862832 | *HEI10* | 1 | 828878 | *HEI10* | 2 | 2497316 |
| 5 | Wild type | 1 | 174344 | Wild type | 2 | 2400130 | *HEI10* | 1 | 736550 | *HEI10* | 2 | 2902014 |
| 6 | Wild type | 1 | 6314291 | Wild type | 2 | 495548 | *HEI10* | 1 | 519731 | *HEI10* | 2 | 798667 |
| 7 | Wild type | 1 | 5967854 | Wild type | 2 | 3470182 | *HEI10* | 1 | 385289 | *HEI10* | 2 | 449830 |
| 8 | Wild type | 1 | 2812962 | Wild type | 2 | 2745197 | *HEI10* | 1 | 1211807 | *HEI10* | 2 | 520820 |
| 9 | Wild type | 1 | 2482679 | Wild type | 2 | 2716911 | *HEI10* | 1 | 5928323 | *HEI10* | 2 | 6226377 |
| 10 | Wild type | 1 | 3876170 | Wild type | 2 | 2675526 | *HEI10* | 1 | 1105832 | *HEI10* | 2 | 1786860 |
| 11 | Wild type | 1 | 952389 | Wild type | 2 | 2771221 | *HEI10* | 1 | 575292 | *HEI10* | 2 | 2195579 |
| 12 | Wild type | 1 | 2336517 | Wild type | 2 | 1363971 | *HEI10* | 1 | 2652413 | *HEI10* | 2 | 414080 |
| 13 | Wild type | 1 | 2845367 | Wild type | 2 | 3212926 | *HEI10* | 1 | 657400 | *HEI10* | 2 | 614024 |
| 14 | Wild type | 1 | 2187808 | Wild type | 2 | 1893515 | *HEI10* | 1 | 2485696 | *HEI10* | 2 | 500549 |
| 15 | Wild type | 1 | 1406459 | Wild type | 2 | 5746504 | *HEI10* | 1 | 2377664 | *HEI10* | 2 | 2796635 |
| 16 | Wild type | 1 | 786891 | Wild type | 2 | 3949253 | *HEI10* | 1 | 1110201 | *HEI10* | 2 | 395778 |
| 17 | Wild type | 1 | 4084768 | Wild type | 2 | 6207252 | *HEI10* | 1 | 2047706 | *HEI10* | 2 | 5672678 |
| 18 | Wild type | 1 | 1870912 | Wild type | 2 | 2424259 | *HEI10* | 1 | 506800 | *HEI10* | 2 | 1848302 |
| 19 | Wild type | 1 | 3020860 | Wild type | 2 | 3031894 | *HEI10* | 1 | 5409471 | *HEI10* | 2 | 915746 |
| 20 | Wild type | 1 | 1661192 | Wild type | 2 | 2075522 | *HEI10* | 1 | 2392458 | *HEI10* | 2 | 1274154 |
| 21 | Wild type | 1 | 1276964 | Wild type | 2 | 1513680 | *HEI10* | 1 | 2289441 | *HEI10* | 2 | 2429500 |
| 22 | Wild type | 1 | 2846864 | Wild type | 2 | 1770320 | *HEI10* | 1 | 1997707 | *HEI10* | 2 | 930482 |
| 23 | Wild type | 1 | 3377644 | Wild type | 2 | 387992 | *HEI10* | 1 | 968263 | *HEI10* | 2 | 633887 |
| 24 | Wild type | 1 | 2546437 | Wild type | 2 | 705596 | *HEI10* | 1 | 2007647 | *HEI10* | 2 | 3541778 |
| 25 | Wild type | 1 | 1272766 | Wild type | 2 | 6690505 | *HEI10* | 1 | 1213866 | *HEI10* | 2 | 6529556 |
| 26 | Wild type | 1 | 741724 | Wild type | 2 | 4674228 | *HEI10* | 1 | 2225573 | *HEI10* | 2 | 992967 |
| 27 | Wild type | 1 | 1114050 | Wild type | 2 | 816999 | *HEI10* | 1 | 640164 | *HEI10* | 2 | 3184782 |
| 28 | Wild type | 1 | 1856989 | Wild type | 1 | 1388157 | *HEI10* | 1 | 1758346 | *HEI10* | 2 | 908562 |
| 29 | Wild type | 1 | 1468127 | Wild type | 2 | 813849 | *HEI10* | 1 | 915187 | *HEI10* | 2 | 831826 |
| 30 | Wild type | 1 | 951016 | Wild type | 2 | 2359825 | *HEI10* | 1 | 2397010 | *HEI10* | 2 | 641726 |
| 31 | Wild type | 1 | 2163079 | Wild type | 2 | 1448518 | *HEI10* | 1 | 1420255 | *HEI10* | 2 | 1172413 |
| 32 | Wild type | 1 | 1104164 | Wild type | 2 | 2868625 | *HEI10* | 1 | 1327389 | *HEI10* | 2 | 830603 |
| 33 | Wild type | 1 | 2022214 | Wild type | 2 | 3491837 | *HEI10* | 1 | 1505374 | *HEI10* | 2 | 3962428 |
| 34 | Wild type | 1 | 2813139 | Wild type | 2 | 6288025 | *HEI10* | 1 | 1140161 | *HEI10* | 2 | 3850885 |
| 35 | Wild type | 1 | 530724 | Wild type | 2 | 886250 | *HEI10* | 1 | 8399516 | *HEI10* | 2 | 1748872 |
| 36 | Wild type | 1 | 2465572 | Wild type | 2 | 1318385 | *HEI10* | 1 | 2344034 | *HEI10* | 2 | 983363 |
| 37 | Wild type | 1 | 1802453 | Wild type | 2 | 4123769 | *HEI10* | 1 | 571048 | *HEI10* | 2 | 6044013 |
| 38 | Wild type | 1 | 1535016 | Wild type | 2 | 2415976 | *HEI10* | 1 | 4768928 | *HEI10* | 2 | 5211484 |
| 39 | Wild type | 1 | 2344363 | Wild type | 2 | 1765212 | *HEI10* | 1 | 10798399 | *HEI10* | 2 | 1497196 |
| 40 | Wild type | 1 | 3348645 | Wild type | 2 | 835283 | *HEI10* | 1 | 4046307 | *HEI10* | 2 | 2467043 |
| 41 | Wild type | 1 | 1076845 | Wild type | 2 | 3793313 | *HEI10* | 1 | 913678 | *HEI10* | 2 | 791849 |
| 42 | Wild type | 1 | 2543997 | Wild type | 2 | 1927369 | *HEI10* | 1 | 400758 | *HEI10* | 2 | 823614 |
| 43 | Wild type | 1 | 2254491 | Wild type | 2 | 1885151 | *HEI10* | 1 | 757430 | *HEI10* | 2 | 1796518 |
| 44 | Wild type | 1 | 1771339 | Wild type | 2 | 925301 | *HEI10* | 1 | 499373 | *HEI10* | 2 | 2987639 |
| 45 | Wild type | 1 | 860721 | Wild type | 2 | 1668508 | *HEI10* | 1 | 250918 | *HEI10* | 2 | 1399199 |
| 46 | Wild type | 1 | 229789 | Wild type | 2 | 2469246 | *HEI10* | 1 | 1199496 | *HEI10* | 2 | 2464965 |
| 47 | Wild type | 1 | 409299 | Wild type | 2 | 3110838 | *HEI10* | 1 | 319406 | *HEI10* | 2 | 1030930 |
| 48 | Wild type | 1 | 832426 | Wild type | 2 | 2425398 | *HEI10* | 1 | 1368984 | *HEI10* | 2 | 2664424 |
| 49 | Wild type | 1 | 1136182 | Wild type | 2 | 1922734 | *HEI10* | 1 | 600264 | *HEI10* | 2 | 1879591 |
| 50 | Wild type | 1 | 1509573 | Wild type | 2 | 2231261 | *HEI10* | 1 | 816313 | *HEI10* | 2 | 4707151 |
| 51 | Wild type | 1 | 577604 | Wild type | 2 | 2719320 | *HEI10* | 1 | 1242446 | *HEI10* | 2 | 2663493 |
| 52 | Wild type | 1 | 1147036 | Wild type | 2 | 1396905 | *HEI10* | 1 | 276141 | *HEI10* | 2 | 1464787 |
| 53 | Wild type | 1 | 855187 | Wild type | 2 | 1972540 | *HEI10* | 1 | 330492 | *HEI10* | 2 | 2051929 |
| 54 | Wild type | 1 | 561583 | Wild type | 2 | 1410374 | *HEI10* | 1 | 774796 | *HEI10* | 2 | 927806 |
| 55 | Wild type | 1 | 1203861 | Wild type | 2 | 1875264 | *HEI10* | 1 | 345172 | *HEI10* | 2 | 3039472 |
| 56 | Wild type | 1 | 1396923 | Wild type | 2 | 3415582 | *HEI10* | 1 | 1098526 | *HEI10* | 2 | 2141659 |
| 57 | Wild type | 1 | 205757 | Wild type | 2 | 5017015 | *HEI10* | 1 | 2453963 | *HEI10* | 2 | 3219274 |
| 58 | Wild type | 1 | 878425 | Wild type | 2 | 4220023 | *HEI10* | 1 | 1861040 | *HEI10* | 2 | 314949 |
| 59 | Wild type | 1 | 931498 | Wild type | 2 | 3654711 | *HEI10* | 1 | 2430578 | *HEI10* | 2 | 4160326 |
| 60 | Wild type | 1 | 3663574 | Wild type | 2 | 2313520 | *HEI10* | 1 | 3757420 | *HEI10* | 2 | 3993142 |
| 61 | Wild type | 1 | 2660768 | Wild type | 2 | 838417 | *HEI10* | 1 | 1344445 | *HEI10* | 2 | 828404 |
| 62 | Wild type | 1 | 2778055 | Wild type | 2 | 1483558 | *HEI10* | 1 | 1033561 | *HEI10* | 2 | 1398489 |
| 63 | Wild type | 1 | 3213276 | Wild type | 2 | 2241034 | *HEI10* | 1 | 8915871 | *HEI10* | 2 | 5287125 |
| 64 | Wild type | 1 | 529374 | Wild type | 2 | 4497587 | *HEI10* | 1 | 3550999 | *HEI10* | 2 | 1607361 |
| 65 | Wild type | 1 | 2292404 | Wild type | 2 | 1010093 | *HEI10* | 1 | 6921406 | *HEI10* | 2 | 1925151 |
| 66 | Wild type | 1 | 1973449 | Wild type | 2 | 898358 | *HEI10* | 1 | 2657146 | *HEI10* | 2 | 1439824 |
| 67 | Wild type | 1 | 826179 | Wild type | 2 | 765932 | *HEI10* | 1 | 491514 | *HEI10* | 2 | 1634983 |
| 68 | Wild type | 1 | 272509 | Wild type | 2 | 939435 | *HEI10* | 1 | 1821854 | *HEI10* | 2 | 865127 |
| 69 | Wild type | 1 | 2725381 | Wild type | 2 | 522341 | *HEI10* | 1 | 483586 | *HEI10* | 2 | 448107 |
| 70 | Wild type | 1 | 1020272 | Wild type | 2 | 636015 | *HEI10* | 1 | 1786932 | *HEI10* | 2 | 258772 |
| 71 | Wild type | 1 | 1432345 | Wild type | 2 | 419297 | *HEI10* | 1 | 122223 | *HEI10* | 2 | 1243978 |
| 72 | Wild type | 1 | 1252875 | Wild type | 2 | 275599 | *HEI10* | 1 | 3918281 | *HEI10* | 2 | 1926608 |
| 73 | Wild type | 1 | 8484841 | Wild type | 2 | 3266314 | *HEI10* | 1 | 1008109 | *HEI10* | 2 | 2137760 |
| 74 | Wild type | 1 | 3497042 | Wild type | 2 | 2216097 | *HEI10* | 1 | 623704 | *HEI10* | 2 | 1251686 |
| 75 | Wild type | 1 | 2810673 | Wild type | 2 | 1777701 | *HEI10* | 1 | 154375 | *HEI10* | 2 | 2662955 |
| 76 | Wild type | 1 | 5346639 | Wild type | 2 | 2920566 | *HEI10* | 1 | 730922 | *HEI10* | 2 | 1756525 |
| 77 | Wild type | 1 | 3738088 | Wild type | 2 | 1556547 | *HEI10* | 1 | 301727 | *HEI10* | 2 | 1676884 |
| 78 | Wild type | 1 | 4653464 | Wild type | 2 | 972034 | *HEI10* | 1 | 503514 | *HEI10* | 2 | 2351081 |
| 79 | Wild type | 1 | 2548816 | Wild type | 2 | 1068091 | *HEI10* | 1 | 1373234 | *HEI10* | 2 | 1734020 |
| 80 | Wild type | 1 | 4776073 | Wild type | 2 | 947843 | *HEI10* | 1 | 814212 | *HEI10* | 2 | 2517737 |
| 81 | Wild type | 1 | 1398399 | Wild type | 2 | 1042485 | *HEI10* | 1 | 2486187 | *HEI10* | 2 | 2676382 |
| 82 | Wild type | 1 | 1760093 | Wild type | 2 | 822644 | *HEI10* | 1 | 2936941 | *HEI10* | 2 | 2892524 |
| 83 | Wild type | 1 | 1045145 | Wild type | 2 | 360373 | *HEI10* | 1 | 3602037 | *HEI10* | 2 | 1167799 |
| 84 | Wild type | 1 | 2336253 | Wild type | 2 | 124823 | *HEI10* | 1 | 1670490 | *HEI10* | 2 | 2201224 |
| 85 | Wild type | 1 | 2391168 | Wild type | 2 | 719932 | *HEI10* | 1 | 1085195 | *HEI10* | 2 | 1860829 |
| 86 | Wild type | 1 | 757475 | Wild type | 2 | 541072 | *HEI10* | 1 | 229882 | *HEI10* | 2 | 2595367 |
| 87 | Wild type | 1 | 1459104 | Wild type | 2 | 105446 | *HEI10* | 1 | 1623905 | *HEI10* | 2 | 628004 |
| 88 | Wild type | 1 | 621808 | Wild type | 2 | 1024191 | *HEI10* | 1 | 695053 | *HEI10* | 2 | 1943049 |
| 89 | Wild type | 1 | 2091978 | Wild type | 2 | 1571163 | *HEI10* | 1 | 937141 | *HEI10* | 2 | 1154409 |
| 90 | Wild type | 1 | 1990047 | Wild type | 2 | 1622474 | *HEI10* | 1 | 1591272 | *HEI10* | 2 | 732713 |
| 91 | Wild type | 1 | 1473152 | Wild type | 2 | 2734481 | *HEI10* | 1 | 691829 | *HEI10* | 2 | 1009296 |
| 92 | Wild type | 1 | 4531242 | Wild type | 2 | 1684078 | *HEI10* | 1 | 730349 | *HEI10* | 2 | 1324859 |
| 93 | Wild type | 1 | 2291280 | Wild type | 2 | 1032088 | *HEI10* | 1 | 513944 | *HEI10* | 2 | 1120444 |
| 94 | Wild type | 1 | 2140701 | Wild type | 2 | 377926 | *HEI10* | 1 | 676653 | *HEI10* | 2 | 724201 |
| 95 | Wild type | 1 | 1808273 | Wild type | 2 | 488302 | *HEI10* | 1 | 666868 | *HEI10* | 2 | 1108730 |
| 96 | Wild type | 1 | 3787948 | Wild type | 2 | 1260848 | *HEI10* | 1 | 420400 | *HEI10* | 2 | 3378153 |

**Supplemental Table S26. Arabidopsis centromeric, pericentromeric heterochromatin and euchromatic arm regions.** We define the centromeres genetically as contiguous regions flanking the TAIR10 centromeric assembly gaps that show an absence of crossovers in wild type (Copenhaver et al. 1999; Giraut et al. 2011; Salomé et al. 2012). We define the pericentromeric regions as regions flanking the centromeres with higher than chromosome average DNA methylation (Stroud et al. 2013; Yelina et al. 2015). The euchromatic arms constitute the remainder of the chromosomes, from the telomeres to the pericentromeres.

| Chr | North  Arm | North  Pericentromere | Centromere | South Pericentromere | South  Arm |
| --- | --- | --- | --- | --- | --- |
| 1 | 1 - 11,330,000 | 11,330,001 -  14,010,000 | 14,010,001-  15,980,000 | 15,980,001 -18,480,000 | 18,480,001 – 30,427,671 |
| 2 | 1 – 990,000 | 990,001 – 2,880,000 | 2,880,001 -  4,750,000 | 4,750,001 -  7,540,000 | 7,540,001 – 19,698,289 |
| 3 | 1 – 10,200,000 | 10,200,001 – 12,680,000 | 12,680,001 – 14,940,000 | 14,940,001 -  16,860,000 | 16,860,001 –  23,459,830 |
| 4 | 1 –  990,000 | 990,001 – 3,390,000 | 3,390,001 – 4,880,000 | 4,880,001 – 6,850,000 | 6,850,001 – 18,585,056 |
| 5 | 1 – 8,890,000 | 8,890,001 – 10,670,000 | 10,670,001 – 13,240,000 | 13,240,001 – 15,650,000 | 15,650,001 –  26,975,502 |

**Supplemental Table S27. Crossover numbers in chromosome arm, pericentromere or centromere regions identified in wild type or *HEI10^Col^* F_2_ mapping populations.** To test for significant differences in crossovers the counts within and outside a given region, e.g. data from chromosome arms from wild type and *HEI10^Col^* populations were used to construct 2×2 contingency tables and *X^2^* tests performed.

| Wild type | Chr1 | Chr2 | Chr3 | Chr4 | Chr5 | Total | *X^2^ P* |
| --- | --- | --- | --- | --- | --- | --- | --- |
| Arms | 231 | 142 | 178 | 154 | 226 | 931 |  |
| Pericentromeres | 84 | 68 | 47 | 44 | 56 | 299 |  |
| Centromeres | 0 | 0 | 0 | 0 | 0 | 0 |  |
| Total | 315 | 210 | 225 | 198 | 282 | 1,230 |  |
|  |  |  |  |  |  |  |  |
| *HEI10^Col^* | Chr1 | Chr2 | Chr3 | Chr4 | Chr5 | Total |  |
| Arms | 588 | 421 | 469 | 387 | 580 | 2,445 | 5.19×10^-9^ |
| Pericentromeres | 177 | 102 | 73 | 29 | 90 | 471 | 6.16×10^-10^ |
| Centromeres | 0 | 2 | 4 | 1 | 5 | 12 | 0.0533 |
| Total | 765 | 525 | 546 | 417 | 675 | 2,928 |  |

**Supplemental Table S28. Base pair overlaps between crossovers, gene and transposon annotations in wild type and *HEI10^Col^*.** The base pair coordinates that TIGER estimated crossovers to occur within were overlapped with TAIR10 representative genes or Arabidopsis transposon annotation. The same number and size of randomly chosen windows were analysed as a control (random). This was performed separately for chromosome arms, pericentromeres and centromeres (Supplemental Table S26).

| Wild type | Crossovers | | | | Random | | | |
| --- | --- | --- | --- | --- | --- | --- | --- | --- |
|  | Genes | TEs | Other | Total | Genes | TEs | Other | Total |
| Arms | 539,601 | 41,255 | 415,905 | 996,761 | 590,949 | 91,539 | 314,273 | 996,761 |
| Pericentromeres | 81,233 | 26,509 | 59,929 | 167,671 | 41,338 | 67,534 | 58,799 | 167,671 |
| Centromeres | 0 | 0 | 0 | 0 | 0 | 0 | 0 | 0 |
| Total | 620,834 | 67,764 | 475,834 | 1,164,432 | 632,287 | 159,073 | 373,072 | 1,164,432 |
| *HEI10^Col^* | Crossovers | | | | Random | | | |
|  | Genes | TEs | Other | Total | Genes | TEs | Other | Total |
| Arms | 1,452,368 | 116,913 | 1,091,884 | 2,661,165 | 1,528,057 | 235,498 | 897,610 | 2,661,165 |
| Pericentromeres | 126,713 | 46,468 | 105,040 | 278,221 | 779,65 | 128,910 | 71,346 | 278,221 |
| Centromeres | 766 | 1,401 | 1,017 | 3,184 | 1,817 | 1,200 | 167 | 3,184 |
| Total | 1,579,847 | 164,782 | 1,197,941 | 2,942,570 | 1,607,839 | 365,608 | 969,123 | 2,942,570 |

**Supplemental Table S29. Genotyping primers.**

| Primer | Sequence |
| --- | --- |
| *hei10-2* | 5'-AAGGAGTTCCCAGAGATGCTC-3' |
| *hei10-2* | 5'-GCCAGCAAGACAGAACAGTTC-3' |
| *msh4-1* | 5'-CGGCTTCACTGCATCTATCTC-3 |
| *msh4-1* | 5'-TGGAATGGATCAATGAGTTCC-3' |
| *msh5-1* | 5'­-AACCGATCGTCATTTGTTCTG-3' |
| *msh5-1* | 5'-CACTAAGGCCTGCTGAATTTG-3' |
| *ptd-1* | 5'-TCATGTCTTGCACCAACAGAG-3' |
| *ptd-1* | 5'-AAATCGACACCAAAATTTTGC-3' |
| *shoc1-1* | 5'-CTATTTGCAGAAGGATGACGG-3' |
| *shoc1-1* | 5'-CCTTTCCCTGAAATTCCTCAG-3' |
| *HEI10-XbaI* | 5'‑AATCTAGACTGGAATCAACAACGCAGTG‑3' |
| *HEI10-BamHI* | 5'‑TTGGATCCTAAGCCTTCAATGAACATCAC‑3' |
| *HEI10-qPCR1* | 5'-GGCATTGAAATCAGCGAGAG-3' |
| *HEI10-qPCR2* | 5'-ACCTGCGATTTTGACAGAGC-3' |
| *DMC1-qPCR1* | 5'-TGAAGAAACGAGCCAGATGC-3' |
| *DMC1-qPCR2* | 5'-GCGTTTATACCTTGTGCGATCA-3' |

**Supplemental Table S30. SSLP primers used for *rQTL* mapping.**

| Primer | Col product size (bp) | Ler product size (bp) | Chr | TAIR10  Coordinate | Nearest gene | Sequence |
| --- | --- | --- | --- | --- | --- | --- |
| 1-171-F | 149 | 135 | 1 | 171290 | At1g01460 | CTTAACTCTTTGGCCTTGGT |
| 1-171-R | 149 | 135 | 1 | 171290 | At1g01460 | CCGTTCGTATGGAATTTCTA |
| 1-3830-F | 130 | 98 | 1 | 3830600 | At1g11370 | CAACAATGGTGATATTTGTTTTGC |
| 1-3830-R | 130 | 98 | 1 | 3830600 | At1g11370 | TCAACAATGGGAAATGATCAAA |
| 1-7294-F | 199 | 162 | 1 | 7294957 | At1g20930 | TTCAAAACTGGAGCGTCGTC |
| 1-7294-R | 199 | 162 | 1 | 7294957 | At1g20930 | GGCCCATCTTGTGTGTTTTG |
| 1-10655-F | 230 | 167 | 1 | 10655852 | At1g30270 | TTGTGGTCCCTGGCTAATCA |
| 1-10655-R | 230 | 167 | 1 | 10655852 | At1g30270 | CAGTGACGAATTCCAAAACGA |
| 1-14122-F | 239 | 189 | 1 | 14122817 | At1g37100 | GCTAGCAGTCGAGTATTCTGTCGAG |
| 1-14122-R | 239 | 189 | 1 | 14122817 | At1g37100 | CGTGTCCCACCATCATCAC |
| 1-16908-F | 110 | 82 | 1 | 16907783 | At1g44770 | GCACAGAAAGACAAACCCAAAG |
| 1-16908-R | 110 | 82 | 1 | 16907783 | At1g44770 | CGACCAGCAAGGTTGTTCTTAG |
| 1-17135-F | 482 | 398 | 1 | 17135019 | At1g45211 | CCTATGTTTCGGCATTCAGG |
| 1-17135-R | 482 | 398 | 1 | 17135019 | At1g45211 | GCATCGTTAGACGGTTTGCT |
| 1-18570-F | 128 | 162 | 1 | 18569811 | At1g50140 | CGTACAGTGTTTCGTGTTCCA |
| 1-18570-R | 128 | 162 | 1 | 18569811 | At1g50140 | TCTCCTTTTGGCTTCTGATGA |
| 1-19077-F | 163 | 128 | 1 | 19076880 | At1g51450 | CCATCTTCTGTTTATTTGATTTCCA |
| 1-19077-R | 163 | 128 | 1 | 19076880 | At1g51450 | AAGATAAGCATGGCTACATTATCAGA |
| 1-19540-F | 221 | 286 | 1 | 19539729 | At1g52440 | GTTCCCCGATTCATGTGAGA |
| 1-19540-R | 221 | 286 | 1 | 19539729 | At1g52440 | CAAAAAGGGAAAAGCCCACT |
| 1-20154-F | 620 | 358 | 1 | 20154053 | At1g54000 | TCCCAACTGGTAATGATATTTATTTTC |
| 1-20154-R | 620 | 358 | 1 | 20154053 | At1g54000 | CCGAATCAAAATCGGAATCTT |
| 1-20606-F | 157 | 203 | 1 | 20606439 | At1g55240 | AATTGTTGCCTTGTTGGATGT |
| 1-20606-R | 157 | 203 | 1 | 20606439 | At1g55240 | ACGTTCAGTTCCCAATGAGC |
| 1-21236-F | 476 | 340 | 1 | 21236506 | At1g56650 | CAATGAGCCCTCTACGCTCT |
| 1-21236-R | 476 | 340 | 1 | 21236506 | At1g56650 | AAGCCCATCATATCCCAACA |
| 1-23477-F | 183 | 129 | 1 | 23477122 | At1g63295 | TGCTTTTCCTTTTTAATCTTTTTCTCA |
| 1-23477-R | 183 | 129 | 1 | 23477122 | At1g63295 | TGATGATTTGTTTTAATCCGCTCA |
| 1-26718-F | 179 | 104 | 1 | 26718054 | At1g70860 | TGTCTTGTTAAAAAGTAAAAACAC |
| 1-26718-R | 179 | 104 | 1 | 26718054 | At1g70860 | GTTTACTATCGTGAAAGTTGTTA |
| 1-30413-F | 135 | 104 | 1 | 30412519 | At1g80950 | CCAGCCACAGCTTCTTTCTGA |
| 1-30413-R | 135 | 104 | 1 | 30412519 | At1g80950 | TTGATTGAATAATGGTTCTTGTGATGA |
| 1-19621-F | 335 | 241 | 1 | 19620819 | At1g52690 | TTCGGGTGATTAGTACGGAAA |
| 1-19621-R | 335 | 241 | 1 | 19620819 | At1g52690 | TGAAAACCGTTACCCCCATA |
| 1-19799-F | 120 | 120 | 1 | 19798694 | At1g53130 | GCAAACTACAGTTCCTAGAATCGAA |
| 1-19799-R | 120 | 120 | 1 | 19798694 | At1g53130 | GGATCTTTCTTGATCTCCTTACCA |
| 1-19898-F | 128 | 128 | 1 | 19897734 | At1g53330 | CACAGTGTGCAAAGATCAAGG |
| 1-19898-R | 128 | 128 | 1 | 19897734 | At1g53330 | TTTGCCTGCAGATAATCCATC |
| 1-19909-F | 207 | 207 | 1 | 19909294 | At1g53360 | TTTATAAAAGTATGAGTCCGTATTTCACA |
| 1-19909-R | 207 | 207 | 1 | 19909294 | At1g53360 | AGCATGAAAACCCAACGGTA |
| 1-20003-F | 534 | 534 | 1 | 20003399 | At1g53600 | CCTGGGTCTAGCTGGATCAT |
| 1-20003-R | 534 | 534 | 1 | 20003399 | At1g53600 | AAGGCGATCAAGTGAGCTTC |
| 1-20072-F | 120 | 120 | 1 | 20072074 | At1g53770 | ATCTTCGTAATGAAATGAACTGAGC |
| 1-20072-R | 120 | 120 | 1 | 20072074 | At1g53770 | TATCACTTGTACGAAGAATATGGTGTG |
| 1-20154-F | 620 | 358 | 1 | 20154053 | At1g54000 | TCCCAACTGGTAATGATATTTATTTTC |
| 1-20154-R | 620 | 358 | 1 | 20154053 | At1g54000 | CCGAATCAAAATCGGAATCTT |
| 1-20606-F | 157 | 203 | 1 | 20606439 | At1g55240 | AATTGTTGCCTTGTTGGATGT |
| 1-20606-R | 157 | 203 | 1 | 20606439 | At1g55240 | ACGTTCAGTTCCCAATGAGC |
| 1-19540-F | 221 | 286 | 1 | 19539729 | At1g52440 | GTTCCCCGATTCATGTGAGA |
| 1-19540-R | 221 | 286 | 1 | 19539729 | At1g52440 | CAAAAAGGGAAAAGCCCACT |
| 2-132-F | 229 | 162 | 2 | 132648 | At2g01250 | TCCAATGGGCCACAAATTAAC |
| 2-132-R | 229 | 162 | 2 | 132648 | At2g01250 | TTTGTGCTTTGATTACTGCAAGTG |
| 2-2346-F | 347 | 261 | 2 | 2346993 | At2g06020 | GGCAAATTTGGTTGGCTCTC |
| 2-2346-R | 347 | 261 | 2 | 2346993 | At2g06020 | TGTTTTGTGCTATTTGTGTCAACC |
| 2-6789-F | 112 | 82 | 2 | 6789815 | At2g15560 | GCGTTTTGTATCATCAAAGGTTCC |
| 2-6789-R | 112 | 82 | 2 | 6789815 | At2g15560 | CGCAATTTCTCGAACTTCCTTT |
| 2-9391-F | 179 | 124 | 2 | 9391360 | At2g22100 | CGGTCACTGTGAGGTCATTG |
| 2-9391-R | 179 | 124 | 2 | 9391360 | At2g22100 | TTTTTGGTCATCGGTACTTGG |
| 2-11443-F | 200 | 141 | 2 | 11443153 | At2g26830 | GGTTCCGTCAACTTCGAAAA |
| 2-11443-R | 200 | 141 | 2 | 11443153 | At2g26830 | CAGTCATTAGAAATCGATCCCACA |
| 2-14714-F | 125 | 89 | 2 | 14714870 | At2g34880 | CAATTAAAGAGGTTTCAGTTTTCCAG |
| 2-14714-R | 125 | 89 | 2 | 14714870 | At2g34880 | CAGAGGGACTTGACGAAAGAG |
| 2-19311-F | 140 | 101 | 2 | 19311521 | At2g47000 | TTTCTGCCAATGATTTAAAGTAACG |
| 2-19311-R | 140 | 101 | 2 | 19311521 | At2g47000 | CAGCGCTGATGCAAAGGTAA |
| 4-230-F | 267 | 209 | 4 | 230388 | At4g00520 | GCGTTCACCTTTAGCATTCCA |
| 4-230-R | 267 | 209 | 4 | 230388 | At4g00520 | GCAGCTACACTCATGCCCTCT |
| 4-2450-F | 242 | 184 | 4 | 2450565 | At4g04840 | GCGATGATGTGCTTAGGTTGG |
| 4-2450-R | 242 | 184 | 4 | 2450565 | At4g04840 | GGATTCAATCACATTTCTTTTCAA |
| 4-4852-F | 146 | 108 | 4 | 4852373 | At4g08028 | TGGGCCAACGACTCTGTTTA |
| 4-4852-R | 146 | 108 | 4 | 4852373 | At4g08028 | TCGTTGTCGAACAACACACC |
| 4-9652-F | 234 | 172 | 4 | 9652287 | At4g17200 | GTTGCCCACTTGTGTGGTCT |
| 4-9652-R | 234 | 172 | 4 | 9652287 | At4g17200 | TCTTGTTTGGATGTGAAATTGGA |
| 4-10477-F | 186 | 140 | 4 | 10476582 | At4g19160 | TTGGCTGATCGACAAAGTGA |
| 4-10477-R | 186 | 140 | 4 | 10476582 | At4g19160 | GTAGTGCATGTTGCGTTTCG |
| 4-10847-F | 254 | 196 | 4 | 10846502 | At4g20030 | TTCCCTTCTTTTGTGGCTTC |
| 4-10847-R | 254 | 196 | 4 | 10846502 | At4g20030 | CCGTCACAATCCTGACTCAA |
| 4-11840-F | 147 | 110 | 4 | 11840149 | At4g22470 | ATTTACGGCGGTTCTTGATG |
| 4-11840-R | 147 | 110 | 4 | 11840149 | At4g22470 | TTTTTGGGTTCCAACAATGTAA |
| 4-12848-F | 138 | 100 | 4 | 12848948 | At4g24980 | CTCCAAGCTCCTTGTTTTGG |
| 4-12848-R | 138 | 100 | 4 | 12848948 | At4g24980 | AATCGTCCGGTCAATCTGAG |
| 4-12981-F | 180 | 128 | 4 | 12981959 | At4g25400 | GCTGAGGTACAATATCTCGAGCTTAC |
| 4-12981-R | 180 | 128 | 4 | 12981959 | At4g25400 | GACAAGATCGAAAACATTAACAAAGT |
| 4-14558-F | 168 | 136 | 4 | 14558575 | At4g29730 | AAATCAAAACCCCATGAAAGG |
| 4-14558-R | 168 | 136 | 4 | 14558575 | At4g29730 | TTGTGGGGTGAGGGAGTTAG |
| 4-18526-F | 478 | 319 | 4 | 18526361 | At4g39950 | GACGAACAAGGCAACCCATT |
| 4-18526-R | 478 | 319 | 4 | 18526361 | At4g39950 | CCGGTTTGTTCACCATCTCC |
| 5-332-F | 258 | 191 | 5 | 331691 | At5g01849 | CCACTTCACACATGGCTACTG |
| 5-332-R | 258 | 191 | 5 | 331691 | At5g01849 | CGGTTTAGGCAGGTGAGAGA |
| 5-3750-F | 137 | 97 | 5 | 3750331 | At5g11660 | ATGGTGGACCTGGGGGTAAC |
| 5-3750-R | 137 | 97 | 5 | 3750331 | At5g11660 | GCATGTAGGAAACACAAATCCTGA |
| 5-7064-F | 267 | 220 | 5 | 7064379 | At5g20840 | ACTGGCCTCGCCTTTCACTA |
| 5-7064-R | 267 | 220 | 5 | 7064379 | At5g20840 | AATCACAACTGTGCCCTCGTT |
| 5-10406-F | 350 | 272 | 5 | 10406321 | At5g28468 | TGTATAATTAGAGCCGTTCGTCGT |
| 5-10406-R | 350 | 272 | 5 | 10406321 | At5g28468 | TTTTGAAACTATCCAAATTACCCAAA |
| 5-16428-F | 229 | 183 | 5 | 16428466 | At5g41030 | TGTTGCCATGTTGATTTGATTG |
| 5-16428-R | 229 | 183 | 5 | 16428466 | At5g41030 | AGATTCAAGGTGGGGCGTGT |
| 5-19994-F | 169 | 109 | 5 | 19994907 | At5g49320 | TCTAAACCGAACTAAACCGTGAA |
| 5-19994-R | 169 | 109 | 5 | 19994907 | At5g49320 | CAAACCAAAACCTACTTTTTCCAA |
| 5-23287-F | 204 | 151 | 5 | 23287613 | At5g57500 | GAGATGTTGAGAAGCAGAGGAAA |
| 5-23287-R | 204 | 151 | 5 | 23287613 | At5g57500 | TGGCGTGAAATACTGAAGCAA |
| 5-26907-F | 270 | 200 | 5 | 26907352 | At5g67420 | TGTGGATCTTTATGACGTGTGC |
| 5-26907-R | 270 | 200 | 5 | 26907352 | At5g67420 | ACCATCTACTTCCATTCAAATAACG |

**Supplemental Table S31. dCAPS and CAPS primers used for *rQTL1* mapping.** dCAPS primers start with letter 'd', and CAPS primers starts with letter 'c'.

| Primer | Col product size (bp) | Ler product size (bp) | Chr | TAIR10 Coordinate | Nearest gene | Sequence | Enzyme |
| --- | --- | --- | --- | --- | --- | --- | --- |
| d1-19962-F | 135 | 101 | 1 | 19962254 | At1g53470 | ACTGTGTGGTATTGGAGGTCCATGAGAA | *Mbo*II |
| d1-19962-R | 135 | 101 | 1 | 19962254 | At1g53470 | GATTTCAGAGCACGTTTGTG |  |
| d1-19952-F | 131 | 97 | 1 | 19951991 | At1g53450 | AAACAGATGCAAATGTTGAAGGATGAGCTGTGAGT | *Apa*LI |
| d1-19952-R | 131 | 97 | 1 | 19951991 | At1g53450 | CAGTGTGTGCTCTACATTTGGTG |  |
| d1-19927-F | 130 | 98 | 1 | 19926881 | At1g53420 | GAGCTTGTACACATTATCGCAATTTGGATCATGAT | *Bcl*I |
| d1-19927-R | 130 | 98 | 1 | 19926881 | At1g53420 | TGTTTCCCTAGGTGGAGGTTT |  |
| d1-19977-F | 127 | 99 | 1 | 19976541 | At1g53520 | CCACTGAGGATTTCAATGTAGGAGAAACTCG | *Xho*I |
| d1-19977-R | 127 | 99 | 1 | 19976541 | At1g53520 | TCTGTTTCGTCGGGAGGAT |  |
| d1-19986-F | 130 | 96 | 1 | 19986236 | At1g53560 | CATTGTTCAATCTAATTTACACATTCTTCTAAAGAT | *Bgl*II |
| d1-19986-R | 130 | 96 | 1 | 19986236 | At1g53560 | GACACATTGATCTATCAAGCTCAT |  |
| c1-19940-F | 182 | 214 | 1 | 19939666 | At1g53430 | TGGTGAAGGAGGATTTGGAC | *Bcl*I |
| c1-19940-R | 182 | 214 | 1 | 19939666 | At1g53430 | GCAGCGCAGATATCATACCG |  |
| d1-19967-F | 131 | 104 | 1 | 19966592 | At1g53490 | ATCCGATCTTGGAAACAGAAGAGCTGCC | *Hpa*II |
| d1-19967-R | 131 | 104 | 1 | 19966592 | At1g53490 | GGGAACGAGAGAGCTGAGAA |  |
