# Supplementary figures and images for: Natural variation and dosage of the HEI10 meiotic E3 ligase control Arabidopsis crossover recombination
Source: Genes Dev. 2017 Feb 1;31(3):306–17. doi: 10.1101/gad.295501.116 (PMC5358726; doi:10.1101/gad.295501.116)

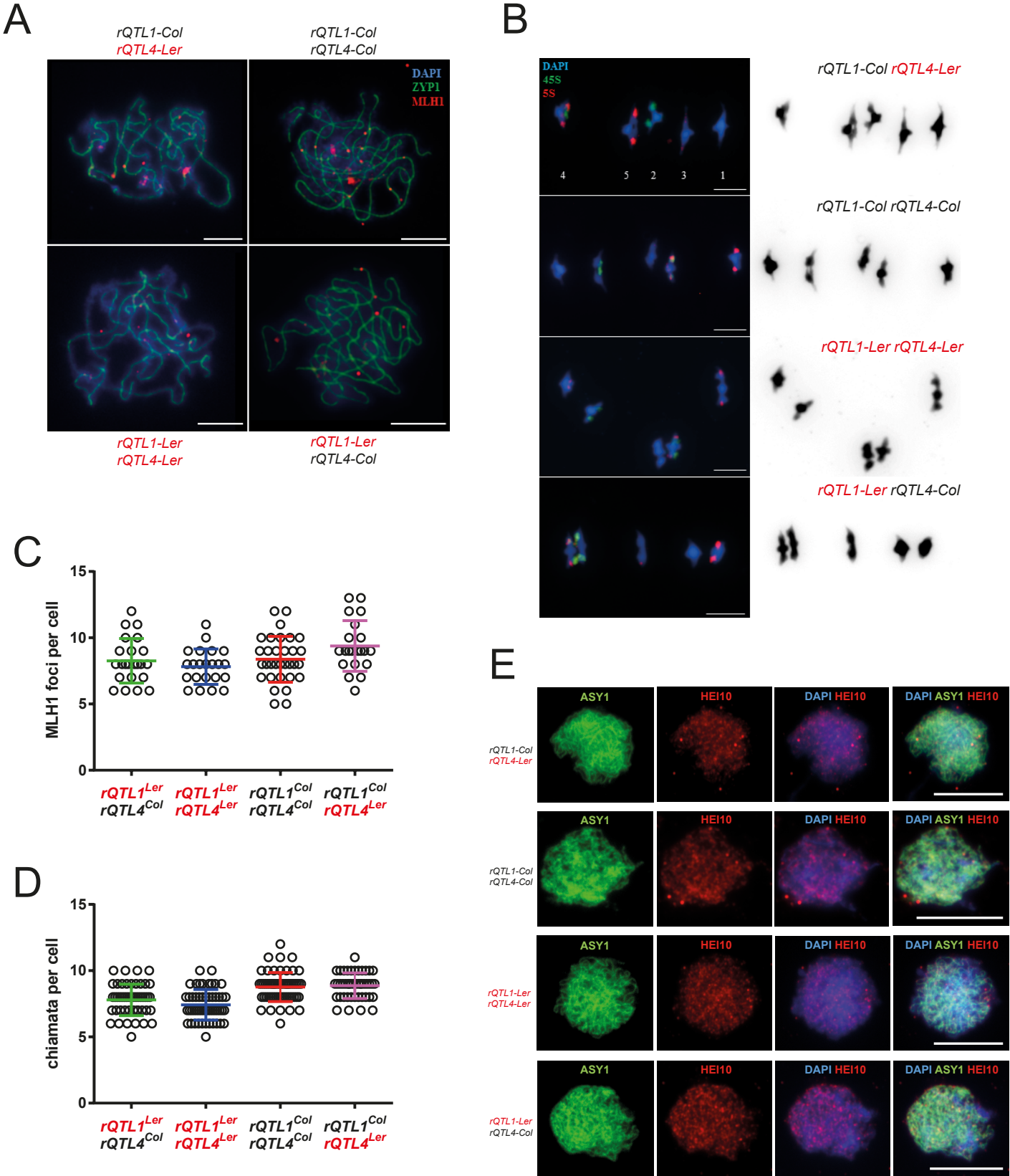

Supplement: Supplemental Material [file supp_gad.295501.116_Supplemental_FigS1.ps]

A

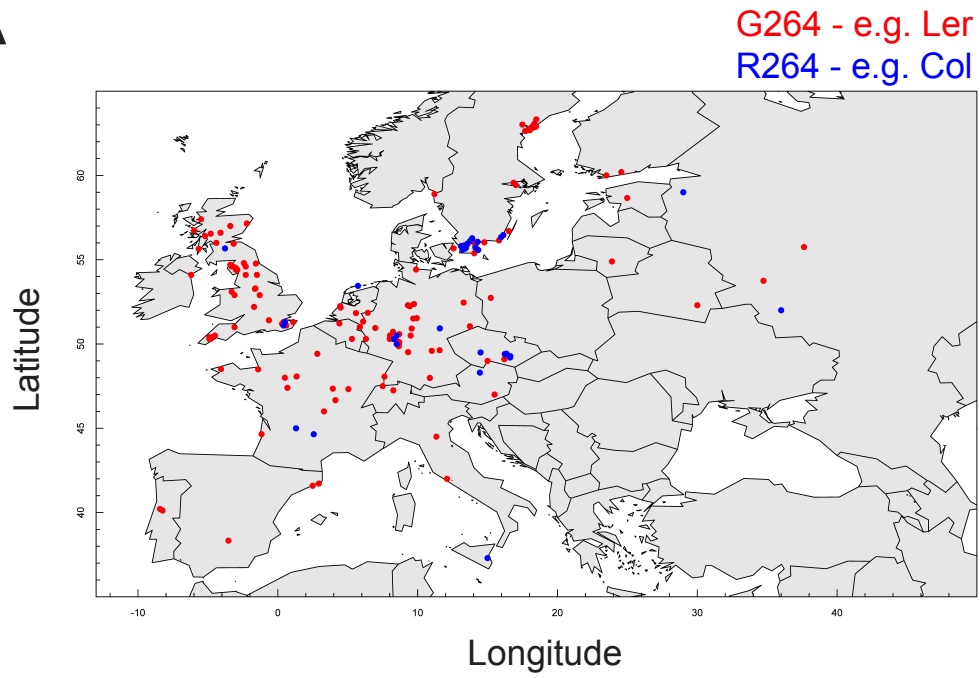

B

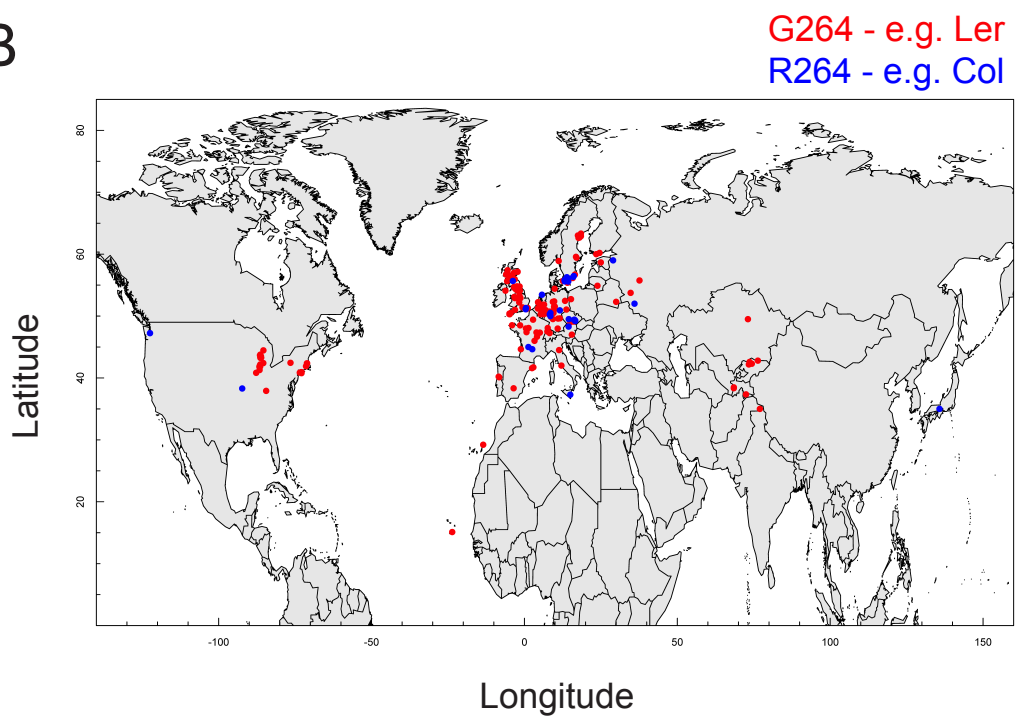

Supplement: Supplemental Material [file supp_gad.295501.116_Supplemental_FigS4.ps]

**A**

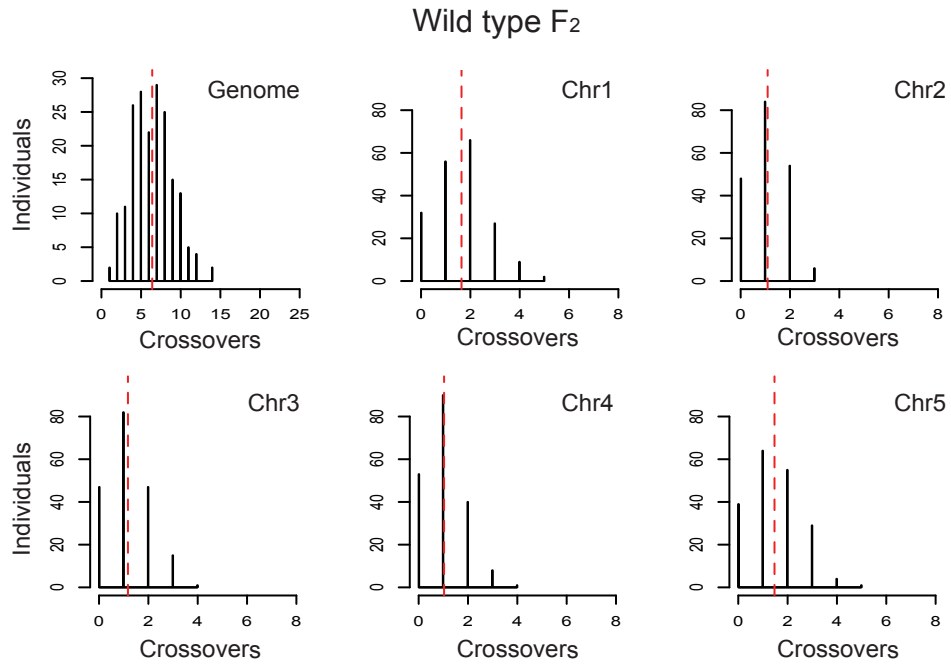

**B**

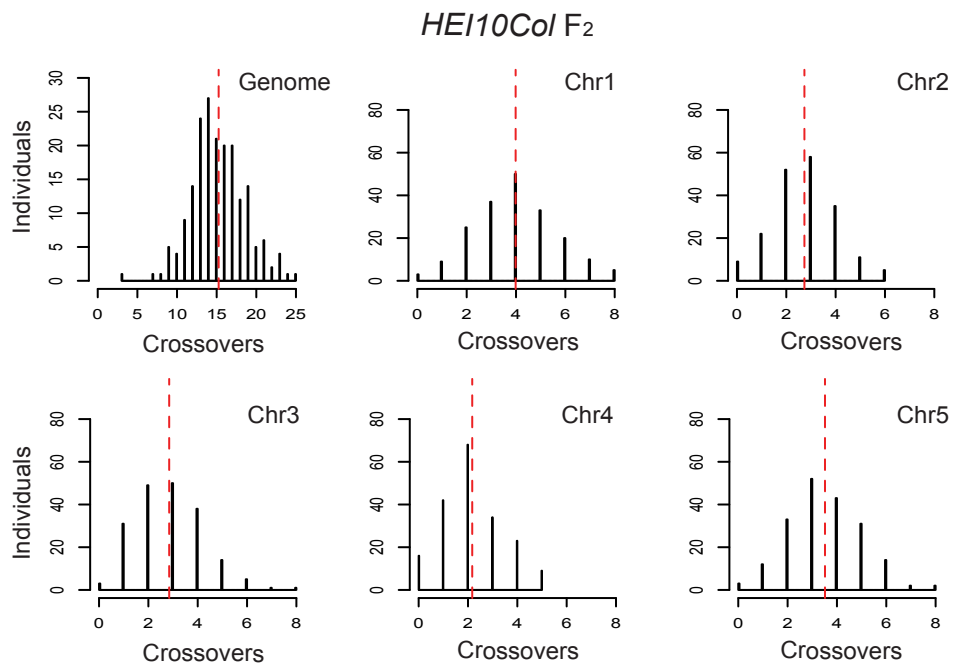

Supplement: Supplemental Material [file supp_gad.295501.116_Supplemental_FigS5.ps]

A

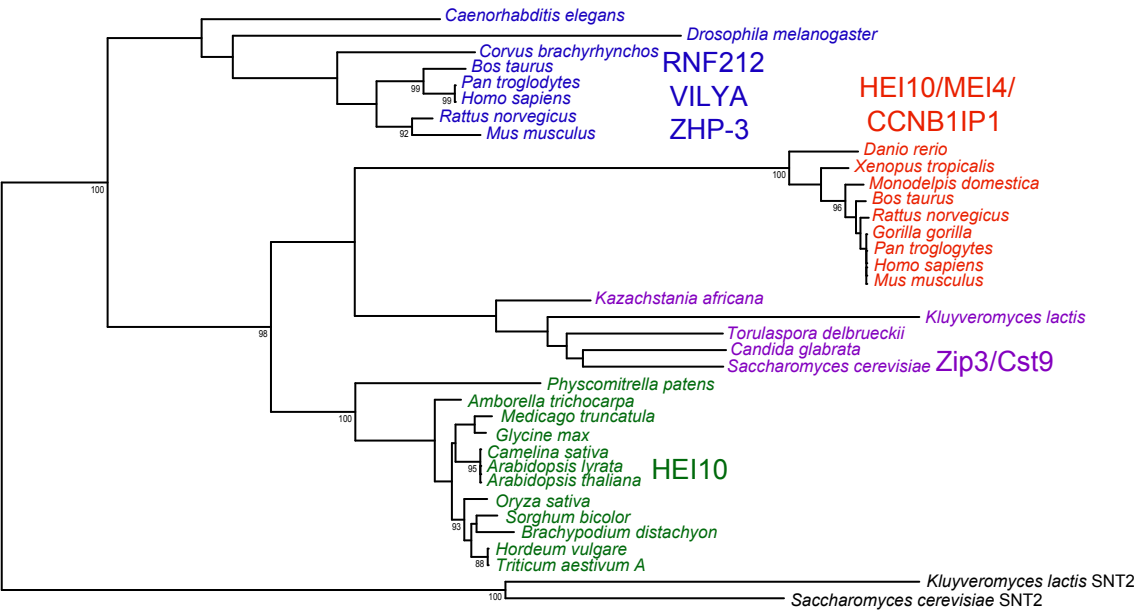

B

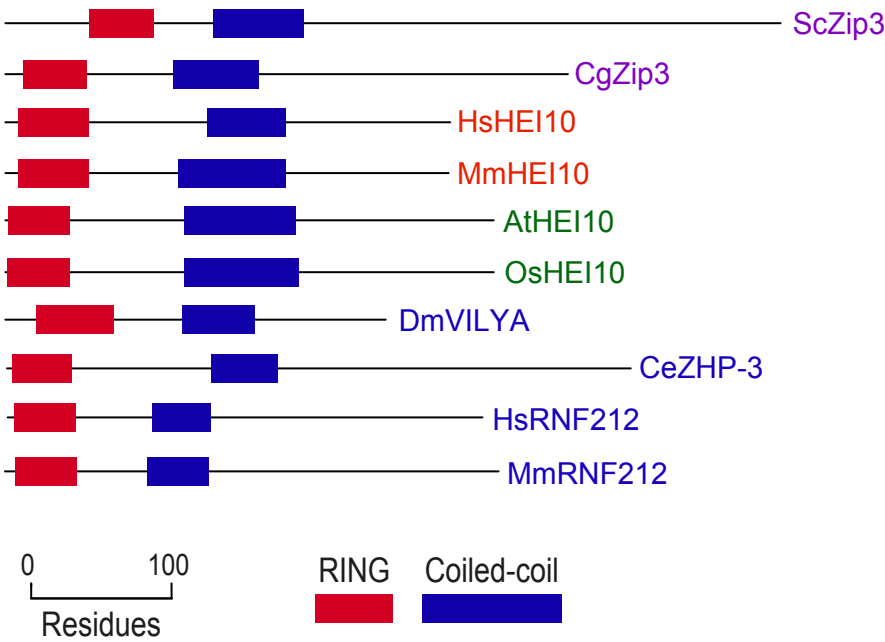

Supplement: Supplemental Material [file supp_gad.295501.116_Supplemental_FigS3.ps]

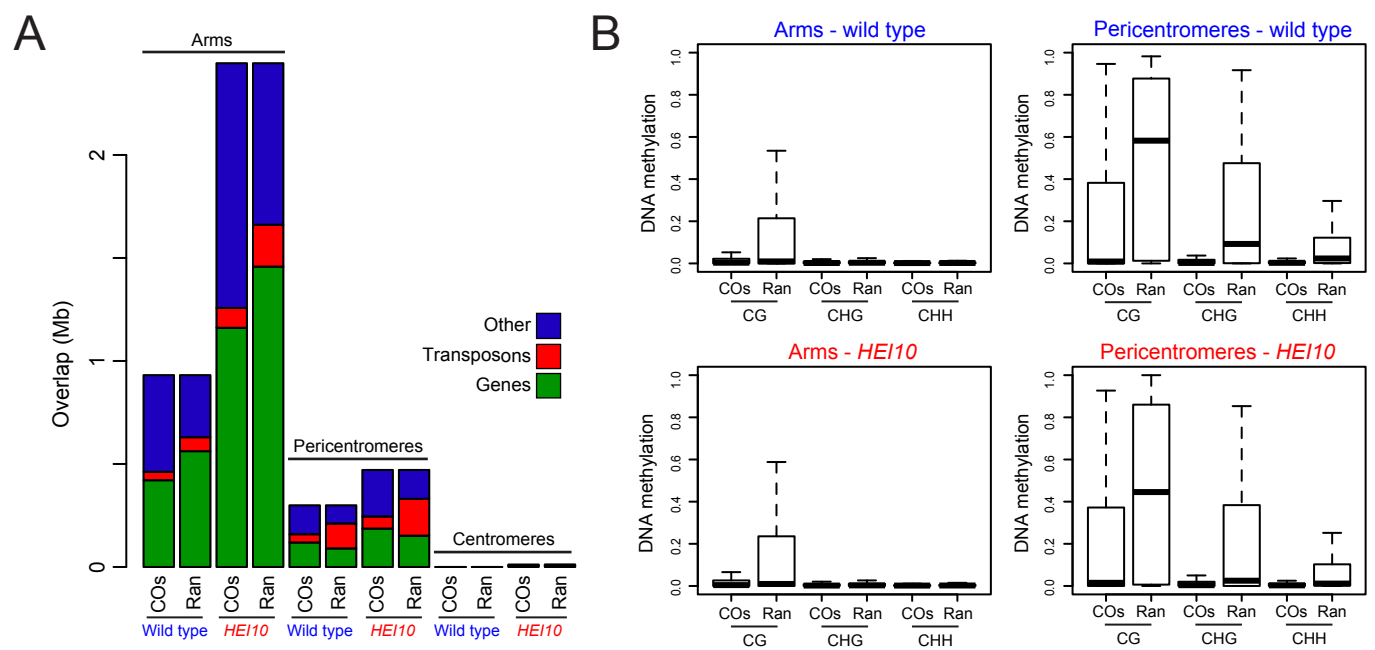

Supplement: Supplemental Material [file supp_gad.295501.116_Supplemental_FigS6.ps]
