# Supplementary material for: Natural variation and dosage of the HEI10 meiotic E3 ligase control Arabidopsis crossover recombination
Source: Genes Dev. 2017 Feb 1;31(3):306–17. doi: 10.1101/gad.295501.116 (PMC5358726; doi:10.1101/gad.295501.116)
Supplement: Supplemental Material [file supp_gad.295501.116_Supplemental_FigS2.docx]

Ziolkowski GENESDEV/2016/295501 Figure S2

CLUSTAL O(1.2.2) multiple sequence alignment

Panicum halli MKCNACWREVEGQAITTTCGHLLCTEDAKKILSNDGACPICDQVLSKSHMKPVDVDPNDD

Setaria italica MKCNACWRELEGQAITTTCGHLLCTEDAKKILSNDGACPICDQVLSKSHMKPMDVDPSDD

Sorghum bicolor MKCNACWRELEGQAISTTCGHLLCADDAKKILSNDGACPICDQVLSKSHMKPMDINPSDD

Zea mays MKCNACWRDLEGQAVSTTCGHLLCADDARKILNNDGTCPICDQVLSKSHMKPMDINPGDD

Oryza sativa MKCNACWRELEGQAVSTTCGHLLCTEDAKKILSNDAACPICDQVLSKSHMRPVDTNPNDD

Leersia perrieri MKCNACWRDLEGQAVSTTCGHLLCTEDAKKILSNDAACPVCDQVLSKSHMRPIDTNPSDD

Brachypodium distachyon MKCNACWREMEGQAISTTCGHLLCPEDAKKILSADGACPICDQVLSKSHMKPIDINPSDE

Triticum aestvium 6AL MKCNACWRELEGQAITTTCGHLLCTEDAKKILSNDGACPICDQVLSKSHMKPTDINPSDE

Triticum aestvium 6DS MKCNACWRELEGQAITTTCGHVLCTEDAKKILSNDGACPICDQVLSKSHMKPTDINPSDE

Triticum aestvium 6BS MKCNACWRELEGQAITTTCGHLLCTEDAKKILSNDGACPICDQVLSKSHMKPTDINPSDE

Linum usitatissimum MRCNACWRELDGRAVATTCGHILCTEDASKVLSNDAACPVCEQVLSKSHMKPVDVNPNDE

Beta vulgaris MRCNACWRELEGQAVSTSCGHLLCTGDAGKILNSDGACPICDQVLSKSLMRPVDINPNDE

Cucumis sativa MKCNACWRELEGRAVTTTCGHLLCTEDASKILSNDGACPICDQVLSKSLMKPVDINPNDE

Medicago truncatula MRCNACWREVEGRAISTTCGHLLCTEDANKILSNDGACPVCDQVLSKSLMKPVDVNPNDE

Glycine max MRCNACWREVEGRAISTTCGHLLCTDDANKILSNDGACPICDQVLSKSLMKPVDVNPNDE

Phaseolus vulgaris MRCNACWRELEGRAISTTCGHLLCTDDANKILSNDGACPICDQVLSKSLMKPVDVNPNDE

Malus domestica MRCNACWRELEGRAVSTTCGHLLCTEDAGKILGSDGACPVCDQVLSKSHMKAFDINPNDE

Prunus persica MRCNACWREVEGRAVSTTCGHLLCTEDASKILGNDGACPICDQVLSKSLMKPVDINPNDE

Vitis vinifera MRCNACWRELEGRAISTTCGHLLCSEDASKILSSDAACPICDQVLSKSLMKPVDVNPNDE

Eutrema salsugineum MRCNACWRELEGRAIATTCGHLLCTEDATKILSNDGACPICDQVLSKSLMKPVDTNPNEE

Boechera stricta MRCNACWRDLEGRAISTTCGHLLCTEDASKILSNDGACPICDQVLSKSLMKSVDINPNEE

Camelina sativa MRCNACWRDLEGRAISTTCGHLLCTEDASKILSNDGACPICDQVLSKSLMKPVDINPNEE

Capsella grandiflora MRCNACWRDLEGRAISTTCGHLLCTEDASKILSNDGACPICDQVLSKSLMKPVDINPNEE

Capsella rubella MRCNACWRDLEGRAISTTCGHLLCTEDASKILSNDGACPICDQVLSKSLMKPVDINPNEE

Arabidopsis lyrata MRCNACWRDLEGRAISTTCGHLLCTEDASKILSNDGACPICDQVLSKSLMKPVDINPNEE

Arabidopsis thaliana Col MRCNACWRDLEGRAISTTCGHLLCTEDASKILSNDGACPICDQVLSKSLMKPVDINPNEE

Arabidopsis thaliana Ler MRCNACWRDLEGRAISTTCGHLLCTEDASKILSNDGACPICDQVLSKSLMKPVDINPNEE

Eucalyptus grandis MRCNACWRELEGRAVSTTCGHLLCNEDAAKILNSDAACPICDQVLSKSHMKPVDINPNDE

Salix purpurea MRCNACWRELEERAVSTTCGHLLCTEDAGKILNSDAPCPICDQVLSESLMKPVEINPNDE

Populus trichocarpa MRCNSCWRELEGRAVSTTCGHLLCTEDANKILNNDAACPICDQVLSKSLMKPVEINPNDE

Manihot esculenta MRCNACWRELEGRAITTTCGHLLCTEDASKILSNDAACPICDQVLSKSLMKPVDINPNDE

Citrus sinensis MRCNACWRELEGRAISTTCGHLLCTEDANKILSNDAACPICDQVLSKSLMKPVDINPNDE

Theobroma cacao MRCNACWRELEGRAVSTTCGHLLCTEDASKILSNDAACPICDQVLSKSLMKPVDINPNDE

*:**:***::: :*::*:***:** ** *:* *. **:*:****:* *: : :* ::

_______________________________________

**RING domain**

Panicum halli WTNMAMAGISPQILMKSAYRSVMFYIGQKELEMQYKMNHVVGQCRQKIELMQGKFTEKLE

Setaria italica WTNMAMAGISPQILMKSAYRSVMFYIGQKELEMQYKMNHIVGQCRQKIELMQGKFTEKLE

Sorghum bicolor WTNMAMVGISPQILMKSAYRSVMFYIGQKELEMQYKMNRIVGQCRQKCELMQAKFTEKLE

Zea mays WTNMAMVGISPQILMKSAYRSVMFYIGQKELEMQYKMNRIVGQCRQKCELMQAKFTEKLE

Oryza sativa WTNMSMAGVSPQILMKSAYRSVMFYIGQKELEMQYKMNRIVGQCRQKCELMQAKFTEKLE

Leersia perrieri WTNMAMAGVSPQILMKSAYRSVMFYIGQKELEMNCKLNRILGQCRQKCELMQAKFTEKLE

Brachypodium distachyon WTNMSMVGISPQILMKSAYRSVMFYIGQKELEMQYKMNRVLGQCRQKCEHIQAKFTEKLE

Triticum aestvium 6AL WTDMSMTGVSPQILMKSAYRSVMFYIGQKDLEMQYKMNRIVGQCRQKCEVMQAKFTEKLE

Triticum aestvium 6DS WTNMSMTGVSPQILMKSAYRSVMFYIGQKDLEMQYKMNRIVGQCRQKCEVMQAKFTEKLE

Triticum aestvium 6BS WTNMSMTGVSPHILMKSAYRSVMFYIGQKDLEMQYKMNRIVGQCRQKCEVMQAKFTEKLE

Linum usitatissimum WTNMAMAGISPQTLMKTTYKGFMFYIGQKELEMQFKMNRIVAQCRQKCEALQEKCTEKLE

Beta vulgaris WINTVMAGVSPQILMKSAYRSVMFYIGQKELEMQCKMNRLVAQCRQKCETLQEKFSEKLE

Cucumis sativa WANMAMAGISPQILMKSAYRSVMFYNGQKELEMQYKMNRLMAQCRQKCEVMQEKFTEKLE

Medicago truncatula WVNMAMVGVSPQILMKSAYKSVMFYIGQKELEMQCKMNKIVGQCRQKCEMMQEKFTEKLE

Glycine max WVNMAMAGVSPQILMKSAYRSVMFYLGQKELEMQFKMNKIVAQCRQKCEMMQEKFTEKIE

Phaseolus vulgaris WVNMAMAGVSPQILMKSAYRSVMFYIGQKELEMQFKMNKIVAQCRQKCEMMQEKFTEKLE

Malus domestica WISMAMIGVSPQILMKSAHKSVMFYIGQRELEMQFKMNRIQAQYRQKFEAMQEKFGEKME

Prunus persica WVNMAMAGVSPQILMKSAHRSIMFYIGQKELEMQFKMNRIVAQFRQKCEAMQEKFTEKLE

Vitis vinifera WINMAMAGVSPQILMKSAYRSVMFYIGQKELEMQYKMNRIVAQCRQKCEMMQEKFTEKLE

Eutrema salsugineum WINMAMAGISPQILMKSAYRSVMFYISQRDLEMQYKMNRVVAQCRQKCEGMQAKFSEKME

Boechera stricta WINMVMAGISPQILMKSAYRSVMFYIAQRDLEMQYKMNRVGAQYRQKCEGMQAKFNEKME

Camelina sativa WINMAMAGISPQILMKSAYRSVMFYIVQRDLEMQYKMNRVVAQCRQKCEGMQAKFSEKME

Capsella grandiflora WINMAMAGISPQILMKSAYRSVMFYIAQRDLEMQYKMNRVVAQCRQKCEGMQAKFSEKME

Capsella rubella WINMAMAGISPQILMKSAYRSVMFYIAQRDLEMQYKMNRVVAQCRQKCEGMQAKFSEKME

Arabidopsis lyrata WINMAMAGISPQILMKSAYRSVMFYIAQRDLEMQYKMNRVVAQCRQKCEGMQAKFSEKME

Arabidopsis thaliana Col WINMAMAGISPQILMKSAYRSVMFYIAQRDLEMQYKMNRVVAQCRQKCEGMQAKFSEKME

Arabidopsis thaliana Ler WINMAMAGISPQILMKSAYRSVMFYIAQRDLEMQYKMNRVVAQCRQKCEGMQAKFSEKME

Eucalyptus grandis WINMAMAGISPQILMKSAYRSVMFFVGQKELEMQFKMNRIVAQCRQKCEVMQEKFTEKLE

Salix purpurea WINMAMAGISPEILMKSAYRSVMFYIGQRELEMQYKMNRVVAQCRQKCESMHEKFAEKLE

Populus trichocarpa WINMAMAGISPQILMKSAYRSVMFFTGQRELEMQYKMNRIVAQCRQKCESMQEKFTEKLE

Manihot esculenta WINTAMAGVSPQILMKSAYRSVMFYIGQKELEMQYKMNRIVAQCRQKCEVMQEKFTEKIE

Citrus sinensis WVNMAMAGVSPQILMKSAYRSVMFYLGQKELEMQYKMNRIVAQCRQKCEAMQEKFTEKLE

Theobroma cacao WINMAMAGVSPQILMKSSYRSVMFYIGQKELEMQYKMNRIVAQCRQKCEAMQEKFSEKLE

* . * *:**. ***::::..**: *::***: *:*:: .* *** * :: * **:*

_

Ziolkowski GENESDEV/2016/295501 Figure S2

Panicum halli ELHTAYQKMGKRCQLMEQEIENLTKDKQELQEKFAEKSRQKRKLDEMYDKLRTEYDSVKR

Setaria italica ELHAAYQKMGKKCQLMEQEIESLTKDKQELQEKFAEKSRQKRKLDEMYDKLRTEYDSLKR

Sorghum bicolor EVHTAYQKMAKRCQLMEQENENLNRDKQELQEKFAEKSRQKRKLDEMYDQLRNEYESLKR

Zea mays EVHTAYQKMAKRCQLLEQENENLNRDKHELQEKFAEKSRQKRKLDEMYDQLRNEYESVKR

Oryza sativa EVHTAYQKMAKKCQLMEQEVENLSRDKQELQEKFAEKSRQKRKLDEMYDQLRSEYESAKR

Leersia perrieri EVHTAYQKMAKKCQLMEQEIENLTRDKQELQEKFAEKSRQKRKLDEMYDQLRSEYESAKR

Brachypodium distachyon EVHTAYQKMGKRCQLMEQEIENLTRDKQELQEKFAEKSRQKRKLDEMYDQLRNEYESVKR

Triticum aestvium 6AL EVHAAYQKMAKRCQLMEQEIENLTRDKQELQEKFAEKSRQKRKLDEMYDQLRNEYESVKR

Triticum aestvium 6DS EVHAAYQKMAKRCQLMEQEIENLTRDKQELQEKFAEKSRQKRKLDEMYDQLRNEYESVKR

Triticum aestvium 6BS EVHAAYQKMAKRCQLMEQEIENLTRDKQELQEKFAEKSRQKRKLDEMYNRLRNEYESVKR

Linum usitatissimum QLHNAYQKMGKRCQMMEQEIESLRKDKQELQEKYSEQSRQKRNLDEMYDQLRSELESMKR

Beta vulgaris QMHAAYQKMGKRCQMMEQEIENLSKDKQELQEKFAEKSRQKRKLDEMYDQLRSEFDSVKR

Cucumis sativa QVHSAYQKMAKRCQMMEREMENLSKDKQELQEKFAEKSRQKRKLDEMYDQLRNEHESLKR

Medicago truncatula QLHTAYQKMAKRCQMMQQEIESLTKDNQELQEKFAEKSRQKRKLDEMYDQLRNEFDSVKR

Glycine max QVHTAYQKMAKKCQMMQQEIESLTKDNQELQEKFAEKSRQKRKLDEMYDQLRNEYDSVKR

Phaseolus vulgaris QVHTAYQKMAKKCQIMQQEIESLTKDNQELQEKFAEKSRQKRKLDEMYDQLRNEFDSVKR

Malus domestica QLHATYQKMAKRCQIMEQEIESLSKDKQELQEKFSEKSRQKRKLDEMYDQLRSEYESVKR

Prunus persica QVHTAYQKMAKRCQMMEQEIESLSKDKQELQEKFAEKSRQKRKLDEMYDQLRSEYESVKR

Vitis vinifera QVHTAYQKMAKRCQMMEQEIETMSKDKQELQEKFSEKSRQKRKLDEMYDQLRSEYESMKR

Eutrema salsugineum QVHTAYQKMGKRCQMMEQEVENLTKDKQELQEKFSEKSRQKRKLDEMYDQLRSEYESVKR

Boechera stricta QVHTAYQKMGKRCQMMEQEVESLTKDKQELQEKFSEKSRQKRKLDEMYDQLRSEYESVKR

Camelina sativa QVHTAYQKMGKRCQMMEQEVENLTKDKQELQEKFSEKSRQKRKLDEMYDQLRSEYESVKR

Capsella grandiflora QVHTAYQKMGKRCQMMEQEVENLTKDKQELQEKFSEKSRQKRKLDEMYDQLRSEYESVKR

Capsella rubella QVHTAYQKMGKRCQMMEQEVENLTKDKQELQEKFSEKSRQKRKLDEMYDQLRSEYESVKR

Arabidopsis lyrata QVHTAYQKMGKRCQMMEQEVENLTKDKQELQEKFSEKSRQKRKLDEMYDQLRSEYESVKR

Arabidopsis thaliana Col QVHTAYQKMGKRCQMMEQEVENLTKDKQELQEKFSEKSRQKRKLDEMYDQLRSEYESVKR

Arabidopsis thaliana Ler QVHTAYQKMGKRCQMMEQEVENLTKDKQELQEKFSEKSRQKRKLDEMYDQLRSEYESVKR

Eucalyptus grandis QVHAAYQKMAKRCQMMEQEIESLSKDKQELQEKFSEKSRQKRKLDEMYDQLRSDYESMKR

Salix purpurea QLHTAYQKITERCQMMEQEIDSLSKDKQELQEKFSEKSRQKRKLDEMYDQLRNEYDSMKR

Populus trichocarpa QLHAAYQKMAKRCQMMEQEIESLSKDKQELQEKFSEKARQKRKLDEMYDQLRSEYESNKR

Manihot esculenta QVHTAYQKVAKRCQMMEHEIESLSKDKQELQEKFSEKSRQKRKLDEMYDQLRSDYESMKR

Citrus sinensis QVHTAYQKMAKRCQMMEQEIESLSKDKQELQEKFSEKSRQKRKLDEMYDQLRSEYESMKR

Theobroma cacao QVHTAYQKMAKRCQMMEQEIESLSKDKQELQEKFSEKSRQKRKLDEMYDQLRSDYESMKR

::* :***: ::**::::* :.: :*::*****::*::****:*****::**.: :* **

_______________________________________________________

**Coiled coil domain**

Panicum halli SAIQPANNLFPRA--QQDLFAGMPNM-MDNSNPLRQ-G---------RREEMWAPAPRQR

Setaria italica SAIQPANNLFPRA--QQDLFSGMPNM-MDNSNPLRQ-G---------QRKEMWAPAPRQR

Sorghum bicolor SALQPANNYLARP--QSDPFAGMPNM-MDGGNHLRQ-GSVDLPKTPGQRDEGWGPPPRQR

Zea mays SALQPANNYLARP--QPDLFAGMPNV-MDGGDHLRQ-GSVDLPKTPGQRDEGWGPPPRQR

Oryza sativa SAIQPANNYFPRA--QPDLFSGVPNI-MDSSDPLRQ-GLAGLPETPGRRDEGWAPPPRQR

Leersia perrieri SAIQPANNYFPRA--RPDLFSGMPNI-MDSGDPLRQ-GSVDPPETPGRRDEGWAPPPRQH

Brachypodium distachyon SAIQPANNYFSRA--QPDLFSGMPNI-LDSGNPLRQ----------GRREEGWAPQPRQR

Triticum aestvium 6AL SAIQPANNYFPRA--QPDLFSGMPNI-LDSGDPLRQ-GSIDPPETPGRRDEGWAPQPRQR

Triticum aestvium 6DS SAIQPANNYFPRA--QPDLFSGMPNI-LDSGDPLRQ-GSIDPPETPGRRDEGWAPQPRQR

Triticum aestvium 6BS SAIQPANNYFPRA--QPDLLSGMPNI-LDSGDPLRQ-GSIDPPETPGRRDEGWAPQPRQR

Linum usitatissimum SAIQPASNFYGRRNNDVDLFADHGATMMDTREPNRNDWSAVTPTTPGLKEDIWT--TRQN

Beta vulgaris SAIQPAKNFYSR-P-EPDLFSHPTHM-MDNQDTLRKDRLVFTPETPGPREDIWP--ARQN

Cucumis sativa SAIQPVTNFYTR-N-ESDLFSNPVNL-MDNREATRKDWMVSAPETPGPREEIWP--TRQN

Medicago truncatula SAIQPASNFYSR-N-ERDLFSNPPNI-LDERETGRKGPPVFTPTTPGPREDVWP--ARQN

Glycine max SAIQPADNFYSR-N-EHDLFSNPPNI-MDGREMGRKGGLVFTPATPGPREDVWP--ARQN

Phaseolus vulgaris SAIQPANNFYSR-N-DHDLFSNPPNI-IDDRDIGRKGGPVFTPATPGPRDDVWP--ARQN

Malus domestica SAIQPSTNFFSR-N-EPDIFSNQANIMMDNREAVRKDWPVLTPETPGPKEDIWP--ARQN

Prunus persica SALQPATNFYSR-N-EPDLFSNQANI-LDNREAVRKDWPVLTPETPGPKEDIWP--ARQN

Vitis vinifera SAIQPTTNFYSR-P-EPDLFSNQPNI-MDNRDTIRKDWSVFTPETPGPKEDIWT--ARQN

Eutrema salsugineum TAIQPANNFYPR-HHEPDFFSNPPVNMMENREPTRKDRSFYSPATPGPKDEIWP--ARQN

Boechera stricta TAIQPANNFYPR-HQEPDFFSNPAVNMMENREPIRKDRSFFSPATPGPKDEIWP--ARQN

Camelina sativa TAIQPANNFYPR-HQEPDFFSNPAVNMMENREPIRKDRSFFSPATPGPKDEIWP--ARQN

Capsella grandiflora TAIQPANNFYPR-HQEADFFSNPAVNMMENREPIRKDRSFYSPATPGPKDEIWP--ARQN

Capsella rubella TAIQPANNFYPR-HQEADFFSNPAVNMMENREPIRKDRSFYSPATPGPKDEIWP--ARQN

Arabidopsis lyrata TAIQPANNFYPR-HQEPDFFSNPAVNMMENREPIRKDRSFFSPATPGPKDEIWP--ARQN

Arabidopsis thaliana Col TAIQPANNFYPR-HQEPDFFSNPAVNMMENRETIRKDRSFFSPATPGPKDEIWP--ARQN

Arabidopsis thaliana Ler TAIQPANNFYPR-HQEPDFFSNPAVNMMENRETIRKDRSFFSPATPGPKDEIWP--ARQN

Eucalyptus grandis SAIQPASNFYPR-S-EHDLFSAPV-NMMNNPDNMHKDWSIFPPPTPGQREDVWP--ARQN

Salix purpurea SAIQPANNLFSR-N-EPDLFANHAATMLDSRNPIW----------KGPREVSWP--ARQN

Populus trichocarpa SAIQPANNFFSR-N-EPDLFSNPAATMMDNRDPIRKDWTVFTPSTPGPREDIWP--ARQN

Manihot esculenta SAIQPTGNFYSR-N-EPDFFPNPATTMMDNREPMRKDWSVFNPPTPGPREDVWP--ARQN

Citrus sinensis SAIQPSNSFYPR-N-DPDLFS---ANMMDNRDPIRKDWSVYSPGTPGPREDIWP--ARQN

Theobroma cacao SAIQPSNNFYVR-N-EADLFSNPATNMVDGRDPIRRDWSIFSPKTPGPREDVWP--ARQN

:*:** . * * : :: : :. * **.

____________________________________________________________

**C-terminal domain**

Ziolkowski GENESDEV/2016/295501 Figure S2

Panicum halli HSNP-DTFEVSGGS-AHMGA-PPVDARPRRP--AGPVFGAG--TNNPSAALRNMLVSPVK

Setaria italica LSNP-DTFEISGGS-AHLGA-PPVDARPRRP--AGPVFGAG--TNNPSAALRNMLISPVK

Sorghum bicolor CSTS-GPFELSTGSPGHAMA-PPADIRPRQQ--TRPVFGAT--MNNPSSALRNFIISPVK

Zea mays HCTS-GPFDLSTGSPAHAVA-PPADIRPRQQ--TRSVFGAT--LNNPSSTLRNLIISPVK

Oryza sativa RSTS-GPFELSAGSPAHNAA-PPVDIRPRQP--ARPVFGTA--MNNTSAALRNMIISPVK

Leersia perrieri RSTS-GPFELSAGSPAHHAA-PQVDIRPRQP--ARPVFGTT--MNNTSAALRNMIISPVK

Brachypodium distachyon RENS-GPFELSAGSPGHTAA-PPMDMRPRQP--ARSTFGAN--MNNSSAALRNMIISPMK

Triticum aestvium 6AL RENS-GPFELSGGSPGHTAA-PPMDMRPRQP--PRSVFGAN--MNNSSTALRNMIISPVK

Triticum aestvium 6DS RENS-GPFELSGGSPGHTAA-PPMDMRPRQP--PRSVFGAN--MNNSSTALRNMIISPVK

Triticum aestvium 6BS RENS-GPFELSGGSPGHTAA-PPMDMRPRQL--PRSVFGAN--MNNSSTALRNMIISPVK

Linum usitatissimum SATSG--FGISGGNSPVNR-QAQADLGNIRA-GGNPAFGAA--PRNPAMALRNLIFSPIK

Beta vulgaris SSNSGGAFDVSAGSPSRRA-PVPIDTGNRRP-SAHPPFGSG-N-SNPSMTLRNLVLSPIK

Cucumis sativa SGNS-GHFDISVGSPAKQAAPMAMEAGNRRA-GAHTAFGSG-A-GNPSMTLRNLILSPIK

Medicago truncatula SNNS-GHFDISVGSPAKQTI-IPGDAGNRRV-GAHPVFGPG-ATSNPSMNLRNLILSPIK

Glycine max SNNS-GHFDLSVGSPAKQTV-IAGDAGNRRA-GAHPVFGPG-ATNNPSMTLRNLILSPIK

Phaseolus vulgaris SNNS-GPFDLSVVSPAKQTA-IAGDAGNRRA-GAHSVFGPG-ATNNPSMTLRNMILSPIK

Malus domestica SSNSGGPFDITLESPAKQAA-IPVDAGXRRA-GGHTMFGNG-X-SNPSXTLRNMIISPIK

Prunus persica SSNSGGPFDISVGSPAKQAA-IPVDAVNRRA-GAHPMFGTG-A-SNPSMTLRNLILSPIK

Vitis vinifera SSNSGGPFEVSGGSPMKQAR-ISVDAGNRRA-GA---FGTG-A-GNPSMTIRNLILSPIK

Eutrema salsugineum SSNS-GPFDISNDSP----A-IPSDVGNRRAGGGHPVFGGG-GTSNPQSTLRNLILSPIK

Boechera stricta SSNS-GPFDISIDSP----A-IPSDLGNRRAGGGHPVYGGG-GTSNPQSTLRNLILSPIK

Camelina sativa SSNS-GPFDISTDSP----A-IPSDLGNRRAGGGHPVYGGGGGTSNPQSTLRNLILSPIK

Capsella grandiflora SSNS-GPFDISTDSP----A-IPSDLGNRRSGGGHPVYGGG-GPSNPQSTLRNLILSPIK

Capsella rubella SSNS-GPFDISTDSP----A-IPSDLGNRRSGGGHPVYGGG-GPSNPQSTLRNLILSPIK

Arabidopsis lyrata SSNS-GPFDISTDSP----A-IPSDLGNRRAGGGHPVYGGG-GTSNPQSTLRNLILSPIK

Arabidopsis thaliana Col SSNS-GPFDISTDSP----A-IPSDLGNRRAGRGHPVYGGG-GTANPQSTLRNLILSPIK

Arabidopsis thaliana Ler SSNS-GPFDISTDSP----A-IPSDLGNRRAGGGHPVYGGG-GTANPQSTLRNLILSPIK

Eucalyptus grandis SSIS-GPFDISGGSPAKHPT-IPVDANSRRV-SGDPVFGAS--TANPSMTLRNLILSPIK

Salix purpurea SPNS-GPFDISDCSPAKQAA-MPVDFGNRRP-GSVRAFGAG--SGNPSMTLRNLILSPIK

Populus trichocarpa SSNS-GPFEIRGGSPANQAA-MPVDVGNRRI-GSVPSFGAG--SGNPSMTLRNLILSPIK

Manihot esculenta SSNS-GPFEISGGSPAKQAA-IPIDAGNRRA-GVHPAFGAG--PGNTSMTLRNLILSPIK

Citrus sinensis SSNS-GPFDIATGSPAKQPV-IPVDGGNRRA-DARPAFGGG--AANPSMTLRNLILSPIK

Theobroma cacao SSNS-GHFDISGGSPAKQAA-IPVDVGNRRG-GAHPAFGAG--GVNPAMTLRNLIISPIK

* : . : : :* * :**::.**:*

____________________________________________________________

**C-terminal domain**

**R264G Col/Ler**

Panicum halli RPQQSRNRLHMFTL--------

Setaria italica RPQQSRNRQHMFTL--------

Sorghum bicolor RPQVSRNRQHIFTL--------

Zea mays RPQTSRNRQHIFT---------

Oryza sativa RPQLSRNRPHMFTL--------

Leersia perrieri RPQLSRNRLFTDQY--------

Brachypodium distachyon RPQLSRNRPQLFTL--------

Triticum aestvium 6AL RPQ-PRNRPQMFTL--------

Triticum aestvium 6DS RPQ-PRNRPQMFTL--------

Triticum aestvium 6BS RPQ-PRNRPQMFTL--------

Linum usitatissimum RPQTR-NRNNLFS---------

Beta vulgaris RPQLSRNRPQLFTM--------

Cucumis sativa RPQLSR-RPQMFTL--------

Medicago truncatula RPQLSHCRLKEANLLCRVSYAF

Glycine max RPQLSRNRPQLFTL--------

Phaseolus vulgaris RPQLSRNRPQLFTL--------

Malus domestica RPQFSR-RPQMFT---------

Prunus persica RPQLSRNRPQMFT---------

Vitis vinifera RPQLSRGRPNMFT---------

Eutrema salsugineum RSQLSRSRPQLFTL--------

Boechera stricta RSQLSRSRPQLFTL--------

Camelina sativa RSQLSRSRPQLFTL--------

Capsella grandiflora RSQLSRSRPQLFTL--------

Capsella rubella RSQLSRSRPQLFT---------

Arabidopsis lyrata RSQLSRSRPQLFT---------

Arabidopsis thaliana Col RSQLSRSRPQLFTL--------

Arabidopsis thaliana Ler RSQLSRSRPQLFTL--------

Eucalyptus grandis RPQLSRNRTQMFT---------

Salix purpurea RPQLSRSRPQMFTL--------

Populus trichocarpa RPQLSRSRPQMFT---------

Manihot esculenta RPQLSRSRPQMFTL--------

Citrus sinensis RPQLSRNRTQIFTL--------

Theobroma cacao RPQLSRNRTQIFT---------

______________________

**C-terminal domain**
